# Network Pharmacology-Based Analysis on the Curative Effect of Kunxian Capsules against Rheumatoid Arthritis

**DOI:** 10.1155/2021/6812374

**Published:** 2021-09-29

**Authors:** Hong-Xia Yan, Chun-Fang Xu, Hong Yang, Xiao-Ya Wen, Zhi-Peng Wang, Yan-Hong Chen, Jing-Xue Liu, Wan-Sheng Chen, Shou-Hong Gao, Xia Tao

**Affiliations:** ^1^College of Traditional Chinese Medicine, Yunnan University of Chinese Medicine, Kunming, Yunnan 650500, China; ^2^Department of Pharmacy, Changzheng Hospital, Second Military Medical University, Shanghai 200003, China; ^3^College of Chemistry and Bio-Engineering, Yichun University, Yichun, Jiangxi 336000, China; ^4^Research and Development Center of Chinese Medicine Resources and Biotechnology, The Ministry of Education (MOE) Key Laboratory for Standardization of Chinese Medicines, Institute of Chinese Materia Medica, Shanghai University of Traditional Chinese Medicine, Shanghai 201203, China

## Abstract

Kunxian capsules (KCs), a Chinese patent medicine, have been clinically proven to be effective in the treatment of rheumatoid arthritis (RA). However, the chemical profile of KC remains to be characterized, and the mechanism underlying the protective effect against RA is yet to be elucidated. Here, a network pharmacology-based approach was adopted, integrated with the chemical profiling of KC by UHPLC-Q-TOF/MS. As a result, a total of 67 compounds have been identified from KC extract, among which 43 were authenticated by comparison to the mass spectrum of standard chemicals. ADME behaviors of the chemical constituents of KC were predicted, resulting in 35 putative active ingredients. Through target prediction of both active ingredients of KC and RA and PPI analysis, core targets were screened out, followed by biological process and related pathway enrichment. Then, a TCM-herb-ingredient-target-pathway network was constructed and a multicomponent, multitarget, and multipathway synergistic mechanism was proposed, providing an information basis for further investigation. The active pharmaceutical ingredients included mainly terpenoids (such as triptolide and celastrol), sesquiterpene pyridines (such as wilforgine and wilforine), and flavonoids (such as icariin, epimedin A, B, and C, and 2″-O-rhamnosylicariside II).

## 1. Introduction

Rheumatoid arthritis (RA), a chronic autoimmune disease, causes progressive articular destruction and associated comorbidities in vascular, metabolic, bone, and psychological domains [1]. The disease is characterized by synovial inflammation and hyperplasia (“swelling”), autoantibody production, cartilage and bone destruction (“deformity”), and systemic features, including cardiovascular, pulmonary, psychological, and skeletal disorders [1, 2]. With the aggravation of population aging, the incidence of RA has increased from 0.5 to 1% worldwide. Women are more vulnerable to the disease than men, and those aged 50 to 60 are with relatively higher risk [1]. Currently, the treatment options for RA included non-steroidal anti-inflammatory drugs (NSAIDs), antirheumatic drugs, glucocorticoid, and immunomodulators. However, adverse effects, even drug-induced diseases, were frequently reported with single-target and symptom-based medications, especially with glucocorticoid [3]. Obviously, current one drug-one target-one disease approaches in drug discovery have become increasingly inefficient [4]. Multiple synergistic medications are in great need.

Despite that the origin of RA is unknown, it has become recently evident that RA arises based on genetic and epigenetic factors, with the environment playing an important role [5]. According to the Kyoto Encyclopedia of Genes and Genomes (KEGG) database, a large and complex network involving T cells, B cells, macrophages, and various cytokines or chemokines, such as IL-1, IL-6, IL-17, TNF-*α*, and MMPs, participated in the pathogenetic processes of RA. Like cocktail therapy, ethnic herbal medicine tended to provide a multitarget approach against illnesses and ailments.

Kunxian capsules (KCs), belonging to the Class VI New Medications in State Key Task 95 in China, are made from 4 crude drugs, including *Tripterygium hypoglaucum* (Lévl.) Hutch. (TH), *Epimedium brevicornu* Maxim. (EB), *Cuscuta chinensis* Lam. (CC), and *Lycium barbarum* L. (LB). KC has been demonstrated to have immunomodulatory, cytokine-antagonistic, anti-inflammatory, and analgesic effects, without glucocorticoid-like effect to induce the atrophy of immune organs [6]. KC treatment was shown to bring significant mitigating effect in patients with RA, systemic lupus erythematosus, or ankylosing spondylitis [6–10]. However, the pharmacodynamic material basis and the underlying mechanisms of the curative effect of KC remain to be illustrated, largely limiting the global acceptance of KC.

Ultra-high performance liquid chromatography coupled with hybrid triple quadrupole time-of-flight mass spectrometry (UHPLC-Q-TOF/MS) is a high-throughput analytical technology that has rapidly developed in the past decade and is widely used in the fields of environmental science, medicine, drug research, and others [11]. Firstly proposed by Hopkins in 2008 [12], network pharmacology represented an efficient approach to establish relationships between multiple compounds and various targets. Therefore, network pharmacology was thought to be appropriate to investigate the molecular mechanisms of complex systems, such as natural herbs or Traditional Chinese Medicine (TCM) [13]. Based on the network of herbs, targets, and diseases, this strategy could systemically elucidate the mechanisms in a holistic manner, matching the TCM theory intrinsically and providing a new and effective way of the research of Chinese compound formulas [13]. Therefore, the current study aimed to investigate the mechanisms underlying the curative effect of KC against RA, through a network pharmacology-based approach integrated with chemical characterization by UHPLC-Q-TOF/MS. The schematic workflow is shown in Figure S1.

## 2. Materials and Methods

### 2.1. Materials and Reagents

Reference substances of hyperoside, triptolide, epimedin C, icariin, and baohuoside I were purchased from the National Institutes for Food and Drug Control (Beijing, China). Arginine, proline, valine, betaine, chlorogenic acid, catechin, L-epicatechin, rutin, isoquercitrin, luteolin-7-O-glucoside, kaempferitrin, astragalin, quercitrin, daidzein, epimedoside A, quercetin, luteolin, epimedin A1, epimedin A, epimedin B, kaempferol, isorhamnetin, icaritin, baohuoside II, sagittatoside A, sagittatoside B, wilforgine, wilforine, 2″-O-rhamnosylicariside II, demethylzeylasteral, celastrol, and baohuoside I were purchased from Dalian Meilun Biotechnology Co. Ltd. (Dalian, China). Epigallocatechin, procyanidin B-3, magnoflorine, wilfortrine, euonine, and wilfornine A were purchased from Chengdu DeSiTe Biotechnology Co. Ltd. (Chengdu, China). The purity of each compound was determined to be higher than 94% by HPLC. LC-grade acetonitrile, methanol, and formic acid were purchased from Merck (Darmstadt, Germany). Purified water for experiments was purchased from A.S. Watson Group Ltd. (Hong Kong, China). Kunxian capsules (KCs) were provided by the Guangzhou Chen Li Ji Pharmaceutical Co. Ltd. (Guangzhou, China).

### 2.2. Preparation of Standard and Sample Solutions

Stock solutions of 43 standards were prepared at a concentration of 1 mg/mL in methanol. Next, aliquots of each stock solution were mixed and diluted with methanol to achieve a series of standard working solutions. The KC samples were ground to a fine powder. Then powdered KC of 0.150 g was suspended with 20 mL of 70% methanol-water (v/v) and weighed, and it underwent ultrasonic-assisted extraction (40 kHz, 500 w) for 15 min, followed by lost weight replenishment. Then, the extracted solution was centrifuged at 15,000 rpm for 20 min, and the supernatant was obtained as sample for detection. All the samples were stored at 4°C before analysis.

### 2.3. UHPLC-Q-TOF/MS Analysis

The LC/MS system consisted of an Agilent 1290 Infinity LC system coupled to an Agilent 6530 UHD accurate-mass quadrupole time-of-flight (Q-TOF) mass spectrometer (Agilent, USA). Chromatographic separations were performed on Accucore C18 (150 × 2.1 mm, 2.6 *μ*m, Thermo Fisher Scientific, USA) maintained at 35°C. The chromatographic conditions were as follows: flow rate of 0.3 mL/min, sample injection volume of 1 *µ*L, mobile phase A (0.05% formic acid-water), and mobile phase B (0.05% formic acid-acetonitrile) with a gradient elution program as follows: 0–3 min, 5–12% B; 3–10 min, 12–18% B; 10–20 min, 18–25% B; 20–25 min, 25–37% B; 25–37 min, 37–65% B; 37–38 min, 65–95% B; and 38–40 min, 95% B.

An electrospray ionization source (ESI) interface was used, and the following parameters were employed in positive modes: capillary voltage, 4.5 kV; nebulizer pressure, 50 psi; drying gas flow, 11 L/min; gas temperature, 350°C; and scan range, 100–1500 m/z. The collision energy was set at 30 eV to obtain the fragment ion data. Data were collected in centroid mode, and the mass range was set at m/z 100–1100 [14–16]. All the acquisition and analysis of data were controlled by Agilent MassHunter Workstation Software Qualitative Analysis Version B.10.0 (Agilent Technologies, USA).

### 2.4. Identification of the Main Ingredients in KC

The compounds contained in the 4 crude drugs were gathered from multiple databases, including PubMed, SciFinder (scifinder.cas.org/), and TCMSP (tcmspw.com/tcmsp.php), as well as subsequent literature search. A KC-specific in-house library was established, including the basic information of the compounds: name, formula, and exact molecular weight. Agilent MassHunter Workstation for Qualitative Analysis (Version 10.0) was employed to analyze the components of KC, which provided a list of compounds that were putatively contained in KC. Chemical standards were obtained and used for the final authentication of the components.

### 2.5. Target Fishing of KC and RA

To screen out putative active ingredient, TCMSP database and SwissADME (swisstargetprediction.ch/) were added to predict the absorption, distribution, metabolism, and excretion (ADME) properties, focusing on 3 aspects, including oral bioavailability (OB), druglikeness (DL), and intestinal permeability. To predict related targets, TCMSP database and SwissTargetPrediction web tool were utilized to gather the potential targets of the screened-out active ingredients. A list of potential targets of the active ingredients of KC was obtained after duplicate removal. GeneCards database (genecards.org/) was used to predict the related targets to RA. The first 1000 targets with higher relevance score were preserved for further analysis. The overlapping targets between the KC and RA were extracted using Microsoft Excel and visualized using Venny2.1.0 (https://bioinfogp.cnb.csic.es/tools/venny/index.html).

### 2.6. PPI Analysis for Core Target Screening

STRING database (string-db.org/) was employed for the PPI analysis with the organism set as “*Homo sapiens*.” The resulting file (.tsv) was introduced to Cytoscape 3.7.0 for topological analysis and visualization. The threshold for core target screening was that the “degree,” “betweenness,” and “closeness” were all larger than the averages of the whole sample.

### 2.7. GO and KEGG Pathway Enrichment

After core target screening, R package from Bioconductor database (bioconductor.org) was adopted to conduct GO and KEGG enrichment based on OmicShare platform (omicshare.com/tools/index.php/) in 3 modules, including biological process (BP), molecular function (MF), and cellular component (CC). *P* < 0.05 and FDR <0.05 were used as screening criteria. Cytoscape 3.7.0 software was adopted to visualize the herb-ingredient-target-pathway network of KC, active ingredients, core targets, and key pathways.

## 3. Results

### 3.1. Identification of the Chemical Constituents in KC Extract by UHPLC-Q-TOF/MS

**Figure 1:**
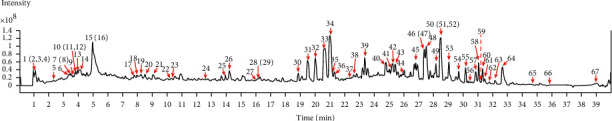
Representative total ion current (TIC) chromatogram of KC extract by UHPLC-Q-TOF/MS in positive mode.

Through UHPLC-Q-TOF/MS analysis, 67 chemical constituents were identified from the 70% methanol-in-water extract of KC according to mass fragmentation patterns based on a previously established KC-specified in-house library (Figure 1). Among them, 43 compounds (1, 2, 3, 4, 5, 8, 10, 11, 15, 16, 17, 18, 19, 20, 21, 22, 23, 25, 26, 27, 28, 29, 30, 31, 32, 33, 34, 35, 37, 43, 45, 47, 48, 50, 51, 52, 55, 58, 60, 63, 64, 66, and 67) were authenticated by comparison to the mass spectrums of the corresponding chemical standards, as indicated by a star symbol (^*∗*^) in Table S1. The other 24 ingredients were identified by studying on the mass fragmentation patterns through analysis of the mass spectra, as well as by referring to previous reports [17–34]. As a result, 67 compounds were identified in total, including 32 flavonoids, 20 alkaloids (18 sesquiterpene alkaloids), 6 amides, 5 terpenoids, and 4 others.

### 3.2. Screening of the Potential Active Ingredients

**Figure 2:**
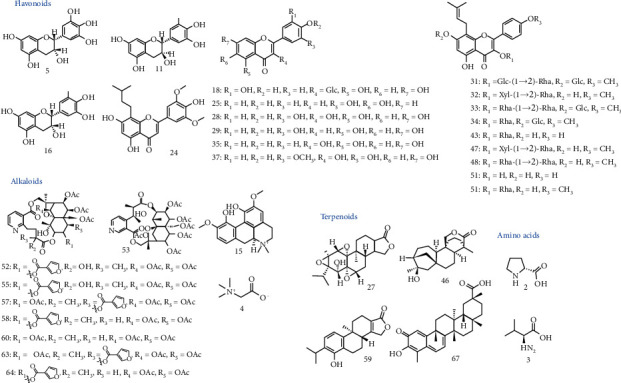
The structures of the 35 potentially active ingredients in KC.

Active ingredients were screened out by analyzing their behaviors of absorption, distribution, metabolism, and excretion (ADME) by referring to TCMSP database and/or prediction using SwissADME web tool. The inclusion of potential active ingredients generally complied with “Rule of Five” (Ro5), firstly proposed by Lipinski et al. in 1997 [35]. Specifically, the thresholds were as follows: for TCMSP database, oral bioavailability (OB) should be no lower than 30%, druglikeness (DL) should be no lower than 0.18%, and intestinal permeability (Caco-2) should be no lower than −0.40 [36]; for SwissADME analysis, gastrointestinal absorption (GI) should be indicated as “high,” and DL analysis should result in more than 2 “Yes,” with data shown in Table S2 (In total, 5 filters of rule-of-five were adopted in the study, including Lipinski (Pfizer), Ghose (Amgen), Veber (GSK), Egan (Pharmacia,) and Muegge (Bayer) methods. One “Yes” was counted if no violations were found in one filter. The times of found “Yes”s were presented as results [37] (see swissADME.ch.)). Moreover, notwithstanding their predicted compromised ADME behaviors, some other compounds with reported strong pharmacological activities were also preserved for further analysis, such as epimedin A [38, 39], 2″-O-rhamnosylicariside II (rha-icariside) [40], and wilfornine A [23]. Finally, 35 active ingredients stood out as presented in Table 1 and Figure 2, including 19 flavonoids (3 flavones, 4 flavonols, and 3 flavanes), 10 alkaloids (8 sesquiterpene pyridine alkaloids), 4 terpenoids, and 2 amino acids.

### 3.3. Target Fishing and Major Hub Analysis

**Figure 3:**
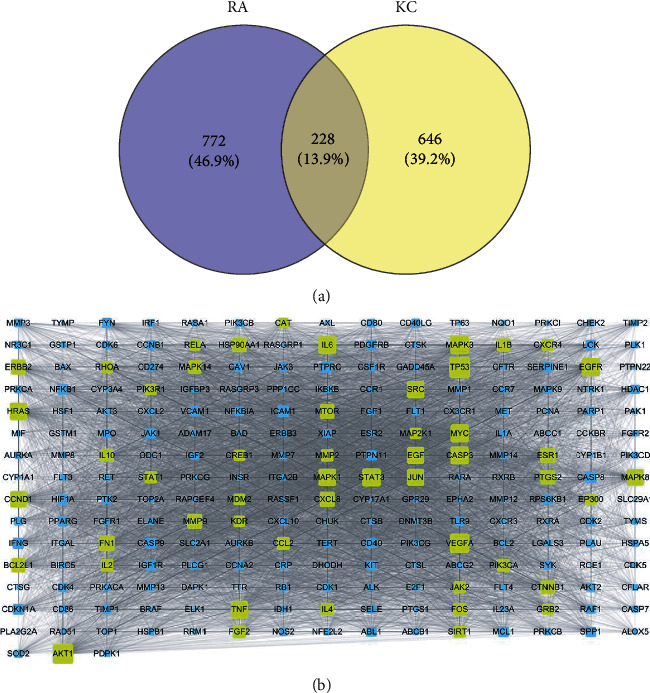
Prediction of key targets. Venn diagram (a) and the PPI network (b).

Target fishing was conducted for the 35 candidate compounds by searching the PubChem and TCMSP databases or by virtual prediction through SwissTargetPrediction web tool. UniProt Consortium was employed to convert the targets to uniform gene symbol. Finally, merging and duplicate removal yielded 874 potential targets for the 35 active ingredients in KC (Table S3). Using GeneCards database, the first 1000 relevant targets for RA were obtained (Table S4). Then, 228 overlapping targets (Table S5) between KC and RA (Figure 3(a)) were introduced into STRING database for PPI analysis. A PPI network with 228 nodes and 14356 edges (Figure 3(b)) was constructed. Subsequent topological analysis by Cytoscape software presented 51 major hubs that might play a potential important role in the curative effect of KC for RA (Table S6).

### 3.4. GO and KEGG Enrichment

**Figure 4:**
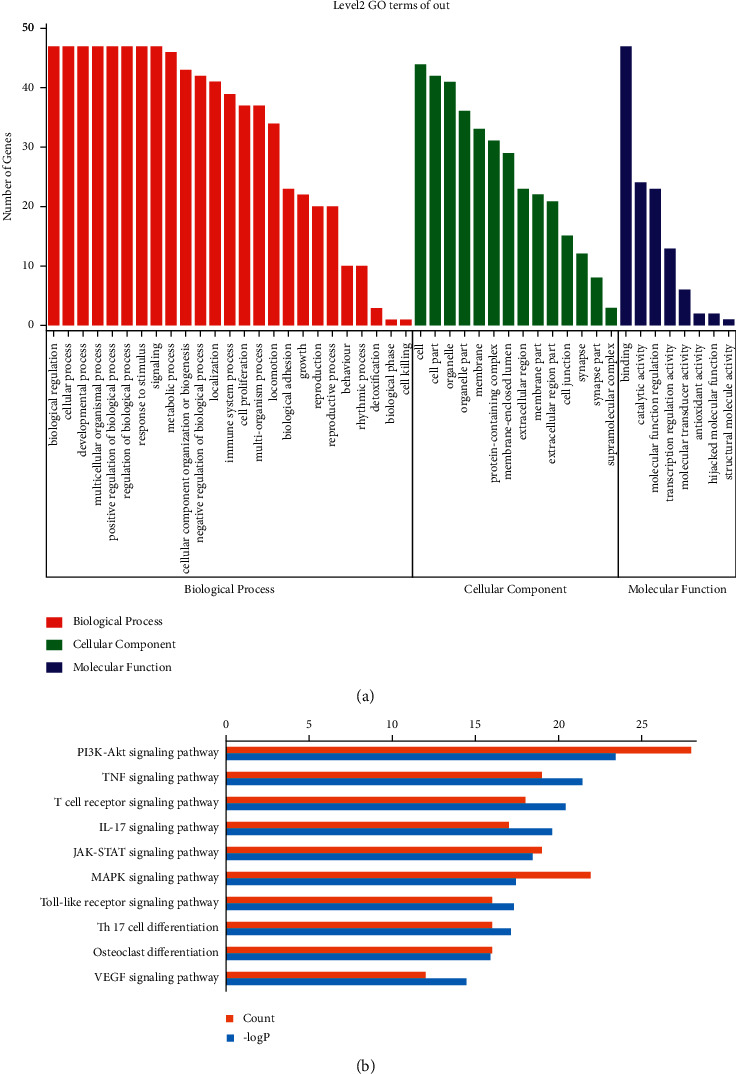
GO and KEGG enrichment. (a) The relevant biological processes, cellular components, and molecular functions. (b) The top 10 related pathways.

GO enrichment of the core targets indicated that multiple biological processes (BP), cellular components (CC), and molecular functions (MF) have been engaged in the mechanisms underlying the protective effect of KC against RA (Figure 4(a)). The top 10 enriched BP included biological regulation, cellular process, developmental process, multicellular organismal process, positive regulation of biological process, regulation of biological process, response to stimulus, signaling, metabolic process, and cellular component organization or biogenesis. The top 10 related CC included cell, cell part, organelle, organelle part, membrane, protein-containing complex, membrane-enclosed lumen, extracellular region, membrane part, and extracellular region part. The top 3 related MF included binding, catalytic activity, and molecular function regulator. After pathway enrichment using KEGG, the top 10 relevant pathways are shown in Figure 4(b), including PI3K-Akt signaling pathway, TNF signaling pathway, T cell receptor signaling pathway, IL-17 signaling pathway, JAK-STAT signaling pathway, MAPK signaling pathway, Toll-like receptor signaling pathway, Th17 cell differentiation, and VEGF signaling pathway.

### 3.5. Construction of TCM-Herb-Ingredient-Target-Pathway Network

**Figure 5:**
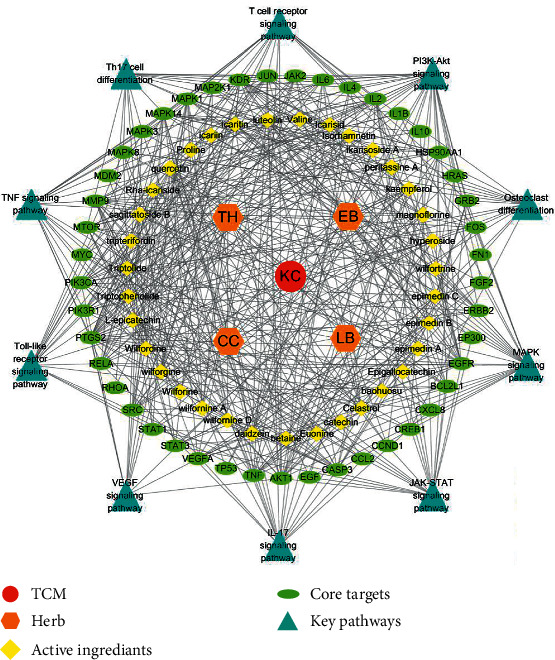
Construction of TCM-herb-ingredient-target-pathway network.

**Figure 6:**
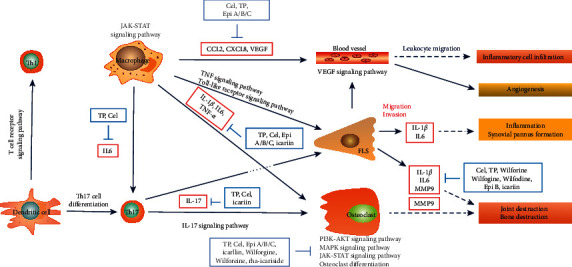
The potential mechanism underlying the protective effect of KC against RA. Note: Cel, celastrol; TP, triptolide; epi A/B/C, epimedin A/B/C; rha-icariside, 2″-O-rhamnosylicariside II.

Through literature research, we linked up the aforementioned 10 pathways, key targets, and active ingredients, constructed a TCM-herb-ingredient-target-pathway network (Figure 5), and proposed a possible mechanism underlying the curative effect of KC against RA (Figure 6). The network contained 95 nodes and 537 edges, with specific information shown in Table s7.

## 4. Discussion

The current work investigated the potential mechanisms of the curative effect of KC, a Chinese patent medicine, against RA, by integrating the chemical profiling with network pharmacology analysis, for the first time [6, 41]. The establishment of TCM-herb-ingredient-target-pathway network could make a useful reference for further drug development and the treatment of RA. The efficacy of KC against RA has been proved by multicenter clinical trials [6–10]. By analyzing open-access databases, Tang et al. collected related information of KC and constructed a disease network for the protection of KC against RA [6], whereas no chemical characterization was conducted. Jing et al. investigated the potential mechanism of KC against proteinuria and identified 51 chemical compounds from KC, among which 9 compounds were compared with corresponding reference standards [41]. Instead, our study identified 67 compounds from KC extract, and 43 components were authenticated by reference standard comparison.

The pharmacodynamic material basis was investigated in our study. Based on predicted ADME behaviors and previous reports, 35 potentially active ingredients were screened out. Among those compounds, 8 sesquiterpene pyridine alkaloids (52, 53, 55, 57, 58, 60, 63, and 64) were demonstrated to have immunosuppressive effect [23, 42], which originated from TH, one of the principal herbs in KC. As the principle sesquiterpene pyridines, wilforgine (58) and wilforine (64) were reported to exhibit immunosuppressive and anti-inflammatory effects [42]. Besides, 2 terpenoids, triptolide (27) and celastrol (67), seemed to be worth our attention. Triptolide was reported to suppress activation of inflammation, thus inhibiting bone loss (osteopenia) [43]. Fan et al. investigated the potential mechanism underlying which triptolide exerted therapeutic effect against RA, by combining bioinformatics analysis with experiment validation [44]. They highlighted that triptolide could inhibit inflammatory responses in RA by triggering receptors expressed on myeloid cells related to (TREM)-1 signaling pathway [44]. Moreover, it was found that in vitro and in vivo, triptolide treatment significantly reduced the migratory and invasive capacities of fibrolast-like synoviocytes (FLS), which would have tumor-like features when activated [45]. FLS, a major constituent of the synovial hyperplasia, could play a pivotal role in RA invading cartilage and bone tissue [46–48]. The related effector molecules to FLS, including inflammatory factors and MAPKs, have also emerged as important mediators of RA [46]. Celastrol, also known as tripterine, also has various pharmacological functions. Li et al. [49] demonstrated that tripterine could protect a chondrogenic cell line ATDC5 from lipopolysaccharide (LPS) injury in vitro. Besides, celastrol induced autophagy-dependent cytotoxicity in synovial fibroblasts (the primary form of FLS) and repressed arthritis in vivo, possibly through blockage of calcium signaling [50]. The other principal herb, EB, provided 15 putative active ingredients, including 14 flavonoids. Icariin (34), one of the main flavonoids, was shown to alleviate RA in a murine model [51] and to inhibit proliferation and inflammation, promoting apoptosis of FLS [52]. Besides, 3 flavonoids, epimedin A (31), B (32), and C (33), and 2″-O-rhamnosylicariside II (rha-icariside, 48) showed the most profound anabolic and anti-inflammatory effects on human osteoarthritic chondrocytes [38, 39, 53].

As depicted in KEGG database and Figures 4(b)–6, the pathogenesis of RA might involve multiple immune and inflammatory cells, such as dendric cells (DC), T cells, macrophages, and effector cells, including chondrocytes, osteoclast, FLS, and vessel cells. Several signaling pathways were supposed to be involved in the process. T cell receptor signaling pathway participated in the differentiation of self-reactive Th1 cell from DC. The differentiation of Th17 cell and the secretion of IL-17 were supposed to be the early activator of T cell-mediated inflammatory responses. These three pathways represented early promoter of the occurrence of RA. Then, signaling pathways of JAK-STAT, PI3K-AKT, and MAPK were involved in the osteoclast differentiation, thus leading to bone and joint destruction. FLS could be activated through TNF signaling pathway and Toll-like receptor signaling pathway, thereby promoting the inflammation. Besides, VEGF signaling pathway could be involved in the leukocyte migration and inflammatory cell infiltration.

It was suggested that KC exerted its efficacy against RA through a complex network that involved various components, multiple pathways, and many targets. First and foremost, KC could directly intervene the enhanced differentiation and activation of osteoclasts, thus ameliorating local bone erosion and systemic osteoporosis in RA [54]. Triptolide has been shown to inhibit the phosphorylation of PI3K and AKT in bone marrow mononuclear cells [43], to decrease the phosphorylation of JAK and STAT in the human leukemic U937 cells and in collagen-induced arthritis rats [44], and to inhibit the activation of matrix metalloproteinase 9 (MMP-9). Moreover, KC could inhibit the excessive inflammatory response and regulate the immune reaction [6]. Icariin inhibited STAT3 activation in T cells, resulting in decreased IL-17 production and alleviated RA [51]. Moreover, IL-6, IL-1*β*, and TNF-*α* were vital pathogenicity cytokines, which could be reduced by components of TH [52, 53, 55]. Other involved chemokines included CCLs and CXCLs [38]. Other than those targets, VEGF was involved in the generation of pannus, an inflammatory exudate overlying synovial cells commonly occurring in RA.

## 5. Conclusions

In conclusion, KC possibly attenuated RA through a multicomponent, multitarget, and multipathway synergistic mechanism. It was suggested that the pharmacodynamic material basis included terpenoids and sesquiterpene pyridines from *Tripterygium hypoglaucum*, such as triptolide, celastrol, wilforgine, and wilforine, and flavonoids from *Epimedium brevicornu*, such as icariin, epimedin A, B, and C, and 2″-O-rhamnosylicariside II. At last, further in-depth studies are still needed to verify the proposed pharmacodynamic material basis and related pathways.

## Figures and Tables

**Table 1:** The 35 putative active ingredients in KC.

Peak no.	Name	Formula	CAS	Sources
2	Proline	C_5_H_9_NO_2_	344-25-2	CC, LB
3	Valine	C_5_H_11_NO_2_	72-18-4	CC, LB
4	Betaine	C_5_H_11_NO_2_	107-43-7	LB
5	Epigallocatechin	C_15_H_14a_O_7_	970-74-1	TH
11	Catechin	C_15_H_14_O_6_	7295-85-4	TH, CC
15	Magnoflorine	C_20_H_23_NO_4_	2141-09-5	EB
16	L-Epicatechin	C_15_H_14_O_6_	490-46-0	TH, CC
18	Hyperoside	C_21_H_20_O_12_	482-36-0	EB, CC
24	Baohuosu	C_22_H_22_O_7_	119730-90-4	EB
25	Daidzein	C_15_H_10_O_4_	486-66-8	EB, CC
27	Triptolide	C_20_H_24_O_6_	38748-32-2	TH
28	Quercetin	C_15_H_10_O_7_	117-39-5	EB
29	Luteolin	C_15_H_10_O_6_	491-70-3	EB, CC
31	Epimedin A	C_39_H_50_O_20_	140147-77-9	EB
32	Epimedin B	C_38_H_48_O_19_	110623-73-9	EB
33	Epimedin C	C_39_H_50_O_19_	110642-44-9	EB
34	Icariin	C_33_H_40_O_15_	489-32-7	EB
35	Kaempferol	C_15_H_10_O_6_	520-18-3	CC
37	Isorhamnetin	C_16_H_12_O_7_	480-19-3	CC
43	Baohuoside II/ikarisoside A	C_26_H_28_O_10_	55395-07-8	EB
46	Tripterifordin	C_20_H_30_O_3_	139122-81-9	TH
47	Sagittatoside B	C_32_H_38_O_14_	118525-36-3	EB
48	2″-O-Rhamnosylicariside II (rha-icariside)	C_33_H_40_O_14_	135293-13-9	EB
50	Icaritin	C_21_H_20_O_6_	118525-40-9	EB
51	Baohuoside I/icarisid	C_27_H_30_O_10_	113558-15-9	EB
52	Wilfortrine	C_41_H_47_NO_20_	37239-48-8	TH
53	Peritassine A	C_38_H_47_NO_18_	150881-01-9	TH
55	Wilfordine	C_43_H_49_NO_19_	37239-51-3	TH
57	Wilfornine D	C_43_H_49_NO_21_	NA	TH
58	Wilforgine	C_41_H_47_NO_19_	37239-47-7	TH
59	Triptophenolide	C_20_H_24_O_3_	74285-86-2	TH
60	Euonine	C_38_H_47_NO_18_	41758-69-4	TH
63	Wilfornine A	C_45_H_51_NO_20_	345954-00-9	TH
64	Wilforine	C_43_H_49_NO_18_	11088-09-8	TH
67	Celastrol	C_29_H_38_O_4_	34157-83-0	TH

*Note*. TH, *Tripterygium hypoglaucum* (Lévl.) Hutch.; EB, *Epimedium brevicornu* Maxim.; CC, *Cuscuta chinensis* Lam.; LB, *Lycium barbarum* L.

## Data Availability

The data used to support the findings of this study are available from the corresponding author upon request.

## Abbreviations

NSAIDs: Non-steroidal anti-inflammatory drugs
KC: Kunxian capsule
RA: Rheumatoid arthritis
TCM: Traditional Chinese Medicine
TH: *Tripterygium hypoglaucum* (Lévl.) Hutch.
EB: *Epimedium brevicornu* Maxim.
CC: *Cuscuta chinensis* Lam.
LB: *Lycium barbarum* L.
TIC: Total ion current
ESI: Electrospray ionization mass spectrometry
TCMSP: Traditional Chinese Medicine Systems Pharmacology Database and Analysis Platform
ADME: Absorption, distribution, metabolism, and excretion
PPI: Protein-protein interaction
GO: Gene Ontology
KEGG: Kyoto Encyclopedia of Genes and Genomes
FDR: False discovery rate
OB: Oral bioavailability
DL: Druglikeness
GI: Gastrointestinal
CAS: Chinese Academy of Sciences
Cel: Celastrol
TP: Triptolide
epi A/B/C: Epimedin A/B/C
rha-icariside: 2″-O-Rhamnosylicariside II.

## Supplementary Materials

Supplementary Materials Figure S1: graphical abstract. Table S1: peak information of the TIC chromatogram. Table S2: the predicted ADME behaviors of the 67 ingredients in KC. Table S3: target prediction of the putative active ingredients. Table S4: target prediction of RA. Table S5: overlapping of the predicted targets of ingredients and RA. Table S6: the key targets and their topological parameters by PPI. Table S7: nodes and edges in the TCM-herb-ingredient-target-pathway network.
