# Supplementary material for: Network Pharmacology-Based Analysis on the Curative Effect of Kunxian Capsules against Rheumatoid Arthritis
Source: Evid Based Complement Alternat Med. 2021 Sep 29;2021:6812374. doi: 10.1155/2021/6812374 (PMC8497097; doi:10.1155/2021/6812374)
Supplement: Supplementary Materials — Figure S1: graphical abstract. Table S1: peak information of the TIC chromatogram. Table S2: the predicted ADME behaviors of the 67 ingredients in KC. Table S3: target prediction of the putative active ingredients. Table S4: target prediction of RA. Table S5: overlapping of the predicted targets of ingredients and RA. Table S6: the key targets and their topological parameters by PPI. Table S7: nodes and edges in the TCM-herb-ingredient-target-pathway network. [file 6812374.f1.docx]

Figure S1. Graphical abstract.


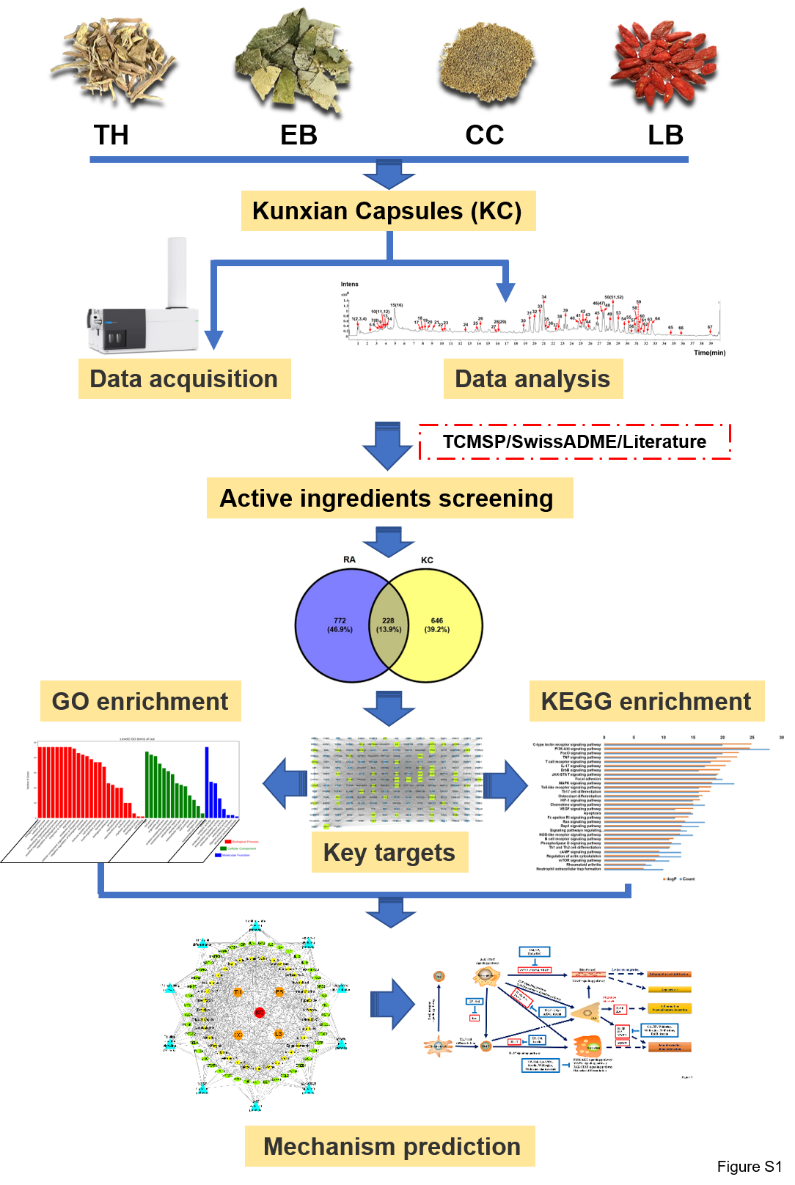


Table S1. Peak informations of the TIC chromatogram.

| **Peak No.** | **Rt (min)** | **Formula** | **Ion source** | **Observed mass (*m/z*)** | **Error (ppm)** | **Fragment ions (m/z)** | **Name** | **Source** | **Compound class** |
| --- | --- | --- | --- | --- | --- | --- | --- | --- | --- |
| 1* | 1.013 | C_6_ H_14_ N_4_ O_2_ | M+H | 175.1192 | -0.98 | 158.0930, 60.0652 | arginine | CC, LB | others |
| 2* | 1.015 | C_5_ H_9_ N O_2_ | M+H | 116.0714 | 8.58 | 133.0984, 55.0544, 70.0653 | proline | CC, LB | others |
| 3* | 1.024 | C_5_ H_11_ N O_2_ | M+H | 118.0867 | 2.99 | 55.0544, 70.0651, 101.9497 | valine | CC, LB | others |
| 4* | 1.029 | C_5_ H_11_ N O_2_ | M+H | 118.0867 | 2.99 | 58.0652 | betaine | LB | alkaloids |
| 5* | 2.217 | C_15_H_14_O_7_ | M+H | 307.0827 | 3.07 | 139.036 | epigallocatechin | TH | flavonoids |
| 6 | 3.363 | C_37_ H_54_ N_3_ O_16_ | M+H | 796.3499 | -0.25 | 634.2961, 310.2084, 200.0932 | lycibarbarspermidine E | LB | amides |
| 7 | 3.431 | C_37_ H_55_ N_3_ O_16_ | M+H | 798.3651 | -0.76 | 636.3111, 347.0962, 200.0921 | lycibarbarspermidine L or K | LB | amides |
| 8* | 3.448 | C_30_ H_26_ O_12_ | M+H | 579.1492 | -0.14 | 447.0374, 409.0900, 257.0424, 200.0927 | procyanidin B-3 | TH | flavonoids |
| 9 | 3.617 | C_37_ H_55_ N_3_ O_16_ | M+H | 798.3651 | -0.76 | 636.3111, 347.0962, 200.0921 | lycibarbarspermidine M | LB | amides |
| 10* | 3.701 | C_16_ H_18_ O_9_ | M+H | 355.1013 | -1.59 | 200.9991, 193.0379, 163.0379 | chlorogenic acid | CC, LB | others |
| 11* | 3.769 | C_15_ H_14_ O_6_ | M+H | 291.0853 | -3.02 | 139.0377, 123.0427 | catechin | TH, CC | flavonoids |
| 12 | 3.8 | C_37_ H_54_ N_3_ O_16_ | M+H | 796.3499 | -0.25 | 634.2961, 310.2084, 200.0932 | lycibarbarspermidine F | LB | amides |
| 13 | 3.908 | C_31_ H_46_ N_3_ O_11_ | M+H | 636.3106 | -3.13 | 474.2581, 384.1633, 222.1109, 310.2108 | lycibarbarspermidines H or I or J | LB | amides |
| 14 | 4.158 | C_31_ H_44_ N_3_ O_11_ | M+H | 634.2961 | -0.67 | 472.2471, 220.0954, 310.2115 | lycibarbarspermidine A or B or C or D | LB | amides |
| 15* | 4.8 | C_20_ H_23_ N O_4_ | M+H | 342.1693 | -1.64 | 297.1113, 265.0852, 237.0899, 219.0793, 58.0641 | magnoflorine | EB | alkaloids |
| 16* | 4.936 | C_15_ H_14_ O_6_ | M+H | 291.085 | -5.45 | 139.0373, 123.0422 | L-epicatechin | TH, CC | flavonoids |
| 17* | 7.839 | C_27_ H_30_ O_16_ | M+H | 611.16211 | 1.47 | 340.9951, 303.0486 | rutin | EB, LB | flavonoids |
| 18* | 7.945 | C_21_ H_20_ O_12_ | M+H | 465.1024 | -1.41 | 340.9959, 303.0494, 257.0457, 229.0504 | hyperoside | EB, CC | flavonoids |
| 19* | 8.3 | C_21_ H_20_ O_12_ | M+H | 465.1035 | 0.48 | 303.0498, 340.9971, 287.0540 | isoquercitrin | CC | flavonoids |
| 20* | 8.631 | C_21_H_20_O_11_ | M+H | 449.1070 | -3.11 | 287.0548 | luteolin-7-O-glucoside | EB | flavonoids |
| 21* | 9.129 | C_27_ H_30_ O_14_ | M+H | 579.1707 | -0.4 | 545.0938, 433.1134, 325.0021, 287.0543 | kaempferitrin | EB | flavonoids |
| 22* | 10.194 | C_21_ H_20_ O_11_ | M+H | 449.1084 | 0 | 325.0017, 287.0551, 185.0468 | astragalin | CC | flavonoids |
| 23* | 10.248 | C_21_H_20_O_11_ | M+H | 449.1084 | 0 | 303.0489, 153.0175 | quercitrin | CC | flavonoids |
| 24 | 12.549 | C_22_ H_22_ O_7_ | M+H | 399.1426 | -2.87 | 355.1176 | baohuosu | EB | flavonoids |
| 25* | 13.898 | C_15_ H_10_ O_4_ | M+H | 255.0647 | -2.06 | 227.0761 | daidzein | EB, CC | flavonoids |
| 26* | 14.185 | C_32_ H_38_ O_15_ | M+H | 663.228 | -1.01 | 517.1699, 355.1176, 299.0547 | epimedoside A | EB | flavonoids |
| 27* | 15.982 | C_20_H_24_O_6_ | M+H | 361.1664 | 3.6 | 297.1484, 343.1544 | triptolide | TH | terpenoids |
| 28* | 16.096 | C_15_ H_10_ O_7_ | M+H | 303.0495 | -1.44 | 229.0530, 153.0174, 257.0444 | quercetin | EB | flavonoids |
| 29* | 16.148 | C_15_ H_10_ O_6_ | M+H | 287.055 | -1.19 | 153.0188 | luteolin | EB, CC | flavonoids |
| 30* | 18.835 | C_39_ H_50_ O_20_ | M+H | 839.2964 | -1.01 | 677.2431, 531.1864, 369.1342, 313.0721, 331.0999 | epimedin A1 | EB | flavonoids |
| 31* | 19.495 | C_39_ H_50_ O_20_ | M+H | 839.2976 | -0.3 | 677.2431, 531.1864, 369.1342, 313.0721, 331.0999 | epimedin A | EB | flavonoids |
| 32* | 20.013 | C_38_ H_48_ O_19_ | M+H | 809.2871 | 0.14 | 677.2439, 531.1859, 369.1333, 313.0704 | epimedin B | EB | flavonoids |
| 33* | 20.628 | C_39_ H_50_ O_19_ | M+H | 823.301 | -0.59 | 677.2435, 531.1850, 369.1331, 313.0702 | epimedin C | EB | flavonoids |
| 34* | 21 | C_33_ H_40_ O_15_ | M+H | 677.2427 | -2.66 | 553.2673, 531.1851, 369.1324, 553.1673, 313.0709 | icariin | EB | flavonoids |
| 35* | 21.114 | C_15_H_10_O_6_ | M+H | 287.0549 | -2.09 | 309.0302 | kaempferol | CC | flavonoids |
| 36 | 21.964 | C_27_ H_32_ O_11_ | M+H | 533.2008 | -1.55 | 387.1441, 369.1331, 313.0709, 425.0906, 409.1225, 214.9167, 531.1852 | icaritin-3-O-rhamnoside | EB | flavonoids |
| 37* | 22.238 | C_16_ H_12_ O_7_ | M+H | 317.0667 | 2.61 | 302.0438, 285.0393, 257.0437 | isorhamnetin | CC | flavonoids |
| 38 | 22.543 | C_36_ H_45_ N O_18_ | M+H | 780.271 | 0.24 | 762.2575, 720.2478, 214.9171, 172.9313, 194.0788 | tripfordine A | TH | alkaloids |
| 39 | 23.333 | C_39_ H_48_ O_19_ | M+H | 821.2861 | -1.56 | 531.1864, 369.1330, 313.0741 | carbonly-2''-β-L-quinovosyl icariin | EB | flavonoids |
| 40 | 24.754 | C_31_ H_36_ O_14_ | M+H | 633.2161 | -1.65 | 531.1865, 385.1281, 369.1945, 313.0712 | ikarisoside F | EB | flavonoids |
| 41 | 25.228 | C38 H47 N O19 | M+H | 822.2812 | 1 | 794.2856, 804.2702, 674.2426, 176.0702 | alatusinine | TH | alkaloids |
| 42 | 25.295 | C_36_ H_43_ N O_18_ | M+H | 778.2544 | 0.51 | 750.2595, 351.1215, 222.0754, 720.2495 | wilfornine E or wilfordinine G | TH | alkaloids |
| 43* | 25.413 | C_26_ H_28_ O_10_ | M+H | 501.1749 | -1.66 | 523.1578（+Na）, 355.1173, 299.0547, 129.0556 | baohuoside II / ikarisoside A | EB | flavonoids |
| 44 | 25.887 | C_38_ H_47_ N O_19_ | M+H | 822.2821 | 0.35 | 804.2682, 762.2644, 744.2463, 250.1765 | wilfordinine B | TH | alkaloids |
| 45* | 26.783 | C_33_ H_40_ O_15_ | M+H | 677.2437 | -1.34 | 369.1339, 331.1004, 313.0706 | sagittatoside A | EB | flavonoids |
| 46 | 27.324 | C_20_ H_30_ O_3_ | M+H | 319.2259 | -1.98 | 255.2101, 131.0847 | tripterifordin | TH | terpenoids |
| 47* | 27.341 | C_32_ H_38_ O_14_ | M+H | 647.2327 | -2.01 | 369.1331, 313.0701, 301.0889, 515.1899 | sagittatoside B | EB | flavonoids |
| 48* | 27.493 | C_33_ H_40_ O_14_ | M+H | 661.2479 | -1.46 | 369.1325, 515.1901, 313.0702 | 2"-O-rhamnosylicariside II | EB | flavonoids |
| 49 | 28.14 | C_38_ H_47_ N O_18_ | M+K | 844.2418 | -1.56 | 788.2748, 746.2651, 704.2523, 686.2434, 206.0807 | wilfordinine E | TH | alkaloids |
| 50* | 28.44 | C_21_ H_20_ O_6_ | M+H | 369.1334 | -0.32 | 313.0704, 391.1164（+Na） | icaritin | EB | flavonoids |
| 51* | 28.457 | C_27_ H_30_ O_10_ | M+H | 515.1904 | -0.88 | 369.1324, 313.0704, 214.9176 | baohuoside I /Icarisid | EB | flavonoids |
| 52* | 28.508 | C_41_ H_47_ N O_20_ | M+H | 874.2759 | -0.38 | 846.2810, 828.2699, 786.2564, 674.2425, 194.0806 | wilfortrine | TH | alkaloids |
| 53 | 29.032 | C_38_ H_47_ N O_18_ | M+K | 844.2418 | -1.56 | 788.2748, 746.2651, 704.2523, 686.2431, 206.0806 | peritassine A | TH | alkaloids |
| 54 | 29.827 | C_39_ H_45_ N O_18_ | M+H | 816.2694 | -1.27 | 206.0802, 798.2607, 262.1056, 685.4323 | 1-desacetylwilforgine | TH | alkaloids |
| 55* | 30.148 | C_43_ H_49_ N O_19_ | M+H | 884.2973 | -0.4 | 856.3013, 866.2849, 838.2922, 674.2442, 194.0815 | wilfordine | TH | alkaloids |
| 56 | 30.452 | C_43_ H_49_ N O_19_ | M+H | 884.2962 | -1.25 | 840.2661, 762.2596, 718.2321, 685.4319 | wilfornine B | TH | alkaloids |
| 57 | 30.858 | C_43_ H_49_ N O_21_ | M+H | 916.2867 | -0.88 | 804.2705, 760.2442, 894.2424, 786.2587 | wilfornine D | TH | alkaloids |
| 58* | 31.044 | C_41_ H_47_ N O_19_ | M+Na | 880.2633 | -0.17 | 840.2692, 816.2701, 798.2592, 746.2642, 686.2427, 206。0805 | wilforgine | TH | alkaloids |
| 59 | 31.171 | C_20_H_24_O_3_ | M+H | 313.1813 | 3.05 | 225.1244 | triptophenolide | TH | terpenoids |
| 60* | 31.298 | C_38_ H_47_ N O_18_ | M+H | 806.2867 | 0 | 788.2750, 746.2644, 728.2536, 686.2427, 206.0806, 828.2682（+Na）, 844.2422(+K) | euonine | TH | alkaloids |
| 61 | 31.474 | C_36_H_43_NO_17_ | M+H | 762.2591 | -2.36 | 383.1525, 206.0811, 727.3488, 744.2481 | evonine | TH | alkaloids |
| 62 | 31.657 | C_41_H_47_NO_17_ | M+H | 826.2924 | 0.23 | 762.2613, 206.0799 | wilfornine F | TH | alkaloids |
| 63* | 32.16 | C_45_ H_51_ N O_20_ | M+Na | 948.2887 | -1.13 | 804.2696, 898.3138, 786.2599, 744.2476, 204.0646 | wilfornine A | TH | alkaloids |
| 64* | 32.651 | C_43_ H_49_ N O_18_ | M+H | 868.3019 | -0.7 | 850.2898, 826.2895, 808.2800, 746.2636, 206.0801 | wilforine | TH | alkaloids |
| 65 | 34.713 | C_45_ H_55_ N O_21_ | M+H | 946.3316 | -2.03 | 685.432, 351.13237, 214.9163 | triptonine A | TH | alkaloids |
| 66* | 35.889 | C_29_H_36_O_6_ | M+H | 481.2588 | -0.39 | 231.0654 | demethylzeylasteral | TH | terpenoids |
| 67* | 39 | C_29_H_38_O_4_ | M+H | 451.2866 | 3.97 | 201.0904 | celastrol | TH | terpenoids |

Note: TH means Tripterygium hypoglaucum (Lévl.) Hutch.; EB means Epimedium brevicornu Maxim.; CC means Cuscuta chinensis Lam.; LB means Lycium barbarum L.；* Compared with a reference standard.

Table S2. The predicted ADME behaviors of the 67 ingredients in KC.

| Peak No. | Name | Formula | CAS | OB in TCMSP (%) | Caco-2 in TCMSP (%) | DL in TCMSP (%) | Gl absorption in SwissADME | DL in SwissADME | Bioavailability Score in SwissADME (%) |
| --- | --- | --- | --- | --- | --- | --- | --- | --- | --- |
| 1 | arginine | C_6_H_14_N_4_O_2_ | 74-79-3 | 47.64 | -0.49 | 0.03 | Low | 3"Yes" and 2"No" | 0.55 |
| **2** | **proline** | C_5_H_9_NO_2_ | 37159-97-0 | 77.57 | 0.22 | 0.01 | High | 3"Yes" and 2"No" | 0.55 |
| **3** | **valine** | C_5_H_11_NO_2_ | 7004-03-7 | 53.33 | 0.04 | 0.01 | High | 3"Yes" and 2"No" | 0.55 |
| **4** | **betaine** | C_5_H_11_NO_2_ | 107-43-7 | 40.92 | -0.77 | 0.01 | Low | 3"Yes" and 2"No" | 0.55 |
| **5** | **epigallocatechin** | C_15_H_14_O_7_ | 970-74-1 | 24.18 | -0.22 | 0.27 | High | 4"Yes" and 1"No" | 0.55 |
| 6 | lycibarbarspermidine E | C_37_H_54_N_3_O_16_ | NA | NA | NA | NA | Low | 5"No" | 0.17 |
| 7 | lycibarbarspermidine L or K | C_37_ H_55_ N_3_ O_16_ | NA | NA | NA | NA | Low | 5"No" | 0.17 |
| 8 | procyanidin B-3 | C_30_H_26_O_12_ | 23567-23-9 | 3.01 | -1.04 | 0.66 | Low | 5"Yes" | 0.17 |
| 9 | lycibarbarspermidine M | C_37_H_55_N_3_O_16_ | NA | NA | NA | NA | Low | 5"No" | 0.17 |
| 10 | chlorogenic acid | C_16_H_18_O_9_ | 327-97-9 | 13.61 | -1.33 | 0.31 | Low | 1"Yes" and 4"No" | 0.11 |
| **11** | **catechin** | C_15_H_14_O_6_ | 7295-85-4 | 54.83 | -0.03 | 0.24 | High | 5"Yes" | 0.55 |
| 12 | lycibarbarspermidines F | C_37_ H_54_ N_3_ O_16_ | NA | NA | NA | NA | Low | 5"No" | 0.17 |
| 13 | lycibarbarspermidines H or I or J | C_31_H_46_N_3_O_11_ | NA | NA | NA | NA | Low | 5"No" | 0.17 |
| 14 | lycibarbarspermidine A or B or C or D | C_31_H_44_N_3_O_11_ | NA | NA | NA | NA | Low | 5"No" | 0.17 |
| **15** | **magnoflorine** | C_20_H_23_NO_4_ | 2141-09-5 | NA | NA | NA | High | 5"Yes" | 0.55 |
| **16** | **L-epicatechin** | C_15_H_14_O_6_ | 490-46-0 | 49.68 | -0.03 | 0.24 | High | 5"Yes" | 0.55 |
| 17 | rutin | C_27_H_30_O_16_ | 153-18-4 | 3.20 | -1.93 | 0.68 | Low | 5"No" | 0.17 |
| **18** | **hyperoside** | C_21_H_20_O_12_ | 482-36-0 | 6.94 | -1.42 | 0.77 | Low | 5"No" | 0.17 |
| 19 | isoquercitrin | C_21_H_20_O_12_ | 21637-25-2 | 1.87 | -1.50 | 0.77 | Low | 5"No" | 0.17 |
| 20 | luteolin-7-O-glucoside | C_21_H_20_O_11_ | 5373-11-5 | 7.29 | -1.23 | 0.78 | Low | 1"Yes" and 4"No" | 0.17 |
| 21 | kaempferitrin | C_27_H_30_O_14_ | 482-38-2 | 7.97 | -1.98 | 0.79 | Low | 5"No" | 0.17 |
| 22 | astragalin | C_21_H_20_O_11_ | 480-10-4 | 2.77 | -1.36 | 0.74 | Low | 1"Yes" and 4"No" | 0.17 |
| 23 | quercitrin | C_21_H_20_O_11_ | 522-12-3 | 2.61 | -1.12 | 0.74 | Low | 1"Yes" and 4"No" | 0.17 |
| **24** | **baohuosu** | C_22_H_22_O_7_ | 119730-90-4 | NA | NA | NA | High | 5"Yes" | 0.55 |
| **25** | **daidzein** | C_15_H_10_O_4_ | 486-66-8 | 19.44 | 0.59 | 0.19 | High | 5"Yes" | 0.55 |
| **26** | **epimedoside A** | C_32_H_38_O_15_ | 39012-04-9 | NA | NA | NA | Low | 5"No" | 0.17 |
| **27** | **triptolide** | C_20_H_24_O_6_ | 38748-32-2 | 51.29 | 0.25 | 0.68 | High | 5"Yes" | 0.55 |
| **28** | **quercetin** | C_15_H_10_O_7_ | 117-39-5 | 46.43 | 0.05 | 0.28 | High | 5"Yes" | 0.55 |
| **29** | **luteolin** | C_15_H_10_O_6_ | 491-70-3 | 36.16 | 0.19 | 0.25 | High | 5"Yes" | 0.55 |
| 30 | epimedin A1 | C_39_H_50_O_20_ | 140147-77-9 | NA | NA | NA | Low | 5"No" | 0.17 |
| **31** | **epimedin A** | C_39_H_50_O_20_ | 110623-72-8 | 6.06 | -2.64 | 0.32 | Low | 5"No" | 0.17 |
| **32** | **epimedin B** | C_38_H_48_O_19_ | 110623-73-9 | 8.65 | -2.89 | 0.34 | Low | 5"No" | 0.17 |
| **33** | **epimedin C** | C_39_H_50_O_19_ | 110642-44-9 | 6.06 | -2.77 | 0.32 | Low | 5"No" | 0.17 |
| **34** | **icariin** | C_33_H_40_O_15_ | 489-32-7 | 41.58 | -1.82 | 0.61 | Low | 5"No" | 0.17 |
| **35** | **kaempferol** | C_15_H_10_O_6_ | 520-18-3 | 41.88 | 0.26 | 0.24 | High | 5"Yes" | 0.55 |
| 36 | icaritin-3-O-rhamnoside | C_27_ H_32_O_11_ | NA | NA | NA | NA | Low | 5"No" | 0.17 |
| **37** | **isorhamnetin** | C_16_H_12_O_7_ | 480-19-3 | 49.60 | 0.31 | 0.31 | High | 5"Yes" | 0.55 |
| 38 | tripfordine A/wilforidine | C_36_H_45_NO_18_ | NA | NA | NA | NA | Low | 5"No" | 0.17 |
| 39 | carbonly-2''-β-L-quinovosyl icariin | C_39_H_48_O_19_ | NA | NA | NA | NA | Low | 5"No" | 0.17 |
| 40 | ikarisoside F | C_31_H_36_O_14_ | 113558-14-8 | 1.95 | -1.83 | 0.67 | Low | 5"No" | 0.17 |
| 41 | alatusinine | C_38_H_47_NO_19_ | 262599-03-1 | NA | NA | NA | Low | 5"No" | 0.17 |
| 42 | wilfornine E/wilfordinine G | C_36_H_43_NO_18_ | NA | 17.74 | -1.57 | 0.22 | Low | 5"No" | 0.17 |
| **43** | **baohuoside II/ikarisoside A** | C_26_H_28_O_10_ | 55395-07-8 | 4.75 | -0.70 | 0.82 | Low | 5"No" | 0.17 |
| 44 | wilfordinine B | C_38_H_47_NO_19_ | NA | NA | NA | NA | Low | 5"No" | 0.17 |
| 45 | sagittatoside A | C_33_ H_40_O_15_ | 118525-35-2 | 8.50 | -1.56 | 0.57 | Low | 5"No" | 0.17 |
| **46** | **tripterifordin** | C_20_H_30_O_3_ | 139122-81-9 | 76.13 | 0.65 | 0.49 | High | 5"Yes" | 0.55 |
| **47** | **sagittatoside B** | C_32_H_38_O_14_ | 118525-36-3 | 5.58 | -1.18 | 0.64 | Low | 5"No" | 0.17 |
| **48** | **2"-O-rhamnosylicariside II2″-O-rhamnosylicariside II (rha-icariside)** | C_33_H_40_O_14_ | 135293-13-9 | NA | NA | NA | Low | 5"No" | 0.17 |
| 49 | wilfordinine E | C_38_H_47_NO_18_ | 262601-67-2 | NA | NA | NA | Low | 5"No" | 0.17 |
| **50** | **icaritin** | C_21_H_20_O_6_ | 38226-86-7 | 28.27 | 0.77 | 0.59 | High | 5"Yes" | 0.55 |
| **51** | **baohuoside I/icarisid** | C_27_H_30_O_10_ | 113558-15-9 | 3.70 | -0.55 | 0.84 | Low | 1"Yes" and 4"No" | 0.55 |
| **52** | **wilfortrine** | C_41_H_47_NO_20_ | 37239-48-8 | 43.57 | -1.51 | 0.15 | Low | 5"No" | 0.17 |
| **53** | **peritassine A** | C_38_H_47_NO_18_ | 150881-01-9 | NA | NA | NA | Low | 5"No" | 0.17 |
| 54 | 1-desacetylwilforgine | C_39_H_45_NO_18_ | NA | NA | NA | NA | Low | 5"No" | 0.17 |
| **55** | **wilfordine** | C_43_H_49_NO_19_ | 37239-51-3 | 7.50 | -1.23 | 0.14 | Low | 5"No" | 0.17 |
| 56 | wilfornine B | C_43_H_49_NO_19_ | 262599-04-2 | NA | NA | NA | Low | 5"No" | 0.17 |
| **57** | **wilfornine D** | C_43_H_49_NO_21_ | NA | 24.39 | -1.12 | 0.14 | Low | 5"No" | 0.17 |
| **58** | **wilforgine** | C_41_H_47_NO_19_ | 37239-47-7 | 7.51 | -0.82 | 0.15 | Low | 5"No" | 0.17 |
| **59** | **triptophenolide** | C_20_H_24_O_3_ | 74285-86-2 | 48.50 | 1.11 | 0.44 | High | 5"Yes" | 0.55 |
| **60** | **euonine** | C_38_H_47_NO_18_ | 41758-69-4 | NA | NA | NA | Low | 5"No" | 0.17 |
| 61 | evonine | C_36_H_43_NO_17_ | 33458-82-1 | NA | NA | NA | Low | 5"No" | 0.17 |
| 62 | wilfornine F | C_41_H_47_NO_17_ | 128397-42-2 | NA | NA | NA | Low | 5"No" | 0.17 |
| **63** | **wilfornine A** | C_45_H_51_NO_20_ | 345954-00-9 | NA | NA | NA | Low | 5"No" | 0.17 |
| **64** | **wilforine** | C_43_H_49_NO_18_ | 11088-09-8 | 7.51 | -0.82 | 0.15 | Low | 5"No" | 0.17 |
| 65 | triptonine A | C_45_H_55_NO_21_ | NA | NA | NA | NA | Low | 5"No" | 0.17 |
| 66 | demethylzeylasteral | C_29_H_36_O_6_ | 107316-88-1 | NA | NA | NA | Low | 3"Yes" and 2"No" | 0.56 |
| **67** | **celastrol** | C_29_H_38_O_4_ | 34157-83-0 | 17.84 | 0.46 | 0.78 | Low | 2"Yes" and 3"No" | 0.85 |

Note: The compounds in bold text were screened out as putative active ingredients.

Table S3. Target prediction of the putative active ingredients.

| Ingredient | Target | Origin |
| --- | --- | --- |
| proline | GABRA3 GABRB2 GABRG2 | SwissTargetPrediction |
| proline | GABRA2 GABRB2 GABRG2 | SwissTargetPrediction |
| proline | SLC6A1 | SwissTargetPrediction |
| proline | ACE | SwissTargetPrediction |
| proline | REN | SwissTargetPrediction |
| proline | GABRR1 | SwissTargetPrediction |
| proline | EGLN1 | SwissTargetPrediction |
| proline | ANPEP | SwissTargetPrediction |
| proline | GABBR2 GABBR1 | SwissTargetPrediction |
| proline | F2 | SwissTargetPrediction |
| proline | LTA4H | SwissTargetPrediction |
| proline | OAT | SwissTargetPrediction |
| proline | ADORA3 | SwissTargetPrediction |
| proline | ITGA2B ITGB3 | SwissTargetPrediction |
| proline | NR1H4 | SwissTargetPrediction |
| proline | GABBR1 | SwissTargetPrediction |
| proline | PLA2G2A | SwissTargetPrediction |
| proline | PLA2G5 | SwissTargetPrediction |
| proline | NOS1 | SwissTargetPrediction |
| proline | NOS2 | SwissTargetPrediction |
| proline | NOS3 | SwissTargetPrediction |
| proline | MME | SwissTargetPrediction |
| proline | HSD17B3 | SwissTargetPrediction |
| proline | DPP4 | SwissTargetPrediction |
| proline | XIAP | SwissTargetPrediction |
| proline | CTSK | SwissTargetPrediction |
| proline | KISS1R | SwissTargetPrediction |
| proline | LAP3 | SwissTargetPrediction |
| proline | HSD11B1 | SwissTargetPrediction |
| proline | SLC1A2 | SwissTargetPrediction |
| proline | HLA-A | SwissTargetPrediction |
| proline | ABAT | SwissTargetPrediction |
| proline | SLC6A12 | SwissTargetPrediction |
| proline | ACE2 | SwissTargetPrediction |
| proline | PTGS2 | SwissTargetPrediction |
| proline | CPN1 | SwissTargetPrediction |
| proline | SLC5A1 | SwissTargetPrediction |
| proline | CAPN1 | SwissTargetPrediction |
| proline | CTSB | SwissTargetPrediction |
| proline | CHRNA4 CHRNB2 | SwissTargetPrediction |
| proline | CHRNA3 CHRNB4 | SwissTargetPrediction |
| proline | BBOX1 | SwissTargetPrediction |
| proline | EPHX2 | SwissTargetPrediction |
| proline | CNR1 | SwissTargetPrediction |
| proline | GABRA1 GABRB2 GABRG2 | SwissTargetPrediction |
| proline | SLC6A11 | SwissTargetPrediction |
| proline | SLC6A13 | SwissTargetPrediction |
| proline | DNPEP | SwissTargetPrediction |
| proline | PRKCA | SwissTargetPrediction |
| proline | CA2 | SwissTargetPrediction |
| proline | CA1 | SwissTargetPrediction |
| proline | CA12 | SwissTargetPrediction |
| proline | CA9 | SwissTargetPrediction |
| proline | DPP7 | SwissTargetPrediction |
| proline | SIRT2 | SwissTargetPrediction |
| proline | FAP | SwissTargetPrediction |
| proline | NAAA | SwissTargetPrediction |
| proline | GFPT1 | SwissTargetPrediction |
| proline | SLC5A7 | SwissTargetPrediction |
| proline | PGC | SwissTargetPrediction |
| proline | DLG4 | SwissTargetPrediction |
| proline | CTSH | SwissTargetPrediction |
| proline | GRM8 | SwissTargetPrediction |
| proline | CTSL | SwissTargetPrediction |
| proline | PLA2G4A | SwissTargetPrediction |
| proline | GRIK1 | SwissTargetPrediction |
| proline | GRIA1 | SwissTargetPrediction |
| proline | GRIK5 | SwissTargetPrediction |
| proline | GRIA4 | SwissTargetPrediction |
| proline | GRIK2 | SwissTargetPrediction |
| proline | GRIK3 | SwissTargetPrediction |
| proline | BACE1 | SwissTargetPrediction |
| proline | FKBP1A | SwissTargetPrediction |
| proline | SLC1A1 | SwissTargetPrediction |
| proline | GRM4 | SwissTargetPrediction |
| proline | GRM5 | SwissTargetPrediction |
| proline | GRM7 | SwissTargetPrediction |
| proline | PLA2G1B | SwissTargetPrediction |
| proline | SLC18A3 | SwissTargetPrediction |
| proline | METAP1 | SwissTargetPrediction |
| proline | SRD5A2 | SwissTargetPrediction |
| proline | CNR2 | SwissTargetPrediction |
| proline | DDAH1 | SwissTargetPrediction |
| proline | CTSD | SwissTargetPrediction |
| proline | XPNPEP1 | SwissTargetPrediction |
| proline | XPNPEP2 | SwissTargetPrediction |
| proline | PRKCE | SwissTargetPrediction |
| proline | PABPC1 | SwissTargetPrediction |
| proline | NTSR1 | SwissTargetPrediction |
| proline | FPR2 | SwissTargetPrediction |
| proline | ENPEP | SwissTargetPrediction |
| proline | GAA | SwissTargetPrediction |
| proline | SI | SwissTargetPrediction |
| proline | GSTK1 | SwissTargetPrediction |
| proline | CFB | SwissTargetPrediction |
| proline | GNPAT | SwissTargetPrediction |
| proline | AR | SwissTargetPrediction |
| proline | LSS | SwissTargetPrediction |
| proline | CHRNA7 | SwissTargetPrediction |
| proline | GANC | SwissTargetPrediction |
| proline | GABRA1 | SwissTargetPrediction |
| proline | PPIA | SwissTargetPrediction |
| proline | BCHE | SwissTargetPrediction |
| proline | ADH1B | SwissTargetPrediction |
| proline | ADH1C | SwissTargetPrediction |
| proline | LYZ | SwissTargetPrediction |
| proline | DAO | SwissTargetPrediction |
| proline | GATM | SwissTargetPrediction |
| proline | P4HA1 | SwissTargetPrediction |
| proline | PRSS3 | SwissTargetPrediction |
| proline | ADH1A | SwissTargetPrediction |
| valine | GABBR2 GABBR1 | SwissTargetPrediction |
| valine | SLC6A1 | SwissTargetPrediction |
| valine | GABRA3 GABRB2 GABRG2 | SwissTargetPrediction |
| valine | GABRA2 GABRB2 GABRG2 | SwissTargetPrediction |
| valine | GABRR1 | SwissTargetPrediction |
| valine | SLC6A11 | SwissTargetPrediction |
| valine | SLC6A13 | SwissTargetPrediction |
| valine | OAT | SwissTargetPrediction |
| valine | GABBR1 | SwissTargetPrediction |
| valine | HDAC3 | SwissTargetPrediction |
| valine | CACNA2D1 | SwissTargetPrediction |
| valine | ADORA3 | SwissTargetPrediction |
| valine | GRM4 | SwissTargetPrediction |
| valine | GRM5 | SwissTargetPrediction |
| valine | GRM8 | SwissTargetPrediction |
| valine | GRM7 | SwissTargetPrediction |
| valine | RNPEP | SwissTargetPrediction |
| valine | LTA4H | SwissTargetPrediction |
| valine | GABRA1 GABRB2 GABRG2 | SwissTargetPrediction |
| valine | SI | SwissTargetPrediction |
| valine | SLC6A12 | SwissTargetPrediction |
| valine | GRIK1 | SwissTargetPrediction |
| valine | GRIA1 | SwissTargetPrediction |
| valine | GRIK5 | SwissTargetPrediction |
| valine | GRIA4 | SwissTargetPrediction |
| valine | GRIK2 | SwissTargetPrediction |
| valine | GRIK3 | SwissTargetPrediction |
| valine | KMO | SwissTargetPrediction |
| valine | TH | SwissTargetPrediction |
| valine | GRIA2 | SwissTargetPrediction |
| valine | GBA | SwissTargetPrediction |
| valine | PYGL | SwissTargetPrediction |
| valine | GFPT1 | SwissTargetPrediction |
| valine | SLC6A3 | SwissTargetPrediction |
| valine | KYNU | SwissTargetPrediction |
| valine | EGLN1 | SwissTargetPrediction |
| valine | SLC1A1 | SwissTargetPrediction |
| valine | GRM1 | SwissTargetPrediction |
| valine | SLC22A6 | SwissTargetPrediction |
| valine | CA2 | SwissTargetPrediction |
| valine | CA1 | SwissTargetPrediction |
| valine | CA12 | SwissTargetPrediction |
| valine | CA9 | SwissTargetPrediction |
| valine | GRM2 | SwissTargetPrediction |
| valine | BBOX1 | SwissTargetPrediction |
| valine | CDC45 | SwissTargetPrediction |
| valine | PTPRC | SwissTargetPrediction |
| valine | CDC25A | SwissTargetPrediction |
| valine | CPN1 | SwissTargetPrediction |
| valine | MAN1B1 | SwissTargetPrediction |
| valine | GRM3 | SwissTargetPrediction |
| valine | GRM6 | SwissTargetPrediction |
| valine | FYN | SwissTargetPrediction |
| valine | EGFR | SwissTargetPrediction |
| valine | LCK | SwissTargetPrediction |
| valine | AGL | SwissTargetPrediction |
| valine | MGAM | SwissTargetPrediction |
| valine | GLA | SwissTargetPrediction |
| valine | FABP4 | SwissTargetPrediction |
| valine | PPARA | SwissTargetPrediction |
| valine | CPT2 | SwissTargetPrediction |
| valine | FABP3 | SwissTargetPrediction |
| valine | FABP5 | SwissTargetPrediction |
| valine | PPARD | SwissTargetPrediction |
| valine | HSD11B1 | SwissTargetPrediction |
| valine | FFAR1 | SwissTargetPrediction |
| valine | FABP2 | SwissTargetPrediction |
| valine | ABAT | SwissTargetPrediction |
| valine | PEPD | SwissTargetPrediction |
| valine | ENPEP | SwissTargetPrediction |
| valine | UGCG | SwissTargetPrediction |
| valine | ACE | SwissTargetPrediction |
| valine | GANAB | SwissTargetPrediction |
| valine | GAA | SwissTargetPrediction |
| valine | KDM2A | SwissTargetPrediction |
| valine | KDM6B | SwissTargetPrediction |
| valine | PHF8 | SwissTargetPrediction |
| valine | KDM5C | SwissTargetPrediction |
| valine | SHBG | SwissTargetPrediction |
| valine | FUCA1 | SwissTargetPrediction |
| valine | HMGCR | SwissTargetPrediction |
| valine | GBA2 | SwissTargetPrediction |
| valine | KDM4C | SwissTargetPrediction |
| valine | GLB1 | SwissTargetPrediction |
| valine | SLC1A2 | SwissTargetPrediction |
| valine | AR | SwissTargetPrediction |
| valine | ANPEP | SwissTargetPrediction |
| valine | VDR | SwissTargetPrediction |
| valine | NR1H4 | SwissTargetPrediction |
| valine | GPBAR1 | SwissTargetPrediction |
| valine | AKR1B10 | SwissTargetPrediction |
| valine | POLB | SwissTargetPrediction |
| valine | CPB2 | SwissTargetPrediction |
| valine | LAP3 | SwissTargetPrediction |
| valine | PLG | SwissTargetPrediction |
| valine | ODC1 | SwissTargetPrediction |
| valine | CPA1 | SwissTargetPrediction |
| valine | KDM4E | SwissTargetPrediction |
| valine | PTGS1 | SwissTargetPrediction |
| valine | PTGS2 | SwissTargetPrediction |
| valine | AKR1B1 | SwissTargetPrediction |
| valine | GABRA1 | SwissTargetPrediction |
| valine | BCHE | SwissTargetPrediction |
| valine | ADH1C | SwissTargetPrediction |
| valine | GOT2 | SwissTargetPrediction |
| valine | GATM | SwissTargetPrediction |
| valine | ME2 | SwissTargetPrediction |
| valine | GOT1 | SwissTargetPrediction |
| valine | CTSD | SwissTargetPrediction |
| valine | OTC | SwissTargetPrediction |
| valine | CTH | SwissTargetPrediction |
| valine | MAOB | SwissTargetPrediction |
| betaine | SLC5A7 | SwissTargetPrediction |
| betaine | BBOX1 | SwissTargetPrediction |
| betaine | ADSS | SwissTargetPrediction |
| betaine | KDM3A | SwissTargetPrediction |
| betaine | KDM6B | SwissTargetPrediction |
| betaine | CA2 | SwissTargetPrediction |
| betaine | KDM5C | SwissTargetPrediction |
| betaine | CA1 | SwissTargetPrediction |
| betaine | EGLN2 | SwissTargetPrediction |
| betaine | CA12 | SwissTargetPrediction |
| betaine | CA9 | SwissTargetPrediction |
| betaine | EGLN1 | SwissTargetPrediction |
| betaine | CHRM4 | SwissTargetPrediction |
| betaine | CHRNA4 CHRNB2 | SwissTargetPrediction |
| betaine | CHRM5 | SwissTargetPrediction |
| betaine | CHRM2 | SwissTargetPrediction |
| betaine | CHRM1 | SwissTargetPrediction |
| betaine | CHRM3 | SwissTargetPrediction |
| betaine | CHRNA7 | SwissTargetPrediction |
| betaine | PTGS1 | TCMSP |
| betaine | ABAT | TCMSP |
| betaine | GOT1 | TCMSP |
| betaine | AKR1B1 | TCMSP |
| betaine | GABRA1 | TCMSP |
| betaine | CTSD | TCMSP |
| betaine | BCHE | TCMSP |
| betaine | ADH1B | TCMSP |
| betaine | ADH1C | TCMSP |
| betaine | GOT2 | TCMSP |
| betaine | GATM | TCMSP |
| betaine | TPI1 | TCMSP |
| betaine | ME2 | TCMSP |
| betaine | PTGS2 | TCMSP |
| betaine | GRIA1 | TCMSP |
| betaine | CDC25B | TCMSP |
| betaine | MAOB | TCMSP |
| epigallocatechin | PTGS1 | TCMSP |
| epigallocatechin | ESR1 | TCMSP |
| epigallocatechin | PTGS2 | TCMSP |
| epigallocatechin | NCOA2 | TCMSP |
| epigallocatechin | CXCL8 | TCMSP |
| catechin | PTGS1 | TCMSP |
| catechin | ESR1 | TCMSP |
| catechin | PTGS2 | TCMSP |
| catechin | NCOA2 | TCMSP |
| catechin | RXRA | TCMSP |
| catechin | CAT | TCMSP |
| catechin | HAS2 | TCMSP |
| magnoflorine | DRD2 | SwissTargetPrediction |
| magnoflorine | CHRNA4 CHRNB2 | SwissTargetPrediction |
| magnoflorine | DRD3 | SwissTargetPrediction |
| magnoflorine | DRD1 | SwissTargetPrediction |
| magnoflorine | HTR1A | SwissTargetPrediction |
| magnoflorine | HTR6 | SwissTargetPrediction |
| magnoflorine | HTR7 | SwissTargetPrediction |
| magnoflorine | PTPRCAP | SwissTargetPrediction |
| magnoflorine | HTR2B | SwissTargetPrediction |
| magnoflorine | ADRA1D | SwissTargetPrediction |
| magnoflorine | HTR2A | SwissTargetPrediction |
| magnoflorine | TH | SwissTargetPrediction |
| magnoflorine | DRD4 | SwissTargetPrediction |
| magnoflorine | HTR5A | SwissTargetPrediction |
| magnoflorine | ADRA1A | SwissTargetPrediction |
| magnoflorine | HTR1D | SwissTargetPrediction |
| magnoflorine | ADRB1 | SwissTargetPrediction |
| magnoflorine | SLC6A3 | SwissTargetPrediction |
| magnoflorine | OPRM1 | SwissTargetPrediction |
| magnoflorine | DRD5 | SwissTargetPrediction |
| magnoflorine | HTR2C | SwissTargetPrediction |
| magnoflorine | KCNH2 | SwissTargetPrediction |
| magnoflorine | ADRA1B | SwissTargetPrediction |
| magnoflorine | TSPO | SwissTargetPrediction |
| magnoflorine | SLC6A4 | SwissTargetPrediction |
| magnoflorine | ADRA2A | SwissTargetPrediction |
| magnoflorine | ADRA2C | SwissTargetPrediction |
| magnoflorine | ADRA2B | SwissTargetPrediction |
| magnoflorine | HTR1B | SwissTargetPrediction |
| magnoflorine | HRH2 | SwissTargetPrediction |
| magnoflorine | PPARG | SwissTargetPrediction |
| magnoflorine | ADRB2 | SwissTargetPrediction |
| magnoflorine | CDK5R1 CDK5 | SwissTargetPrediction |
| magnoflorine | GSK3B | SwissTargetPrediction |
| magnoflorine | CDK2 CCNA1 CCNA2 | SwissTargetPrediction |
| magnoflorine | NR3C2 | SwissTargetPrediction |
| magnoflorine | EGFR | SwissTargetPrediction |
| magnoflorine | HPGD | SwissTargetPrediction |
| magnoflorine | AR | SwissTargetPrediction |
| magnoflorine | CYP19A1 | SwissTargetPrediction |
| magnoflorine | MGLL | SwissTargetPrediction |
| magnoflorine | TRPC6 | SwissTargetPrediction |
| magnoflorine | TRPC3 | SwissTargetPrediction |
| magnoflorine | JAK3 | SwissTargetPrediction |
| magnoflorine | PTPN1 | SwissTargetPrediction |
| magnoflorine | SIGMAR1 | SwissTargetPrediction |
| magnoflorine | PIM1 | SwissTargetPrediction |
| magnoflorine | DYRK1A | SwissTargetPrediction |
| magnoflorine | STAT3 | SwissTargetPrediction |
| magnoflorine | CLK1 | SwissTargetPrediction |
| magnoflorine | DYRK2 | SwissTargetPrediction |
| magnoflorine | PRKCG | SwissTargetPrediction |
| magnoflorine | GABRA1 | SwissTargetPrediction |
| magnoflorine | EPHX2 | SwissTargetPrediction |
| magnoflorine | VCAM1 | SwissTargetPrediction |
| magnoflorine | GABRA5 | SwissTargetPrediction |
| magnoflorine | MAP2K1 | SwissTargetPrediction |
| magnoflorine | ADAMTS5 | SwissTargetPrediction |
| magnoflorine | RPS6KB1 | SwissTargetPrediction |
| magnoflorine | KAT2B | SwissTargetPrediction |
| magnoflorine | SIRT2 | SwissTargetPrediction |
| magnoflorine | FLT3 | SwissTargetPrediction |
| magnoflorine | CLK3 | SwissTargetPrediction |
| magnoflorine | PTGES | SwissTargetPrediction |
| magnoflorine | CSF1R | SwissTargetPrediction |
| magnoflorine | MAPKAPK2 | SwissTargetPrediction |
| magnoflorine | KDR | SwissTargetPrediction |
| magnoflorine | CDK1 | SwissTargetPrediction |
| magnoflorine | IRAK4 | SwissTargetPrediction |
| magnoflorine | ALOX15 | SwissTargetPrediction |
| magnoflorine | ALOX12 | SwissTargetPrediction |
| magnoflorine | PIK3CB | SwissTargetPrediction |
| magnoflorine | PIK3CG | SwissTargetPrediction |
| magnoflorine | PIK3CA | SwissTargetPrediction |
| magnoflorine | HSD17B3 | SwissTargetPrediction |
| magnoflorine | PDGFRB | SwissTargetPrediction |
| magnoflorine | FLT4 | SwissTargetPrediction |
| magnoflorine | SRC | SwissTargetPrediction |
| magnoflorine | ICAM1 | SwissTargetPrediction |
| magnoflorine | SELE | SwissTargetPrediction |
| magnoflorine | PPP5C | SwissTargetPrediction |
| magnoflorine | CCNB3 CDK1 CCNB1 CCNB2 | SwissTargetPrediction |
| magnoflorine | DBF4 CDC7 | SwissTargetPrediction |
| magnoflorine | AURKA | SwissTargetPrediction |
| magnoflorine | TRPV1 | SwissTargetPrediction |
| magnoflorine | TRPM8 | SwissTargetPrediction |
| magnoflorine | PGR | SwissTargetPrediction |
| magnoflorine | MMP3 | SwissTargetPrediction |
| magnoflorine | MMP2 | SwissTargetPrediction |
| magnoflorine | ADORA2A | SwissTargetPrediction |
| magnoflorine | MAPK1 | SwissTargetPrediction |
| magnoflorine | PDE10A | SwissTargetPrediction |
| magnoflorine | CXCR3 | SwissTargetPrediction |
| magnoflorine | CDK2 | SwissTargetPrediction |
| magnoflorine | MMP9 | SwissTargetPrediction |
| magnoflorine | PDGFRA PDGFRB | SwissTargetPrediction |
| magnoflorine | AURKB | SwissTargetPrediction |
| magnoflorine | ROCK2 | SwissTargetPrediction |
| magnoflorine | FGFR1 | SwissTargetPrediction |
| magnoflorine | AKT1 | SwissTargetPrediction |
| l-epicatechin | PTGS1 | TCMSP |
| l-epicatechin | ESR1 | TCMSP |
| l-epicatechin | PTGS2 | TCMSP |
| l-epicatechin | RXRA | TCMSP |
| l-epicatechin | NCOA2 | TCMSP |
| l-epicatechin | AKT1 | TCMSP |
| l-epicatechin | PLAU | TCMSP |
| l-epicatechin | TNFSF15 | TCMSP |
| l-epicatechin | JUN | TCMSP |
| l-epicatechin | IL6 | TCMSP |
| l-epicatechin | CASP3 | TCMSP |
| l-epicatechin | POR | TCMSP |
| l-epicatechin | COMT | TCMSP |
| l-epicatechin | CREB1 | TCMSP |
| l-epicatechin | CCL2 | TCMSP |
| l-epicatechin | DUOX2 | TCMSP |
| l-epicatechin | IL2 | TCMSP |
| l-epicatechin | PLAT | TCMSP |
| l-epicatechin | IL1A | TCMSP |
| l-epicatechin | GCLC | TCMSP |
| l-epicatechin | GSS | TCMSP |
| l-epicatechin | ABCG2 | TCMSP |
| l-epicatechin | ACE | TCMSP |
| l-epicatechin | HAS2 | TCMSP |
| l-epicatechin | GRIA2 | TCMSP |
| l-epicatechin | CRTC2 | TCMSP |
| hyperoside | NOX4 | SwissTargetPrediction |
| hyperoside | ADRA2C | SwissTargetPrediction |
| hyperoside | AKR1B1 | SwissTargetPrediction |
| hyperoside | CA2 | SwissTargetPrediction |
| hyperoside | CA7 | SwissTargetPrediction |
| hyperoside | CA12 | SwissTargetPrediction |
| hyperoside | CA4 | SwissTargetPrediction |
| hyperoside | ACHE | SwissTargetPrediction |
| hyperoside | NQO2 | SwissTargetPrediction |
| hyperoside | RPS6KA3 | SwissTargetPrediction |
| hyperoside | NMUR2 | SwissTargetPrediction |
| hyperoside | ADRA2A | SwissTargetPrediction |
| hyperoside | PTGS2 | SwissTargetPrediction |
| hyperoside | CD38 | SwissTargetPrediction |
| hyperoside | PDE5A | SwissTargetPrediction |
| hyperoside | TNF | SwissTargetPrediction |
| hyperoside | IL2 | SwissTargetPrediction |
| hyperoside | ADORA1 | SwissTargetPrediction |
| hyperoside | XDH | SwissTargetPrediction |
| hyperoside | ALOX5 | SwissTargetPrediction |
| hyperoside | SLC29A1 | SwissTargetPrediction |
| hyperoside | TERT | SwissTargetPrediction |
| hyperoside | ADORA3 | SwissTargetPrediction |
| hyperoside | PLG | SwissTargetPrediction |
| hyperoside | KCNA3 | SwissTargetPrediction |
| hyperoside | ABCG2 | SwissTargetPrediction |
| hyperoside | SRC | SwissTargetPrediction |
| hyperoside | APP | SwissTargetPrediction |
| hyperoside | CYP1B1 | SwissTargetPrediction |
| hyperoside | MCL1 | SwissTargetPrediction |
| hyperoside | ALDH2 | SwissTargetPrediction |
| hyperoside | ITGAV ITGB3 | SwissTargetPrediction |
| hyperoside | ITGAV ITGB6 | SwissTargetPrediction |
| hyperoside | F10 | SwissTargetPrediction |
| hyperoside | KISS1R | SwissTargetPrediction |
| hyperoside | CA1 | SwissTargetPrediction |
| hyperoside | CA9 | SwissTargetPrediction |
| hyperoside | CA13 | SwissTargetPrediction |
| hyperoside | ITGA2B ITGB3 | SwissTargetPrediction |
| hyperoside | ITGB1 ITGA5 | SwissTargetPrediction |
| hyperoside | HSP90AB1 | SwissTargetPrediction |
| hyperoside | ADORA2A | SwissTargetPrediction |
| hyperoside | CHEK2 | SwissTargetPrediction |
| hyperoside | CHEK1 | SwissTargetPrediction |
| hyperoside | PRKCG | SwissTargetPrediction |
| hyperoside | PRKCD | SwissTargetPrediction |
| hyperoside | PRKCA | SwissTargetPrediction |
| hyperoside | PRKCB | SwissTargetPrediction |
| hyperoside | PRKCE | SwissTargetPrediction |
| hyperoside | PRKCH | SwissTargetPrediction |
| hyperoside | DNM2 | SwissTargetPrediction |
| hyperoside | MAPT | SwissTargetPrediction |
| hyperoside | KDM4E | SwissTargetPrediction |
| hyperoside | GPR35 | SwissTargetPrediction |
| hyperoside | AVPR2 | SwissTargetPrediction |
| hyperoside | TOP2A | SwissTargetPrediction |
| hyperoside | MAOA | SwissTargetPrediction |
| hyperoside | IGF1R | SwissTargetPrediction |
| hyperoside | FLT3 | SwissTargetPrediction |
| hyperoside | CYP19A1 | SwissTargetPrediction |
| hyperoside | INSR | SwissTargetPrediction |
| hyperoside | EGFR | SwissTargetPrediction |
| hyperoside | PIM1 | SwissTargetPrediction |
| hyperoside | AURKB | SwissTargetPrediction |
| hyperoside | DRD4 | SwissTargetPrediction |
| hyperoside | GLO1 | SwissTargetPrediction |
| hyperoside | MYLK | SwissTargetPrediction |
| hyperoside | MPO | SwissTargetPrediction |
| hyperoside | PIK3R1 | SwissTargetPrediction |
| hyperoside | DAPK1 | SwissTargetPrediction |
| hyperoside | PYGL | SwissTargetPrediction |
| hyperoside | SYK | SwissTargetPrediction |
| hyperoside | GSK3B | SwissTargetPrediction |
| hyperoside | PTK2 | SwissTargetPrediction |
| hyperoside | HSD17B2 | SwissTargetPrediction |
| hyperoside | KDR | SwissTargetPrediction |
| hyperoside | MMP13 | SwissTargetPrediction |
| hyperoside | MMP3 | SwissTargetPrediction |
| hyperoside | CA3 | SwissTargetPrediction |
| hyperoside | ALOX15 | SwissTargetPrediction |
| hyperoside | PLK1 | SwissTargetPrediction |
| hyperoside | CA6 | SwissTargetPrediction |
| hyperoside | CDK1 | SwissTargetPrediction |
| hyperoside | MMP9 | SwissTargetPrediction |
| hyperoside | PIK3CG | SwissTargetPrediction |
| hyperoside | MMP2 | SwissTargetPrediction |
| hyperoside | PKN1 | SwissTargetPrediction |
| hyperoside | CA14 | SwissTargetPrediction |
| hyperoside | CSNK2A1 | SwissTargetPrediction |
| hyperoside | ALOX12 | SwissTargetPrediction |
| hyperoside | MET | SwissTargetPrediction |
| hyperoside | NEK2 | SwissTargetPrediction |
| hyperoside | CXCR1 | SwissTargetPrediction |
| hyperoside | CAMK2B | SwissTargetPrediction |
| hyperoside | ALK | SwissTargetPrediction |
| hyperoside | AKT1 | SwissTargetPrediction |
| hyperoside | NEK6 | SwissTargetPrediction |
| hyperoside | PLA2G1B | SwissTargetPrediction |
| hyperoside | CA5A | SwissTargetPrediction |
| hyperoside | AXL | SwissTargetPrediction |
| hyperoside | PRSS1 | SwissTargetPrediction |
| hyperoside | NOS2 | SwissTargetPrediction |
| hyperoside | PTGS1 | SwissTargetPrediction |
| hyperoside | NCOA2 | SwissTargetPrediction |
| baohuosu | AKT1 | SwissTargetPrediction |
| baohuosu | PDE5A | SwissTargetPrediction |
| baohuosu | ABCB1 | SwissTargetPrediction |
| baohuosu | ABCG2 | SwissTargetPrediction |
| baohuosu | CYP19A1 | SwissTargetPrediction |
| baohuosu | PTPN1 | SwissTargetPrediction |
| baohuosu | PTGS1 | SwissTargetPrediction |
| baohuosu | AKR1B1 | SwissTargetPrediction |
| baohuosu | BACE1 | SwissTargetPrediction |
| baohuosu | BCHE | SwissTargetPrediction |
| baohuosu | ACHE | SwissTargetPrediction |
| baohuosu | ABCC1 | SwissTargetPrediction |
| baohuosu | CYP1B1 | SwissTargetPrediction |
| baohuosu | PPARG | SwissTargetPrediction |
| baohuosu | HSP90AB1 | SwissTargetPrediction |
| baohuosu | GSK3B | SwissTargetPrediction |
| baohuosu | PDK1 | SwissTargetPrediction |
| baohuosu | PLA2G2A | SwissTargetPrediction |
| baohuosu | APP | SwissTargetPrediction |
| baohuosu | HSP90AA1 | SwissTargetPrediction |
| baohuosu | BCL2L1 | SwissTargetPrediction |
| baohuosu | BCL2 | SwissTargetPrediction |
| baohuosu | MELK | SwissTargetPrediction |
| baohuosu | HSP90B1 | SwissTargetPrediction |
| baohuosu | PTGS2 | SwissTargetPrediction |
| baohuosu | CA2 | SwissTargetPrediction |
| baohuosu | PLG | SwissTargetPrediction |
| baohuosu | CDK5R1 CDK5 | SwissTargetPrediction |
| baohuosu | CA7 | SwissTargetPrediction |
| baohuosu | CA12 | SwissTargetPrediction |
| baohuosu | CTNNB1 | SwissTargetPrediction |
| baohuosu | OPRD1 | SwissTargetPrediction |
| baohuosu | ALOX15 | SwissTargetPrediction |
| baohuosu | CHEK2 | SwissTargetPrediction |
| baohuosu | CHEK1 | SwissTargetPrediction |
| baohuosu | SYK | SwissTargetPrediction |
| baohuosu | PARP1 | SwissTargetPrediction |
| baohuosu | TTR | SwissTargetPrediction |
| baohuosu | CD38 | SwissTargetPrediction |
| baohuosu | AKR1B10 | SwissTargetPrediction |
| baohuosu | TNKS2 | SwissTargetPrediction |
| baohuosu | TNKS | SwissTargetPrediction |
| baohuosu | TOP1 | SwissTargetPrediction |
| baohuosu | ARG1 | SwissTargetPrediction |
| baohuosu | CDK1 | SwissTargetPrediction |
| baohuosu | PIM1 | SwissTargetPrediction |
| baohuosu | AGTR1 | SwissTargetPrediction |
| baohuosu | XDH | SwissTargetPrediction |
| baohuosu | CNOT7 | SwissTargetPrediction |
| baohuosu | CA4 | SwissTargetPrediction |
| baohuosu | ADRB2 | SwissTargetPrediction |
| baohuosu | ADRB1 | SwissTargetPrediction |
| baohuosu | F10 | SwissTargetPrediction |
| baohuosu | PLAU | SwissTargetPrediction |
| baohuosu | CCNB3 CDK1 CCNB1 CCNB2 | SwissTargetPrediction |
| baohuosu | ESR2 | SwissTargetPrediction |
| baohuosu | ERBB2 | SwissTargetPrediction |
| baohuosu | GCGR | SwissTargetPrediction |
| baohuosu | CDK2 | SwissTargetPrediction |
| baohuosu | PRKCI | SwissTargetPrediction |
| baohuosu | CHUK | SwissTargetPrediction |
| baohuosu | KISS1R | SwissTargetPrediction |
| baohuosu | EGLN1 | SwissTargetPrediction |
| baohuosu | FASN | SwissTargetPrediction |
| baohuosu | RELA | SwissTargetPrediction |
| baohuosu | CDK6 | SwissTargetPrediction |
| baohuosu | HSD17B2 | SwissTargetPrediction |
| baohuosu | OPRK1 | SwissTargetPrediction |
| baohuosu | HSD17B1 | SwissTargetPrediction |
| baohuosu | KLK1 | SwissTargetPrediction |
| baohuosu | KLK2 | SwissTargetPrediction |
| baohuosu | MAOA | SwissTargetPrediction |
| baohuosu | ALOX12 | SwissTargetPrediction |
| baohuosu | NOX4 | SwissTargetPrediction |
| baohuosu | PDE10A | SwissTargetPrediction |
| baohuosu | CCR4 | SwissTargetPrediction |
| baohuosu | FBP1 | SwissTargetPrediction |
| baohuosu | DHODH | SwissTargetPrediction |
| baohuosu | DHFR | SwissTargetPrediction |
| baohuosu | TERT | SwissTargetPrediction |
| baohuosu | NOS2 | SwissTargetPrediction |
| baohuosu | CALCA | SwissTargetPrediction |
| baohuosu | GPR84 | SwissTargetPrediction |
| baohuosu | EGFR | SwissTargetPrediction |
| baohuosu | ACP1 | SwissTargetPrediction |
| baohuosu | KLKB1 | SwissTargetPrediction |
| baohuosu | CFTR | SwissTargetPrediction |
| baohuosu | RAF1 | SwissTargetPrediction |
| baohuosu | MAPT | SwissTargetPrediction |
| baohuosu | KDM4E | SwissTargetPrediction |
| baohuosu | TOP2A | SwissTargetPrediction |
| baohuosu | MYLK | SwissTargetPrediction |
| baohuosu | MPO | SwissTargetPrediction |
| baohuosu | PIK3R1 | SwissTargetPrediction |
| baohuosu | DAPK1 | SwissTargetPrediction |
| baohuosu | PYGL | SwissTargetPrediction |
| baohuosu | MMP3 | SwissTargetPrediction |
| baohuosu | CA3 | SwissTargetPrediction |
| baohuosu | PLK1 | SwissTargetPrediction |
| baohuosu | CSNK2A1 | SwissTargetPrediction |
| daidzein | ALDH2 | SwissTargetPrediction |
| daidzein | ESR1 | TCMSP |
| daidzein | CA7 | SwissTargetPrediction |
| daidzein | ESR2 | SwissTargetPrediction |
| daidzein | CA12 | SwissTargetPrediction |
| daidzein | CA4 | SwissTargetPrediction |
| daidzein | TBXAS1 | SwissTargetPrediction |
| daidzein | MAOA | SwissTargetPrediction |
| daidzein | EGFR | SwissTargetPrediction |
| daidzein | MGAM | SwissTargetPrediction |
| daidzein | HTR2A | SwissTargetPrediction |
| daidzein | HTR2C | SwissTargetPrediction |
| daidzein | ADORA1 | SwissTargetPrediction |
| daidzein | ADORA2A | SwissTargetPrediction |
| daidzein | HSD17B1 | SwissTargetPrediction |
| daidzein | ESRRA | SwissTargetPrediction |
| daidzein | ESRRB | SwissTargetPrediction |
| daidzein | ABCG2 | SwissTargetPrediction |
| daidzein | CYP19A1 | SwissTargetPrediction |
| daidzein | ALOX12 | SwissTargetPrediction |
| daidzein | TYR | SwissTargetPrediction |
| daidzein | MIF | SwissTargetPrediction |
| daidzein | XDH | SwissTargetPrediction |
| daidzein | PFKFB3 | SwissTargetPrediction |
| daidzein | IL2 | SwissTargetPrediction |
| daidzein | CBR1 | SwissTargetPrediction |
| daidzein | HSD17B2 | SwissTargetPrediction |
| daidzein | ABCB1 | SwissTargetPrediction |
| daidzein | PTPRS | SwissTargetPrediction |
| daidzein | ALOX15 | SwissTargetPrediction |
| daidzein | PTGS1 | TCMSP |
| daidzein | SLC6A2 | SwissTargetPrediction |
| daidzein | NOX4 | SwissTargetPrediction |
| daidzein | MAOB | SwissTargetPrediction |
| daidzein | PON1 | SwissTargetPrediction |
| daidzein | CA2 | SwissTargetPrediction |
| daidzein | CA1 | SwissTargetPrediction |
| daidzein | STS | SwissTargetPrediction |
| daidzein | ACHE | SwissTargetPrediction |
| daidzein | TLR9 | SwissTargetPrediction |
| daidzein | PPARA | SwissTargetPrediction |
| daidzein | ALOX5 | SwissTargetPrediction |
| daidzein | TNKS2 | SwissTargetPrediction |
| daidzein | TNKS | SwissTargetPrediction |
| daidzein | PTPN1 | SwissTargetPrediction |
| daidzein | CA9 | SwissTargetPrediction |
| daidzein | PLAT | SwissTargetPrediction |
| daidzein | F10 | SwissTargetPrediction |
| daidzein | PLAU | SwissTargetPrediction |
| daidzein | CA5B | SwissTargetPrediction |
| daidzein | PPARG | TCMSP |
| daidzein | MCL1 | SwissTargetPrediction |
| daidzein | CDK5R1 CDK5 | SwissTargetPrediction |
| daidzein | PARP1 | SwissTargetPrediction |
| daidzein | AKR1B1 | SwissTargetPrediction |
| daidzein | SNCA | SwissTargetPrediction |
| daidzein | IGFBP3 | SwissTargetPrediction |
| daidzein | HSD17B3 | SwissTargetPrediction |
| daidzein | CRHR1 | SwissTargetPrediction |
| daidzein | BAD | SwissTargetPrediction |
| daidzein | PTP4A3 | SwissTargetPrediction |
| daidzein | FLT3 | SwissTargetPrediction |
| daidzein | CCNB3 CDK1 CCNB1 CCNB2 | SwissTargetPrediction |
| daidzein | PTGS2 | TCMSP |
| daidzein | CDK6 | SwissTargetPrediction |
| daidzein | SYK | SwissTargetPrediction |
| daidzein | GSK3B | SwissTargetPrediction |
| daidzein | ABCC1 | SwissTargetPrediction |
| daidzein | TTR | SwissTargetPrediction |
| daidzein | CSNK2A1 | SwissTargetPrediction |
| daidzein | CFTR | SwissTargetPrediction |
| daidzein | CYP1B1 | SwissTargetPrediction |
| daidzein | AKR1B10 | SwissTargetPrediction |
| daidzein | HSP90AA1 | SwissTargetPrediction |
| daidzein | PIM1 | SwissTargetPrediction |
| daidzein | AURKB | SwissTargetPrediction |
| daidzein | PLA2G4A | SwissTargetPrediction |
| daidzein | CDC7 | SwissTargetPrediction |
| daidzein | CA3 | SwissTargetPrediction |
| daidzein | CA6 | SwissTargetPrediction |
| daidzein | CA14 | SwissTargetPrediction |
| daidzein | CA13 | SwissTargetPrediction |
| daidzein | CA5A | SwissTargetPrediction |
| daidzein | KLKB1 | SwissTargetPrediction |
| daidzein | FNTA FNTB | SwissTargetPrediction |
| daidzein | IGFBP6 | SwissTargetPrediction |
| daidzein | IGFBP4 | SwissTargetPrediction |
| daidzein | IGFBP5 | SwissTargetPrediction |
| daidzein | IGFBP2 | SwissTargetPrediction |
| daidzein | IGFBP1 | SwissTargetPrediction |
| daidzein | HSP90AB1 | SwissTargetPrediction |
| daidzein | RAF1 | SwissTargetPrediction |
| daidzein | NTRK1 | SwissTargetPrediction |
| daidzein | PIM2 | SwissTargetPrediction |
| daidzein | BRAF | SwissTargetPrediction |
| daidzein | PRMT1 | SwissTargetPrediction |
| daidzein | PRSS1 | TCMSP |
| daidzein | CXCR2 | SwissTargetPrediction |
| daidzein | DHODH | SwissTargetPrediction |
| daidzein | CES2 | SwissTargetPrediction |
| daidzein | RXRA | TCMSP |
| daidzein | ADRB2 | TCMSP |
| daidzein | MAPK14 | TCMSP |
| daidzein | CHEK1 | TCMSP |
| daidzein | RELA | TCMSP |
| daidzein | VEGFA | TCMSP |
| daidzein | FOS | TCMSP |
| daidzein | CDKN1A | TCMSP |
| daidzein | EIF6 | TCMSP |
| daidzein | BAX | TCMSP |
| daidzein | TNFSF15 | TCMSP |
| daidzein | JUN | TCMSP |
| daidzein | IL6 | TCMSP |
| daidzein | NOS2 | TCMSP |
| daidzein | CASP3 | TCMSP |
| daidzein | TP63 | TCMSP |
| daidzein | LDLR | TCMSP |
| daidzein | CAT | TCMSP |
| daidzein | IGF1R | TCMSP |
| daidzein | STAT1 | TCMSP |
| daidzein | CYP3A4 | TCMSP |
| daidzein | CAV1 | TCMSP |
| daidzein | ICAM1 | TCMSP |
| daidzein | MTTP | TCMSP |
| daidzein | APOB | TCMSP |
| daidzein | VCAM1 | TCMSP |
| daidzein | SOD2 | TCMSP |
| daidzein | NOS3 | TCMSP |
| daidzein | ECE1 | TCMSP |
| daidzein | GABBR1 | TCMSP |
| daidzein | IL4 | TCMSP |
| daidzein | CPT1A | TCMSP |
| daidzein | AHR | TCMSP |
| daidzein | RHOA | TCMSP |
| daidzein | MT2A | TCMSP |
| daidzein | FCER2 | TCMSP |
| daidzein | EP300 | TCMSP |
| daidzein | ATP5F1B | TCMSP |
| daidzein | ND6 | TCMSP |
| daidzein | HSD3B2 | TCMSP |
| daidzein | HSD3B1 | TCMSP |
| daidzein | TFF1 | TCMSP |
| daidzein | TRPM2 | TCMSP |
| daidzein | GH1 | TCMSP |
| daidzein | PPARGC1B | TCMSP |
| daidzein | B4GALT4 | TCMSP |
| daidzein | GHR | TCMSP |
| daidzein | TGFB1I1 | TCMSP |
| daidzein | CD5L | TCMSP |
| daidzein | P4HB | TCMSP |
| daidzein | CYP21A2 | TCMSP |
| daidzein | GADD45A | TCMSP |
| daidzein | BARD1 | TCMSP |
| daidzein | RBM45 | TCMSP |
| daidzein | BAP1 | TCMSP |
| daidzein | RAD51 | TCMSP |
| triptolide | RELA | TCMSP |
| triptolide | STAT3 | TCMSP |
| triptolide | VEGFA | TCMSP |
| triptolide | BCL2 | TCMSP |
| triptolide | FOS | TCMSP |
| triptolide | CDKN1A | TCMSP |
| triptolide | PLAU | TCMSP |
| triptolide | TNFSF15 | TCMSP |
| triptolide | JUN | TCMSP |
| triptolide | CASP3 | TCMSP |
| triptolide | TP63 | TCMSP |
| triptolide | MAPK8 | TCMSP |
| triptolide | PTGS2 | TCMSP |
| triptolide | STAT1 | TCMSP |
| triptolide | CXCL8 | TCMSP |
| triptolide | MCL1 | TCMSP |
| triptolide | IL2 | TCMSP |
| triptolide | IFNG | TCMSP |
| triptolide | IL4 | TCMSP |
| triptolide | CD80 | TCMSP |
| triptolide | CD86 | TCMSP |
| triptolide | CXCR4 | TCMSP |
| triptolide | BIRC3 | TCMSP |
| triptolide | CD274 | TCMSP |
| triptolide | IL23A | TCMSP |
| triptolide | CCR7 | TCMSP |
| triptolide | CD1A | TCMSP |
| triptolide | CD40 | TCMSP |
| triptolide | CD14 | TCMSP |
| triptolide | C3 | TCMSP |
| triptolide | VTCN1 | TCMSP |
| quercetin | NOX4 | SwissTargetPrediction |
| quercetin | AVPR2 | SwissTargetPrediction |
| quercetin | AKR1B1 | TCMSP |
| quercetin | XDH | SwissTargetPrediction |
| quercetin | MAOA | SwissTargetPrediction |
| quercetin | IGF1R | SwissTargetPrediction |
| quercetin | FLT3 | SwissTargetPrediction |
| quercetin | CYP19A1 | SwissTargetPrediction |
| quercetin | EGFR | TCMSP |
| quercetin | F2 | SwissTargetPrediction |
| quercetin | CA2 | SwissTargetPrediction |
| quercetin | PIM1 | SwissTargetPrediction |
| quercetin | ALOX5 | TCMSP |
| quercetin | AURKB | SwissTargetPrediction |
| quercetin | DRD4 | SwissTargetPrediction |
| quercetin | ADORA1 | SwissTargetPrediction |
| quercetin | CA7 | SwissTargetPrediction |
| quercetin | GLO1 | SwissTargetPrediction |
| quercetin | MPO | TCMSP |
| quercetin | PIK3R1 | SwissTargetPrediction |
| quercetin | ADORA2A | SwissTargetPrediction |
| quercetin | DAPK1 | SwissTargetPrediction |
| quercetin | PYGL | SwissTargetPrediction |
| quercetin | CA1 | SwissTargetPrediction |
| quercetin | GSK3B | SwissTargetPrediction |
| quercetin | SRC | SwissTargetPrediction |
| quercetin | PTK2 | SwissTargetPrediction |
| quercetin | HSD17B2 | SwissTargetPrediction |
| quercetin | KDR | SwissTargetPrediction |
| quercetin | MMP13 | SwissTargetPrediction |
| quercetin | MMP3 | TCMSP |
| quercetin | CA3 | SwissTargetPrediction |
| quercetin | ALOX15 | SwissTargetPrediction |
| quercetin | ABCC1 | SwissTargetPrediction |
| quercetin | PLK1 | SwissTargetPrediction |
| quercetin | CA6 | SwissTargetPrediction |
| quercetin | CDK1 | SwissTargetPrediction |
| quercetin | MMP9 | TCMSP |
| quercetin | CA12 | SwissTargetPrediction |
| quercetin | MMP2 | TCMSP |
| quercetin | PKN1 | SwissTargetPrediction |
| quercetin | CA14 | SwissTargetPrediction |
| quercetin | CA9 | SwissTargetPrediction |
| quercetin | CSNK2A1 | SwissTargetPrediction |
| quercetin | ALOX12 | SwissTargetPrediction |
| quercetin | MET | SwissTargetPrediction |
| quercetin | CA4 | SwissTargetPrediction |
| quercetin | NEK2 | SwissTargetPrediction |
| quercetin | CXCR1 | SwissTargetPrediction |
| quercetin | CAMK2B | SwissTargetPrediction |
| quercetin | ALK | SwissTargetPrediction |
| quercetin | AKT1 | TCMSP |
| quercetin | ABCB1 | SwissTargetPrediction |
| quercetin | NEK6 | SwissTargetPrediction |
| quercetin | PLA2G1B | SwissTargetPrediction |
| quercetin | CA5A | SwissTargetPrediction |
| quercetin | BACE1 | SwissTargetPrediction |
| quercetin | CYP1B1 | TCMSP |
| quercetin | AXL | SwissTargetPrediction |
| quercetin | ABCG2 | TCMSP |
| quercetin | NUAK1 | SwissTargetPrediction |
| quercetin | AKR1C2 | SwissTargetPrediction |
| quercetin | AKR1C1 | SwissTargetPrediction |
| quercetin | AKR1C3 | SwissTargetPrediction |
| quercetin | AKR1C4 | SwissTargetPrediction |
| quercetin | CA13 | SwissTargetPrediction |
| quercetin | AKR1A1 | SwissTargetPrediction |
| quercetin | GPR35 | SwissTargetPrediction |
| quercetin | SYK | SwissTargetPrediction |
| quercetin | MAPT | SwissTargetPrediction |
| quercetin | KDM4E | SwissTargetPrediction |
| quercetin | TOP2A | TCMSP |
| quercetin | INSR | TCMSP |
| quercetin | ACHE | TCMSP |
| quercetin | MYLK | SwissTargetPrediction |
| quercetin | PIK3CG | SwissTargetPrediction |
| quercetin | APEX1 | SwissTargetPrediction |
| quercetin | CDK5R1 CDK5 | SwissTargetPrediction |
| quercetin | CCNB3 CDK1 CCNB1 CCNB2 | SwissTargetPrediction |
| quercetin | ARG1 | SwissTargetPrediction |
| quercetin | PTPRS | SwissTargetPrediction |
| quercetin | ESR2 | SwissTargetPrediction |
| quercetin | MPG | SwissTargetPrediction |
| quercetin | SLC22A12 | SwissTargetPrediction |
| quercetin | CDK6 | SwissTargetPrediction |
| quercetin | CDK2 | SwissTargetPrediction |
| quercetin | TYR | SwissTargetPrediction |
| quercetin | HSD17B1 | SwissTargetPrediction |
| quercetin | AHR | TCMSP |
| quercetin | ESRRA | SwissTargetPrediction |
| quercetin | APP | SwissTargetPrediction |
| quercetin | PARP1 | TCMSP |
| quercetin | TTR | SwissTargetPrediction |
| quercetin | MMP12 | SwissTargetPrediction |
| quercetin | CD38 | SwissTargetPrediction |
| quercetin | AKR1B10 | SwissTargetPrediction |
| quercetin | TNKS2 | SwissTargetPrediction |
| quercetin | TNKS | SwissTargetPrediction |
| quercetin | TOP1 | TCMSP |
| quercetin | TERT | SwissTargetPrediction |
| quercetin | PTGS1 | TCMSP |
| quercetin | AR | TCMSP |
| quercetin | PPARG | TCMSP |
| quercetin | PTGS2 | TCMSP |
| quercetin | NCOA2 | TCMSP |
| quercetin | PRSS1 | TCMSP |
| quercetin | KCNH2 | TCMSP |
| quercetin | SCN5A | TCMSP |
| quercetin | ADRB2 | TCMSP |
| quercetin | F7 | TCMSP |
| quercetin | RXRA | TCMSP |
| quercetin | GABRA1 | TCMSP |
| quercetin | MAOB | TCMSP |
| quercetin | RELA | TCMSP |
| quercetin | VEGFA | TCMSP |
| quercetin | CCND1 | TCMSP |
| quercetin | BCL2 | TCMSP |
| quercetin | BCL2L1 | TCMSP |
| quercetin | FOS | TCMSP |
| quercetin | CDKN1A | TCMSP |
| quercetin | EIF6 | TCMSP |
| quercetin | BAX | TCMSP |
| quercetin | CASP9 | TCMSP |
| quercetin | PLAU | TCMSP |
| quercetin | MAPK1 | TCMSP |
| quercetin | IL10 | TCMSP |
| quercetin | EGF | TCMSP |
| quercetin | RB1 | TCMSP |
| quercetin | TNFSF15 | TCMSP |
| quercetin | JUN | TCMSP |
| quercetin | IL6 | TCMSP |
| quercetin | AHSA1 | TCMSP |
| quercetin | CASP3 | TCMSP |
| quercetin | TP63 | TCMSP |
| quercetin | ELK1 | TCMSP |
| quercetin | NFKBIA | TCMSP |
| quercetin | POR | TCMSP |
| quercetin | ODC1 | TCMSP |
| quercetin | CASP8 | TCMSP |
| quercetin | RAF1 | TCMSP |
| quercetin | SOD1 | TCMSP |
| quercetin | PRKCA | TCMSP |
| quercetin | MMP1 | TCMSP |
| quercetin | HIF1A | TCMSP |
| quercetin | STAT1 | TCMSP |
| quercetin | RUNX1T1 | TCMSP |
| quercetin | ERBB2 | TCMSP |
| quercetin | ACACA | TCMSP |
| quercetin | HMOX1 | TCMSP |
| quercetin | CYP3A4 | TCMSP |
| quercetin | CYP1A2 | TCMSP |
| quercetin | CAV1 | TCMSP |
| quercetin | MYC | TCMSP |
| quercetin | F3 | TCMSP |
| quercetin | GJA1 | TCMSP |
| quercetin | CYP1A1 | TCMSP |
| quercetin | ICAM1 | TCMSP |
| quercetin | IL1B | TCMSP |
| quercetin | CCL2 | TCMSP |
| quercetin | SELE | TCMSP |
| quercetin | VCAM1 | TCMSP |
| quercetin | PTGER3 | TCMSP |
| quercetin | CXCL8 | TCMSP |
| quercetin | PRKCB | TCMSP |
| quercetin | BIRC5 | TCMSP |
| quercetin | DUOX2 | TCMSP |
| quercetin | NOS3 | TCMSP |
| quercetin | HSPB1 | TCMSP |
| quercetin | SULT1E1 | TCMSP |
| quercetin | MGAM | TCMSP |
| quercetin | IL2 | TCMSP |
| quercetin | NR1I2 | TCMSP |
| quercetin | CCNB1 | TCMSP |
| quercetin | PLAT | TCMSP |
| quercetin | THBD | TCMSP |
| quercetin | SERPINE1 | TCMSP |
| quercetin | COL1A1 | TCMSP |
| quercetin | IFNG | TCMSP |
| quercetin | IL1A | TCMSP |
| quercetin | NCF1 | TCMSP |
| quercetin | HAS2 | TCMSP |
| quercetin | GSTP1 | TCMSP |
| quercetin | NFE2L2 | TCMSP |
| quercetin | NQO1 | TCMSP |
| quercetin | PSMD3 | TCMSP |
| quercetin | SLC2A4 | TCMSP |
| quercetin | COL3A1 | TCMSP |
| quercetin | CXCL11 | TCMSP |
| quercetin | CXCL2 | TCMSP |
| quercetin | DCAF5 | TCMSP |
| quercetin | NR1I3 | TCMSP |
| quercetin | CHEK2 | TCMSP |
| quercetin | CLDN4 | TCMSP |
| quercetin | PPARA | TCMSP |
| quercetin | PPARD | TCMSP |
| quercetin | HSF1 | TCMSP |
| quercetin | CRP | TCMSP |
| quercetin | CXCL10 | TCMSP |
| quercetin | CHUK | TCMSP |
| quercetin | SPP1 | TCMSP |
| quercetin | RUNX2 | TCMSP |
| quercetin | RASSF1 | TCMSP |
| quercetin | E2F1 | TCMSP |
| quercetin | E2F2 | TCMSP |
| quercetin | ACPP | TCMSP |
| quercetin | CTSD | TCMSP |
| quercetin | IGFBP3 | TCMSP |
| quercetin | IGF2 | TCMSP |
| quercetin | CD40LG | TCMSP |
| quercetin | IRF1 | TCMSP |
| quercetin | ERBB3 | TCMSP |
| quercetin | PON1 | TCMSP |
| quercetin | DIO1 | TCMSP |
| quercetin | PCOLCE | TCMSP |
| quercetin | NPEPPS | TCMSP |
| quercetin | HK2 | TCMSP |
| quercetin | RASA1 | TCMSP |
| quercetin | GSTM1 | TCMSP |
| quercetin | GSTM2 | TCMSP |
| luteolin | NOX4 | SwissTargetPrediction |
| luteolin | AKR1B1 | SwissTargetPrediction |
| luteolin | CDK5R1 CDK5 | SwissTargetPrediction |
| luteolin | XDH | SwissTargetPrediction |
| luteolin | MAOA | SwissTargetPrediction |
| luteolin | FLT3 | SwissTargetPrediction |
| luteolin | CA2 | SwissTargetPrediction |
| luteolin | CCNB3 CDK1 CCNB1 CCNB2 | SwissTargetPrediction |
| luteolin | ALOX5 | SwissTargetPrediction |
| luteolin | ADORA1 | SwissTargetPrediction |
| luteolin | CA7 | SwissTargetPrediction |
| luteolin | GLO1 | SwissTargetPrediction |
| luteolin | APP | SwissTargetPrediction |
| luteolin | SYK | SwissTargetPrediction |
| luteolin | GSK3B | SwissTargetPrediction |
| luteolin | PARP1 | SwissTargetPrediction |
| luteolin | TTR | SwissTargetPrediction |
| luteolin | MMP9 | SwissTargetPrediction |
| luteolin | CA12 | SwissTargetPrediction |
| luteolin | MMP2 | SwissTargetPrediction |
| luteolin | CA4 | SwissTargetPrediction |
| luteolin | MMP12 | SwissTargetPrediction |
| luteolin | CD38 | SwissTargetPrediction |
| luteolin | CYP1B1 | SwissTargetPrediction |
| luteolin | ABCG2 | SwissTargetPrediction |
| luteolin | AKR1B10 | SwissTargetPrediction |
| luteolin | TNKS2 | SwissTargetPrediction |
| luteolin | TNKS | SwissTargetPrediction |
| luteolin | TOP1 | SwissTargetPrediction |
| luteolin | ARG1 | SwissTargetPrediction |
| luteolin | PTPRS | SwissTargetPrediction |
| luteolin | ABCC1 | SwissTargetPrediction |
| luteolin | HSD17B1 | SwissTargetPrediction |
| luteolin | ACHE | SwissTargetPrediction |
| luteolin | CDK6 | SwissTargetPrediction |
| luteolin | ABCB1 | SwissTargetPrediction |
| luteolin | HSD17B2 | SwissTargetPrediction |
| luteolin | ALOX15 | SwissTargetPrediction |
| luteolin | ALOX12 | SwissTargetPrediction |
| luteolin | ESR2 | SwissTargetPrediction |
| luteolin | CYP19A1 | SwissTargetPrediction |
| luteolin | ADORA2A | SwissTargetPrediction |
| luteolin | CSNK2A1 | SwissTargetPrediction |
| luteolin | ESR1 | SwissTargetPrediction |
| luteolin | PTGS2 | SwissTargetPrediction |
| luteolin | CFTR | SwissTargetPrediction |
| luteolin | AMY1A | SwissTargetPrediction |
| luteolin | GRK6 | SwissTargetPrediction |
| luteolin | CDK2 | SwissTargetPrediction |
| luteolin | TERT | SwissTargetPrediction |
| luteolin | CA1 | SwissTargetPrediction |
| luteolin | CA9 | SwissTargetPrediction |
| luteolin | CDK1 | SwissTargetPrediction |
| luteolin | TYR | SwissTargetPrediction |
| luteolin | AHR | SwissTargetPrediction |
| luteolin | ESRRA | SwissTargetPrediction |
| luteolin | GPR35 | SwissTargetPrediction |
| luteolin | DAPK1 | SwissTargetPrediction |
| luteolin | AVPR2 | SwissTargetPrediction |
| luteolin | IGF1R | SwissTargetPrediction |
| luteolin | EGFR | SwissTargetPrediction |
| luteolin | F2 | SwissTargetPrediction |
| luteolin | PIM1 | SwissTargetPrediction |
| luteolin | AURKB | SwissTargetPrediction |
| luteolin | DRD4 | SwissTargetPrediction |
| luteolin | MPO | SwissTargetPrediction |
| luteolin | PIK3R1 | SwissTargetPrediction |
| luteolin | PYGL | SwissTargetPrediction |
| luteolin | SRC | SwissTargetPrediction |
| luteolin | PTK2 | SwissTargetPrediction |
| luteolin | KDR | SwissTargetPrediction |
| luteolin | MMP13 | SwissTargetPrediction |
| luteolin | MMP3 | SwissTargetPrediction |
| luteolin | CA3 | SwissTargetPrediction |
| luteolin | PLK1 | SwissTargetPrediction |
| luteolin | CA6 | SwissTargetPrediction |
| luteolin | PKN1 | SwissTargetPrediction |
| luteolin | CA14 | SwissTargetPrediction |
| luteolin | MET | SwissTargetPrediction |
| luteolin | NEK2 | SwissTargetPrediction |
| luteolin | CXCR1 | SwissTargetPrediction |
| luteolin | CAMK2B | SwissTargetPrediction |
| luteolin | ALK | SwissTargetPrediction |
| luteolin | AKT1 | SwissTargetPrediction |
| luteolin | NEK6 | SwissTargetPrediction |
| luteolin | PLA2G1B | SwissTargetPrediction |
| luteolin | CA5A | SwissTargetPrediction |
| luteolin | BACE1 | SwissTargetPrediction |
| luteolin | AXL | SwissTargetPrediction |
| luteolin | NUAK1 | SwissTargetPrediction |
| luteolin | AKR1C2 | SwissTargetPrediction |
| luteolin | AKR1C1 | SwissTargetPrediction |
| luteolin | AKR1C3 | SwissTargetPrediction |
| luteolin | AKR1C4 | SwissTargetPrediction |
| luteolin | CA13 | SwissTargetPrediction |
| luteolin | AKR1A1 | SwissTargetPrediction |
| luteolin | PFKFB3 | SwissTargetPrediction |
| luteolin | KDM4E | SwissTargetPrediction |
| luteolin | PLG | SwissTargetPrediction |
| luteolin | AR | SwissTargetPrediction |
| luteolin | PTGS1 | SwissTargetPrediction |
| luteolin | PRSS1 | SwissTargetPrediction |
| luteolin | NCOA2 | SwissTargetPrediction |
| luteolin | RELA | SwissTargetPrediction |
| luteolin | VEGFA | SwissTargetPrediction |
| luteolin | CCND1 | SwissTargetPrediction |
| luteolin | BCL2L1 | SwissTargetPrediction |
| luteolin | CDKN1A | SwissTargetPrediction |
| luteolin | CASP9 | SwissTargetPrediction |
| luteolin | MAPK1 | SwissTargetPrediction |
| luteolin | IL10 | SwissTargetPrediction |
| luteolin | RB1 | SwissTargetPrediction |
| luteolin | TNFSF15 | SwissTargetPrediction |
| luteolin | JUN | SwissTargetPrediction |
| luteolin | IL6 | SwissTargetPrediction |
| luteolin | CASP3 | SwissTargetPrediction |
| luteolin | TP63 | SwissTargetPrediction |
| luteolin | NFKBIA | SwissTargetPrediction |
| luteolin | MDM2 | SwissTargetPrediction |
| luteolin | MMP1 | SwissTargetPrediction |
| luteolin | PCNA | SwissTargetPrediction |
| luteolin | ERBB2 | SwissTargetPrediction |
| luteolin | PPARG | SwissTargetPrediction |
| luteolin | HMOX1 | SwissTargetPrediction |
| luteolin | CASP7 | SwissTargetPrediction |
| luteolin | ICAM1 | SwissTargetPrediction |
| luteolin | MCL1 | SwissTargetPrediction |
| luteolin | BIRC5 | SwissTargetPrediction |
| luteolin | IL2 | SwissTargetPrediction |
| luteolin | CCNB1 | SwissTargetPrediction |
| luteolin | IFNG | SwissTargetPrediction |
| luteolin | IL4 | SwissTargetPrediction |
| luteolin | TOP2A | SwissTargetPrediction |
| luteolin | GSTP1 | SwissTargetPrediction |
| luteolin | SLC2A4 | SwissTargetPrediction |
| luteolin | INSR | SwissTargetPrediction |
| luteolin | CD40LG | SwissTargetPrediction |
| luteolin | PTGES | SwissTargetPrediction |
| luteolin | NUF2 | SwissTargetPrediction |
| luteolin | ADCY2 | SwissTargetPrediction |
| epimedin a | PDE5A | SwissTargetPrediction |
| epimedin a | RPS6KA3 | SwissTargetPrediction |
| epimedin a | AKR1B1 | SwissTargetPrediction |
| epimedin a | ACHE | SwissTargetPrediction |
| epimedin a | CA7 | SwissTargetPrediction |
| epimedin a | NQO2 | SwissTargetPrediction |
| epimedin a | NOX4 | SwissTargetPrediction |
| epimedin a | ADRA2C | SwissTargetPrediction |
| epimedin a | NMUR2 | SwissTargetPrediction |
| epimedin a | ADRA2A | SwissTargetPrediction |
| epimedin a | CA2 | SwissTargetPrediction |
| epimedin a | PTGS2 | SwissTargetPrediction |
| epimedin a | XDH | SwissTargetPrediction |
| epimedin a | CD38 | SwissTargetPrediction |
| epimedin a | CA4 | SwissTargetPrediction |
| epimedin a | CA12 | SwissTargetPrediction |
| epimedin a | TNF | SwissTargetPrediction |
| epimedin a | VEGFA | SwissTargetPrediction |
| epimedin a | FGF1 | SwissTargetPrediction |
| epimedin a | FGF2 | SwissTargetPrediction |
| epimedin a | HPSE | SwissTargetPrediction |
| epimedin a | ABCB1 | SwissTargetPrediction |
| epimedin a | PRSS1 | SwissTargetPrediction |
| epimedin a | PRSS3 | SwissTargetPrediction |
| epimedin a | ALOX5 | SwissTargetPrediction |
| epimedin a | IL2 | SwissTargetPrediction |
| epimedin a | AMY2A | SwissTargetPrediction |
| epimedin a | ADORA1 | SwissTargetPrediction |
| epimedin a | F10 | SwissTargetPrediction |
| epimedin a | TYMS | SwissTargetPrediction |
| epimedin a | FPGS | SwissTargetPrediction |
| epimedin a | TACR2 | SwissTargetPrediction |
| epimedin a | CYP19A1 | SwissTargetPrediction |
| epimedin a | NPC1L1 | SwissTargetPrediction |
| epimedin a | TERT | SwissTargetPrediction |
| epimedin a | PTPRA | SwissTargetPrediction |
| epimedin a | ITGAV ITGB3 | SwissTargetPrediction |
| epimedin a | ITGA2B ITGB3 | SwissTargetPrediction |
| epimedin a | CASP3 | SwissTargetPrediction |
| epimedin a | CASP6 | SwissTargetPrediction |
| epimedin a | CASP7 | SwissTargetPrediction |
| epimedin a | CASP1 | SwissTargetPrediction |
| epimedin a | FOLH1 | SwissTargetPrediction |
| epimedin a | GBA | SwissTargetPrediction |
| epimedin a | GBA2 | SwissTargetPrediction |
| epimedin a | PTPN2 | SwissTargetPrediction |
| epimedin a | PTPN22 | SwissTargetPrediction |
| epimedin a | ATP1A1 | SwissTargetPrediction |
| epimedin a | HLA-A | SwissTargetPrediction |
| epimedin a | CTSD | SwissTargetPrediction |
| epimedin a | EDNRA | SwissTargetPrediction |
| epimedin a | LGALS3 | SwissTargetPrediction |
| epimedin a | CCKBR | SwissTargetPrediction |
| epimedin a | CASP8 | SwissTargetPrediction |
| epimedin a | ALOX5AP | SwissTargetPrediction |
| epimedin a | GPR34 | SwissTargetPrediction |
| epimedin a | ABCG2 | SwissTargetPrediction |
| epimedin a | SPTLC1 | SwissTargetPrediction |
| epimedin a | SPTLC2 | SwissTargetPrediction |
| epimedin a | ROCK2 | SwissTargetPrediction |
| epimedin a | FLT3 | SwissTargetPrediction |
| epimedin a | ALDH2 | SwissTargetPrediction |
| epimedin a | TBXAS1 | SwissTargetPrediction |
| epimedin a | P2RY12 | SwissTargetPrediction |
| epimedin a | AKT1 | SwissTargetPrediction |
| epimedin a | OPRK1 | SwissTargetPrediction |
| epimedin a | SIRT1 | SwissTargetPrediction |
| epimedin a | CYP1B1 | SwissTargetPrediction |
| epimedin a | DHFR | SwissTargetPrediction |
| epimedin a | ITGA2 ITGB3 | SwissTargetPrediction |
| epimedin a | PRKCB | SwissTargetPrediction |
| epimedin a | FDFT1 | SwissTargetPrediction |
| epimedin a | ITGB5 ITGAV | SwissTargetPrediction |
| epimedin a | TMPRSS11D | SwissTargetPrediction |
| epimedin a | ITGB1 ITGA5 | SwissTargetPrediction |
| epimedin a | DRD4 | SwissTargetPrediction |
| epimedin a | DRD3 | SwissTargetPrediction |
| epimedin a | ST14 | SwissTargetPrediction |
| epimedin a | PTPRC | SwissTargetPrediction |
| epimedin a | P2RY10 | SwissTargetPrediction |
| epimedin a | RRM1 | SwissTargetPrediction |
| epimedin a | LGALS4 | SwissTargetPrediction |
| epimedin a | SERPINE1 | SwissTargetPrediction |
| epimedin a | LGALS8 | SwissTargetPrediction |
| epimedin a | TNNC1 TNNT2 TNNI3 | SwissTargetPrediction |
| epimedin a | CD22 | SwissTargetPrediction |
| epimedin a | LGALS7 | SwissTargetPrediction |
| epimedin a | LGALS9 | SwissTargetPrediction |
| epimedin a | TMPRSS15 | SwissTargetPrediction |
| epimedin a | KCNA3 | SwissTargetPrediction |
| epimedin a | PDYN | SwissTargetPrediction |
| epimedin a | CA14 | SwissTargetPrediction |
| epimedin a | GRB2 | SwissTargetPrediction |
| epimedin a | MTOR | SwissTargetPrediction |
| epimedin a | PIK3CA | SwissTargetPrediction |
| epimedin a | PLG | SwissTargetPrediction |
| epimedin a | RORC | SwissTargetPrediction |
| epimedin a | LIPC | SwissTargetPrediction |
| epimedin a | STAT3 | SwissTargetPrediction |
| epimedin a | BCHE | SwissTargetPrediction |
| epimedin b | PDE5A | SwissTargetPrediction |
| epimedin b | ADRB2 | SwissTargetPrediction |
| epimedin b | IGF1R | SwissTargetPrediction |
| epimedin b | VEGFA | SwissTargetPrediction |
| epimedin b | FGF1 | SwissTargetPrediction |
| epimedin b | FGF2 | SwissTargetPrediction |
| epimedin b | HPSE | SwissTargetPrediction |
| epimedin b | F10 | SwissTargetPrediction |
| epimedin b | ABCB1 | SwissTargetPrediction |
| epimedin b | ADORA1 | SwissTargetPrediction |
| epimedin b | PRSS1 | SwissTargetPrediction |
| epimedin b | PRSS3 | SwissTargetPrediction |
| epimedin b | IMPDH1 | SwissTargetPrediction |
| epimedin b | IMPDH2 | SwissTargetPrediction |
| epimedin b | PTPN2 | SwissTargetPrediction |
| epimedin b | PTPRA | SwissTargetPrediction |
| epimedin b | IL2 | SwissTargetPrediction |
| epimedin b | TACR2 | SwissTargetPrediction |
| epimedin b | HDAC6 | SwissTargetPrediction |
| epimedin b | HDAC8 | SwissTargetPrediction |
| epimedin b | HDAC1 | SwissTargetPrediction |
| epimedin b | NPC1L1 | SwissTargetPrediction |
| epimedin b | AKT2 | SwissTargetPrediction |
| epimedin b | RPS6KA1 | SwissTargetPrediction |
| epimedin b | ROCK1 | SwissTargetPrediction |
| epimedin b | ALOX5 | SwissTargetPrediction |
| epimedin b | RRM1 | SwissTargetPrediction |
| epimedin b | ITGAV ITGB3 | SwissTargetPrediction |
| epimedin b | ITGA2B ITGB3 | SwissTargetPrediction |
| epimedin b | GRB2 | SwissTargetPrediction |
| epimedin b | ITGAV ITGB6 | SwissTargetPrediction |
| epimedin b | CNR1 | SwissTargetPrediction |
| epimedin b | CNR2 | SwissTargetPrediction |
| epimedin b | FPGS | SwissTargetPrediction |
| epimedin b | MMP2 | SwissTargetPrediction |
| epimedin b | MMP12 | SwissTargetPrediction |
| epimedin b | TYMS | SwissTargetPrediction |
| epimedin b | GBA | SwissTargetPrediction |
| epimedin b | GBA2 | SwissTargetPrediction |
| epimedin b | CASP3 | SwissTargetPrediction |
| epimedin b | EPHX2 | SwissTargetPrediction |
| epimedin b | F2 | SwissTargetPrediction |
| epimedin b | MMP13 | SwissTargetPrediction |
| epimedin b | LTB4R | SwissTargetPrediction |
| epimedin b | ITGB5 ITGAV | SwissTargetPrediction |
| epimedin b | ITGAV ITGB1 | SwissTargetPrediction |
| epimedin b | OPRK1 | SwissTargetPrediction |
| epimedin b | MMP9 | SwissTargetPrediction |
| epimedin b | MMP8 | SwissTargetPrediction |
| epimedin b | KDR | SwissTargetPrediction |
| epimedin b | CASP6 | SwissTargetPrediction |
| epimedin b | CASP7 | SwissTargetPrediction |
| epimedin b | CASP8 | SwissTargetPrediction |
| epimedin b | CASP1 | SwissTargetPrediction |
| epimedin b | FOLH1 | SwissTargetPrediction |
| epimedin b | ITGA2 ITGB3 | SwissTargetPrediction |
| epimedin b | S1PR1 | SwissTargetPrediction |
| epimedin b | P2RY12 | SwissTargetPrediction |
| epimedin b | PPARA | SwissTargetPrediction |
| epimedin b | CD22 | SwissTargetPrediction |
| epimedin b | SIRT1 | SwissTargetPrediction |
| epimedin b | CYP19A1 | SwissTargetPrediction |
| epimedin b | FNTA FNTB | SwissTargetPrediction |
| epimedin b | S1PR3 | SwissTargetPrediction |
| epimedin b | TMPRSS15 | SwissTargetPrediction |
| epimedin b | F3 F7 | SwissTargetPrediction |
| epimedin b | CTSD | SwissTargetPrediction |
| epimedin b | ITGB1 ITGA5 | SwissTargetPrediction |
| epimedin b | CAPN1 | SwissTargetPrediction |
| epimedin b | ITGAL | SwissTargetPrediction |
| epimedin b | STAT3 | SwissTargetPrediction |
| epimedin b | HMGCR | SwissTargetPrediction |
| epimedin b | SRD5A1 | SwissTargetPrediction |
| epimedin b | ITGA2B | SwissTargetPrediction |
| epimedin b | HLA-A | SwissTargetPrediction |
| epimedin b | F11 | SwissTargetPrediction |
| epimedin b | CDK2 | SwissTargetPrediction |
| epimedin b | PLK1 | SwissTargetPrediction |
| epimedin b | CDK1 | SwissTargetPrediction |
| epimedin b | F7 | SwissTargetPrediction |
| epimedin b | INSR | SwissTargetPrediction |
| epimedin b | TMPRSS11D | SwissTargetPrediction |
| epimedin b | GLO1 | SwissTargetPrediction |
| epimedin b | NMUR2 | SwissTargetPrediction |
| epimedin b | ADRA2A | SwissTargetPrediction |
| epimedin b | ADRA2C | SwissTargetPrediction |
| epimedin b | ACHE | SwissTargetPrediction |
| epimedin b | RPS6KA3 | SwissTargetPrediction |
| epimedin b | NQO2 | SwissTargetPrediction |
| epimedin b | NOX4 | SwissTargetPrediction |
| epimedin b | PTGS2 | SwissTargetPrediction |
| epimedin b | CA2 | SwissTargetPrediction |
| epimedin b | CA7 | SwissTargetPrediction |
| epimedin b | CA12 | SwissTargetPrediction |
| epimedin b | CA4 | SwissTargetPrediction |
| epimedin b | XDH | SwissTargetPrediction |
| epimedin b | AKR1B1 | SwissTargetPrediction |
| epimedin b | CD38 | SwissTargetPrediction |
| epimedin b | TNF | SwissTargetPrediction |
| epimedin b | TOP1 | SwissTargetPrediction |
| epimedin c | PDE5A | SwissTargetPrediction |
| epimedin c | NMUR2 | SwissTargetPrediction |
| epimedin c | ADRA2A | SwissTargetPrediction |
| epimedin c | ADRA2C | SwissTargetPrediction |
| epimedin c | ACHE | SwissTargetPrediction |
| epimedin c | RPS6KA3 | SwissTargetPrediction |
| epimedin c | CA7 | SwissTargetPrediction |
| epimedin c | NQO2 | SwissTargetPrediction |
| epimedin c | NOX4 | SwissTargetPrediction |
| epimedin c | CA2 | SwissTargetPrediction |
| epimedin c | PTGS2 | SwissTargetPrediction |
| epimedin c | AKR1B1 | SwissTargetPrediction |
| epimedin c | XDH | SwissTargetPrediction |
| epimedin c | CD38 | SwissTargetPrediction |
| epimedin c | CA4 | SwissTargetPrediction |
| epimedin c | CA12 | SwissTargetPrediction |
| epimedin c | TNF | SwissTargetPrediction |
| epimedin c | VEGFA | SwissTargetPrediction |
| epimedin c | FGF1 | SwissTargetPrediction |
| epimedin c | FGF2 | SwissTargetPrediction |
| epimedin c | HPSE | SwissTargetPrediction |
| epimedin c | ABCB1 | SwissTargetPrediction |
| epimedin c | ITGAV ITGB3 | SwissTargetPrediction |
| epimedin c | ADORA1 | SwissTargetPrediction |
| epimedin c | F10 | SwissTargetPrediction |
| epimedin c | ALOX5 | SwissTargetPrediction |
| epimedin c | IL2 | SwissTargetPrediction |
| epimedin c | LGALS3 | SwissTargetPrediction |
| epimedin c | TYMS | SwissTargetPrediction |
| epimedin c | CASP3 | SwissTargetPrediction |
| epimedin c | GRB2 | SwissTargetPrediction |
| epimedin c | PTPRA | SwissTargetPrediction |
| epimedin c | FOLH1 | SwissTargetPrediction |
| epimedin c | ITGA2B ITGB3 | SwissTargetPrediction |
| epimedin c | DHFR | SwissTargetPrediction |
| epimedin c | PTPN2 | SwissTargetPrediction |
| epimedin c | TACR2 | SwissTargetPrediction |
| epimedin c | RRM1 | SwissTargetPrediction |
| epimedin c | CASP6 | SwissTargetPrediction |
| epimedin c | CASP7 | SwissTargetPrediction |
| epimedin c | CASP8 | SwissTargetPrediction |
| epimedin c | CASP1 | SwissTargetPrediction |
| epimedin c | ITGA2 ITGB3 | SwissTargetPrediction |
| epimedin c | TYR | SwissTargetPrediction |
| epimedin c | FLT3 | SwissTargetPrediction |
| epimedin c | GBA | SwissTargetPrediction |
| epimedin c | SIRT1 | SwissTargetPrediction |
| epimedin c | CYP19A1 | SwissTargetPrediction |
| epimedin c | GBA2 | SwissTargetPrediction |
| epimedin c | CD22 | SwissTargetPrediction |
| epimedin c | PTPRC | SwissTargetPrediction |
| epimedin c | PRSS1 | SwissTargetPrediction |
| epimedin c | HLA-A | SwissTargetPrediction |
| epimedin c | PRSS3 | SwissTargetPrediction |
| epimedin c | AMY2A | SwissTargetPrediction |
| epimedin c | OPRK1 | SwissTargetPrediction |
| epimedin c | P2RY12 | SwissTargetPrediction |
| epimedin c | ITGB1 ITGA5 | SwissTargetPrediction |
| epimedin c | HDAC6 | SwissTargetPrediction |
| epimedin c | TNNC1 TNNT2 TNNI3 | SwissTargetPrediction |
| epimedin c | ITGB5 ITGAV | SwissTargetPrediction |
| epimedin c | FANCF | SwissTargetPrediction |
| epimedin c | DRD4 | SwissTargetPrediction |
| epimedin c | DRD3 | SwissTargetPrediction |
| epimedin c | S1PR3 | SwissTargetPrediction |
| epimedin c | S1PR1 | SwissTargetPrediction |
| epimedin c | SLC28A2 | SwissTargetPrediction |
| epimedin c | TMPRSS15 | SwissTargetPrediction |
| epimedin c | PTPN22 | SwissTargetPrediction |
| epimedin c | F9 | SwissTargetPrediction |
| epimedin c | ITGA2B | SwissTargetPrediction |
| epimedin c | MET | SwissTargetPrediction |
| epimedin c | ADRB2 | SwissTargetPrediction |
| epimedin c | GPR34 | SwissTargetPrediction |
| epimedin c | P2RY10 | SwissTargetPrediction |
| epimedin c | GPR174 | SwissTargetPrediction |
| epimedin c | NPC1L1 | SwissTargetPrediction |
| epimedin c | LIPC | SwissTargetPrediction |
| epimedin c | HMGCR | SwissTargetPrediction |
| epimedin c | KCNH2 | SwissTargetPrediction |
| epimedin c | CCKBR | SwissTargetPrediction |
| epimedin c | CALCRL | SwissTargetPrediction |
| epimedin c | LGALS4 | SwissTargetPrediction |
| epimedin c | LGALS8 | SwissTargetPrediction |
| epimedin c | LNPEP | SwissTargetPrediction |
| epimedin c | MMP13 | SwissTargetPrediction |
| epimedin c | ERAP2 | SwissTargetPrediction |
| epimedin c | ERAP1 | SwissTargetPrediction |
| epimedin c | MTOR | SwissTargetPrediction |
| epimedin c | PIK3CG | SwissTargetPrediction |
| epimedin c | SERPINE1 | SwissTargetPrediction |
| epimedin c | PIK3CA | SwissTargetPrediction |
| epimedin c | TMPRSS11D | SwissTargetPrediction |
| epimedin c | ITGAV ITGB6 | SwissTargetPrediction |
| epimedin c | ST14 | SwissTargetPrediction |
| epimedin c | MMP12 | SwissTargetPrediction |
| epimedin c | MMP8 | SwissTargetPrediction |
| epimedin c | UGCG | SwissTargetPrediction |
| epimedin c | PDYN | SwissTargetPrediction |
| epimedin c | FPGS | SwissTargetPrediction |
| icariin | PDE5A | SwissTargetPrediction |
| icariin | RPS6KA3 | SwissTargetPrediction |
| icariin | ACHE | SwissTargetPrediction |
| icariin | NQO2 | SwissTargetPrediction |
| icariin | NOX4 | SwissTargetPrediction |
| icariin | ADRA2C | SwissTargetPrediction |
| icariin | CA2 | SwissTargetPrediction |
| icariin | CA12 | SwissTargetPrediction |
| icariin | NMUR2 | SwissTargetPrediction |
| icariin | ADRA2A | SwissTargetPrediction |
| icariin | AKR1B1 | SwissTargetPrediction |
| icariin | CA7 | SwissTargetPrediction |
| icariin | CA4 | SwissTargetPrediction |
| icariin | CD38 | SwissTargetPrediction |
| icariin | XDH | SwissTargetPrediction |
| icariin | EPHX2 | SwissTargetPrediction |
| icariin | PTGS2 | SwissTargetPrediction |
| icariin | IL2 | SwissTargetPrediction |
| icariin | ADORA1 | SwissTargetPrediction |
| icariin | TOP1 | SwissTargetPrediction |
| icariin | F2 | SwissTargetPrediction |
| icariin | F7 | SwissTargetPrediction |
| icariin | FOLH1 | SwissTargetPrediction |
| icariin | ABCB1 | SwissTargetPrediction |
| icariin | CSNK2A1 | SwissTargetPrediction |
| icariin | CSNK2A2 | SwissTargetPrediction |
| icariin | CA14 | SwissTargetPrediction |
| icariin | TACR2 | SwissTargetPrediction |
| icariin | F10 | SwissTargetPrediction |
| icariin | TYMS | SwissTargetPrediction |
| icariin | TYMP | SwissTargetPrediction |
| icariin | MAPK8 | SwissTargetPrediction |
| icariin | MAP2K1 | SwissTargetPrediction |
| icariin | MAPK9 | SwissTargetPrediction |
| icariin | ITGAV ITGB3 | SwissTargetPrediction |
| icariin | ITGA2B ITGB3 | SwissTargetPrediction |
| icariin | ITGB5 ITGAV | SwissTargetPrediction |
| icariin | ITGAV ITGB1 | SwissTargetPrediction |
| icariin | CD22 | SwissTargetPrediction |
| icariin | C3AR1 | SwissTargetPrediction |
| icariin | PRSS1 | SwissTargetPrediction |
| icariin | SLC28A2 | SwissTargetPrediction |
| icariin | F9 | SwissTargetPrediction |
| icariin | PPARA | SwissTargetPrediction |
| icariin | CDK2 | SwissTargetPrediction |
| icariin | CDK1 | SwissTargetPrediction |
| icariin | STAT3 | SwissTargetPrediction |
| icariin | BCL2A1 | SwissTargetPrediction |
| icariin | MMP2 | SwissTargetPrediction |
| icariin | HCAR2 | SwissTargetPrediction |
| icariin | MMP12 | SwissTargetPrediction |
| icariin | SLC29A1 | SwissTargetPrediction |
| icariin | MAPK14 | SwissTargetPrediction |
| icariin | PRKCA | SwissTargetPrediction |
| icariin | DNMT3B | SwissTargetPrediction |
| icariin | FDFT1 | SwissTargetPrediction |
| icariin | PRUNE1 | SwissTargetPrediction |
| icariin | GPR34 | SwissTargetPrediction |
| icariin | P2RY10 | SwissTargetPrediction |
| icariin | METAP2 | SwissTargetPrediction |
| icariin | TRPV1 | SwissTargetPrediction |
| icariin | JUN | SwissTargetPrediction |
| icariin | AKR1B10 | SwissTargetPrediction |
| icariin | IMPDH1 | SwissTargetPrediction |
| icariin | ABL1 | SwissTargetPrediction |
| icariin | KIT | SwissTargetPrediction |
| icariin | IMPDH2 | SwissTargetPrediction |
| icariin | PDGFRA PDGFRB | SwissTargetPrediction |
| icariin | ITGA2 ITGB3 | SwissTargetPrediction |
| icariin | FNTA FNTB | SwissTargetPrediction |
| icariin | FGF2 | SwissTargetPrediction |
| icariin | HSP90AA1 | SwissTargetPrediction |
| icariin | GLB1 | SwissTargetPrediction |
| icariin | PPP2CA | SwissTargetPrediction |
| icariin | ECE1 | SwissTargetPrediction |
| icariin | ACE | SwissTargetPrediction |
| icariin | F3 F7 | SwissTargetPrediction |
| icariin | GBA | SwissTargetPrediction |
| icariin | ROCK2 | SwissTargetPrediction |
| icariin | GBA2 | SwissTargetPrediction |
| icariin | PTPN2 | SwissTargetPrediction |
| icariin | PTPRA | SwissTargetPrediction |
| icariin | PRKACA | SwissTargetPrediction |
| icariin | AKT3 | SwissTargetPrediction |
| icariin | ITGB1 ITGA4 | SwissTargetPrediction |
| icariin | TNF | SwissTargetPrediction |
| icariin | ITGAL | SwissTargetPrediction |
| icariin | KCNH2 | SwissTargetPrediction |
| icariin | TMPRSS15 | SwissTargetPrediction |
| icariin | MMP13 | SwissTargetPrediction |
| icariin | MMP9 | SwissTargetPrediction |
| icariin | CAPN1 | SwissTargetPrediction |
| icariin | MMP8 | SwissTargetPrediction |
| icariin | DOT1L | SwissTargetPrediction |
| icariin | ITGB1 ITGA5 | SwissTargetPrediction |
| icariin | CDK6 | SwissTargetPrediction |
| icariin | PARP1 | SwissTargetPrediction |
| icariin | CDK4 | SwissTargetPrediction |
| icariin | NOS2 | SwissTargetPrediction |
| icariin | MANBA | SwissTargetPrediction |
| kaempferol | NOX4 | SwissTargetPrediction |
| kaempferol | AKR1B1 | SwissTargetPrediction |
| kaempferol | XDH | SwissTargetPrediction |
| kaempferol | TYR | SwissTargetPrediction |
| kaempferol | FLT3 | SwissTargetPrediction |
| kaempferol | CA2 | SwissTargetPrediction |
| kaempferol | ALOX5 | SwissTargetPrediction |
| kaempferol | CA7 | SwissTargetPrediction |
| kaempferol | HSD17B2 | SwissTargetPrediction |
| kaempferol | ABCC1 | SwissTargetPrediction |
| kaempferol | HSD17B1 | SwissTargetPrediction |
| kaempferol | AHR | SwissTargetPrediction |
| kaempferol | CA12 | SwissTargetPrediction |
| kaempferol | ESRRA | SwissTargetPrediction |
| kaempferol | ABCB1 | SwissTargetPrediction |
| kaempferol | CYP1B1 | SwissTargetPrediction |
| kaempferol | ABCG2 | SwissTargetPrediction |
| kaempferol | ADORA1 | SwissTargetPrediction |
| kaempferol | CA4 | SwissTargetPrediction |
| kaempferol | ACHE | SwissTargetPrediction |
| kaempferol | MAOA | SwissTargetPrediction |
| kaempferol | GLO1 | SwissTargetPrediction |
| kaempferol | SYK | SwissTargetPrediction |
| kaempferol | GSK3B | SwissTargetPrediction |
| kaempferol | MMP9 | SwissTargetPrediction |
| kaempferol | MMP2 | SwissTargetPrediction |
| kaempferol | ALOX15 | SwissTargetPrediction |
| kaempferol | ALOX12 | SwissTargetPrediction |
| kaempferol | PTPRS | SwissTargetPrediction |
| kaempferol | ADORA2A | SwissTargetPrediction |
| kaempferol | CDK5R1 CDK5 | SwissTargetPrediction |
| kaempferol | CCNB3 CDK1 CCNB1 CCNB2 | SwissTargetPrediction |
| kaempferol | ARG1 | SwissTargetPrediction |
| kaempferol | GPR35 | SwissTargetPrediction |
| kaempferol | ESR2 | SwissTargetPrediction |
| kaempferol | DAPK1 | SwissTargetPrediction |
| kaempferol | MPG | SwissTargetPrediction |
| kaempferol | SLC22A12 | SwissTargetPrediction |
| kaempferol | CDK6 | SwissTargetPrediction |
| kaempferol | CDK2 | SwissTargetPrediction |
| kaempferol | TTR | SwissTargetPrediction |
| kaempferol | AKR1B10 | SwissTargetPrediction |
| kaempferol | TNKS2 | SwissTargetPrediction |
| kaempferol | TNKS | SwissTargetPrediction |
| kaempferol | CYP19A1 | SwissTargetPrediction |
| kaempferol | CSNK2A1 | SwissTargetPrediction |
| kaempferol | EGFR | SwissTargetPrediction |
| kaempferol | AVPR2 | SwissTargetPrediction |
| kaempferol | IGF1R | SwissTargetPrediction |
| kaempferol | F2 | SwissTargetPrediction |
| kaempferol | PIM1 | SwissTargetPrediction |
| kaempferol | AURKB | SwissTargetPrediction |
| kaempferol | DRD4 | SwissTargetPrediction |
| kaempferol | MPO | SwissTargetPrediction |
| kaempferol | PIK3R1 | SwissTargetPrediction |
| kaempferol | PYGL | SwissTargetPrediction |
| kaempferol | CA1 | SwissTargetPrediction |
| kaempferol | SRC | SwissTargetPrediction |
| kaempferol | PTK2 | SwissTargetPrediction |
| kaempferol | KDR | SwissTargetPrediction |
| kaempferol | MMP13 | SwissTargetPrediction |
| kaempferol | MMP3 | SwissTargetPrediction |
| kaempferol | CA3 | SwissTargetPrediction |
| kaempferol | PLK1 | SwissTargetPrediction |
| kaempferol | CA6 | SwissTargetPrediction |
| kaempferol | CDK1 | SwissTargetPrediction |
| kaempferol | PKN1 | SwissTargetPrediction |
| kaempferol | CA14 | SwissTargetPrediction |
| kaempferol | CA9 | SwissTargetPrediction |
| kaempferol | MET | SwissTargetPrediction |
| kaempferol | NEK2 | SwissTargetPrediction |
| kaempferol | CXCR1 | SwissTargetPrediction |
| kaempferol | CAMK2B | SwissTargetPrediction |
| kaempferol | ALK | SwissTargetPrediction |
| kaempferol | AKT1 | SwissTargetPrediction |
| kaempferol | NEK6 | SwissTargetPrediction |
| kaempferol | PLA2G1B | SwissTargetPrediction |
| kaempferol | CA5A | SwissTargetPrediction |
| kaempferol | BACE1 | SwissTargetPrediction |
| kaempferol | AXL | SwissTargetPrediction |
| kaempferol | NUAK1 | SwissTargetPrediction |
| kaempferol | AKR1C2 | SwissTargetPrediction |
| kaempferol | AKR1C1 | SwissTargetPrediction |
| kaempferol | AKR1C3 | SwissTargetPrediction |
| kaempferol | AKR1C4 | SwissTargetPrediction |
| kaempferol | CA13 | SwissTargetPrediction |
| kaempferol | AKR1A1 | SwissTargetPrediction |
| kaempferol | APP | SwissTargetPrediction |
| kaempferol | PARP1 | SwissTargetPrediction |
| kaempferol | MMP12 | SwissTargetPrediction |
| kaempferol | CD38 | SwissTargetPrediction |
| kaempferol | TOP1 | SwissTargetPrediction |
| kaempferol | ESR1 | SwissTargetPrediction |
| kaempferol | PTGS2 | SwissTargetPrediction |
| kaempferol | CFTR | SwissTargetPrediction |
| kaempferol | PFKFB3 | SwissTargetPrediction |
| kaempferol | AMY1A | SwissTargetPrediction |
| kaempferol | GRK6 | SwissTargetPrediction |
| kaempferol | TERT | SwissTargetPrediction |
| kaempferol | BCHE | SwissTargetPrediction |
| kaempferol | NOS2 | SwissTargetPrediction |
| kaempferol | PTGS1 | SwissTargetPrediction |
| kaempferol | AR | SwissTargetPrediction |
| kaempferol | PPARG | SwissTargetPrediction |
| kaempferol | NCOA2 | SwissTargetPrediction |
| kaempferol | PRSS1 | SwissTargetPrediction |
| kaempferol | PGR | SwissTargetPrediction |
| kaempferol | CHRM1 | SwissTargetPrediction |
| kaempferol | SLC6A2 | SwissTargetPrediction |
| kaempferol | CHRM2 | SwissTargetPrediction |
| kaempferol | ADRA1B | SwissTargetPrediction |
| kaempferol | GABRA1 | SwissTargetPrediction |
| kaempferol | F7 | SwissTargetPrediction |
| kaempferol | RELA | SwissTargetPrediction |
| kaempferol | IKBKB | SwissTargetPrediction |
| kaempferol | BCL2 | SwissTargetPrediction |
| kaempferol | BAX | SwissTargetPrediction |
| kaempferol | TNFSF15 | SwissTargetPrediction |
| kaempferol | JUN | SwissTargetPrediction |
| kaempferol | AHSA1 | SwissTargetPrediction |
| kaempferol | CASP3 | SwissTargetPrediction |
| kaempferol | MAPK8 | SwissTargetPrediction |
| kaempferol | MMP1 | SwissTargetPrediction |
| kaempferol | STAT1 | SwissTargetPrediction |
| kaempferol | HMOX1 | SwissTargetPrediction |
| kaempferol | CYP3A4 | SwissTargetPrediction |
| kaempferol | CYP1A2 | SwissTargetPrediction |
| kaempferol | CYP1A1 | SwissTargetPrediction |
| kaempferol | ICAM1 | SwissTargetPrediction |
| kaempferol | SELE | SwissTargetPrediction |
| kaempferol | VCAM1 | SwissTargetPrediction |
| kaempferol | NR1I2 | SwissTargetPrediction |
| kaempferol | HAS2 | SwissTargetPrediction |
| kaempferol | GSTP1 | SwissTargetPrediction |
| kaempferol | PSMD3 | SwissTargetPrediction |
| kaempferol | SLC2A4 | SwissTargetPrediction |
| kaempferol | NR1I3 | SwissTargetPrediction |
| kaempferol | INSR | SwissTargetPrediction |
| kaempferol | DIO1 | SwissTargetPrediction |
| kaempferol | PPP3CA | SwissTargetPrediction |
| kaempferol | GSTM1 | SwissTargetPrediction |
| kaempferol | GSTM2 | SwissTargetPrediction |
| kaempferol | SLPI | SwissTargetPrediction |
| isorhamnetin | XDH | SwissTargetPrediction |
| isorhamnetin | CA2 | SwissTargetPrediction |
| isorhamnetin | CA7 | SwissTargetPrediction |
| isorhamnetin | CA12 | SwissTargetPrediction |
| isorhamnetin | CA4 | SwissTargetPrediction |
| isorhamnetin | CYP1B1 | SwissTargetPrediction |
| isorhamnetin | ABCC1 | SwissTargetPrediction |
| isorhamnetin | NOX4 | SwissTargetPrediction |
| isorhamnetin | AKR1B1 | SwissTargetPrediction |
| isorhamnetin | ABCG2 | SwissTargetPrediction |
| isorhamnetin | IGF1R | SwissTargetPrediction |
| isorhamnetin | EGFR | SwissTargetPrediction |
| isorhamnetin | ACHE | SwissTargetPrediction |
| isorhamnetin | ALOX15 | SwissTargetPrediction |
| isorhamnetin | ALOX12 | SwissTargetPrediction |
| isorhamnetin | AVPR2 | SwissTargetPrediction |
| isorhamnetin | MAOA | SwissTargetPrediction |
| isorhamnetin | FLT3 | SwissTargetPrediction |
| isorhamnetin | CYP19A1 | SwissTargetPrediction |
| isorhamnetin | F2 | SwissTargetPrediction |
| isorhamnetin | PIM1 | SwissTargetPrediction |
| isorhamnetin | ALOX5 | SwissTargetPrediction |
| isorhamnetin | AURKB | SwissTargetPrediction |
| isorhamnetin | DRD4 | SwissTargetPrediction |
| isorhamnetin | ADORA1 | SwissTargetPrediction |
| isorhamnetin | GLO1 | SwissTargetPrediction |
| isorhamnetin | MPO | SwissTargetPrediction |
| isorhamnetin | PIK3R1 | SwissTargetPrediction |
| isorhamnetin | ADORA2A | SwissTargetPrediction |
| isorhamnetin | DAPK1 | SwissTargetPrediction |
| isorhamnetin | PYGL | SwissTargetPrediction |
| isorhamnetin | CA1 | SwissTargetPrediction |
| isorhamnetin | GSK3B | SwissTargetPrediction |
| isorhamnetin | SRC | SwissTargetPrediction |
| isorhamnetin | PTK2 | SwissTargetPrediction |
| isorhamnetin | HSD17B2 | SwissTargetPrediction |
| isorhamnetin | KDR | SwissTargetPrediction |
| isorhamnetin | MMP13 | SwissTargetPrediction |
| isorhamnetin | MMP3 | SwissTargetPrediction |
| isorhamnetin | CA3 | SwissTargetPrediction |
| isorhamnetin | PLK1 | SwissTargetPrediction |
| isorhamnetin | CA6 | SwissTargetPrediction |
| isorhamnetin | CDK1 | SwissTargetPrediction |
| isorhamnetin | MMP9 | SwissTargetPrediction |
| isorhamnetin | MMP2 | SwissTargetPrediction |
| isorhamnetin | PKN1 | SwissTargetPrediction |
| isorhamnetin | CA14 | SwissTargetPrediction |
| isorhamnetin | CA9 | SwissTargetPrediction |
| isorhamnetin | CSNK2A1 | SwissTargetPrediction |
| isorhamnetin | MET | SwissTargetPrediction |
| isorhamnetin | NEK2 | SwissTargetPrediction |
| isorhamnetin | CXCR1 | SwissTargetPrediction |
| isorhamnetin | CAMK2B | SwissTargetPrediction |
| isorhamnetin | ALK | SwissTargetPrediction |
| isorhamnetin | AKT1 | SwissTargetPrediction |
| isorhamnetin | ABCB1 | SwissTargetPrediction |
| isorhamnetin | NEK6 | SwissTargetPrediction |
| isorhamnetin | PLA2G1B | SwissTargetPrediction |
| isorhamnetin | CA5A | SwissTargetPrediction |
| isorhamnetin | BACE1 | SwissTargetPrediction |
| isorhamnetin | AXL | SwissTargetPrediction |
| isorhamnetin | NUAK1 | SwissTargetPrediction |
| isorhamnetin | AKR1C2 | SwissTargetPrediction |
| isorhamnetin | AKR1C1 | SwissTargetPrediction |
| isorhamnetin | AKR1C3 | SwissTargetPrediction |
| isorhamnetin | AKR1C4 | SwissTargetPrediction |
| isorhamnetin | CA13 | SwissTargetPrediction |
| isorhamnetin | AKR1A1 | SwissTargetPrediction |
| isorhamnetin | GPR35 | SwissTargetPrediction |
| isorhamnetin | MAPT | SwissTargetPrediction |
| isorhamnetin | KDM4E | SwissTargetPrediction |
| isorhamnetin | TOP2A | SwissTargetPrediction |
| isorhamnetin | INSR | SwissTargetPrediction |
| isorhamnetin | MYLK | SwissTargetPrediction |
| isorhamnetin | SYK | SwissTargetPrediction |
| isorhamnetin | PIK3CG | SwissTargetPrediction |
| isorhamnetin | APEX1 | SwissTargetPrediction |
| isorhamnetin | CDK5R1 CDK5 | SwissTargetPrediction |
| isorhamnetin | CCNB3 CDK1 CCNB1 CCNB2 | SwissTargetPrediction |
| isorhamnetin | CDK6 | SwissTargetPrediction |
| isorhamnetin | CDK2 | SwissTargetPrediction |
| isorhamnetin | ARG1 | SwissTargetPrediction |
| isorhamnetin | APP | SwissTargetPrediction |
| isorhamnetin | MCL1 | SwissTargetPrediction |
| isorhamnetin | TERT | SwissTargetPrediction |
| isorhamnetin | TYR | SwissTargetPrediction |
| isorhamnetin | HSD17B1 | SwissTargetPrediction |
| isorhamnetin | AHR | SwissTargetPrediction |
| isorhamnetin | ESRRA | SwissTargetPrediction |
| isorhamnetin | PTPRS | SwissTargetPrediction |
| isorhamnetin | PLG | SwissTargetPrediction |
| isorhamnetin | ESR2 | SwissTargetPrediction |
| isorhamnetin | MPG | SwissTargetPrediction |
| isorhamnetin | SLC22A12 | SwissTargetPrediction |
| isorhamnetin | PARP1 | SwissTargetPrediction |
| isorhamnetin | TTR | SwissTargetPrediction |
| isorhamnetin | MMP12 | SwissTargetPrediction |
| isorhamnetin | CD38 | SwissTargetPrediction |
| isorhamnetin | AKR1B10 | SwissTargetPrediction |
| isorhamnetin | TNKS2 | SwissTargetPrediction |
| isorhamnetin | NOS2 | SwissTargetPrediction |
| isorhamnetin | PTGS1 | SwissTargetPrediction |
| isorhamnetin | ESR1 | SwissTargetPrediction |
| isorhamnetin | AR | SwissTargetPrediction |
| isorhamnetin | PPARG | SwissTargetPrediction |
| isorhamnetin | PTGS2 | SwissTargetPrediction |
| isorhamnetin | MAPK14 | SwissTargetPrediction |
| isorhamnetin | PRSS1 | SwissTargetPrediction |
| isorhamnetin | CCNA2 | SwissTargetPrediction |
| isorhamnetin | NCOA2 | SwissTargetPrediction |
| isorhamnetin | PYGM | SwissTargetPrediction |
| isorhamnetin | PPARD | SwissTargetPrediction |
| isorhamnetin | CHEK1 | SwissTargetPrediction |
| isorhamnetin | NCOA1 | SwissTargetPrediction |
| isorhamnetin | F7 | SwissTargetPrediction |
| isorhamnetin | GABRA1 | SwissTargetPrediction |
| isorhamnetin | MAOB | SwissTargetPrediction |
| isorhamnetin | GRIA2 | SwissTargetPrediction |
| isorhamnetin | RELA | SwissTargetPrediction |
| isorhamnetin | NCF1 | SwissTargetPrediction |
| isorhamnetin | OLR1 | SwissTargetPrediction |
| baohuoside ii / ikarisoside a | PDE5A | SwissTargetPrediction |
| baohuoside ii / ikarisoside a | CA7 | SwissTargetPrediction |
| baohuoside ii / ikarisoside a | CA12 | SwissTargetPrediction |
| baohuoside ii / ikarisoside a | CA4 | SwissTargetPrediction |
| baohuoside ii / ikarisoside a | AKR1B1 | SwissTargetPrediction |
| baohuoside ii / ikarisoside a | RPS6KA3 | SwissTargetPrediction |
| baohuoside ii / ikarisoside a | CA2 | SwissTargetPrediction |
| baohuoside ii / ikarisoside a | NOX4 | SwissTargetPrediction |
| baohuoside ii / ikarisoside a | ACHE | SwissTargetPrediction |
| baohuoside ii / ikarisoside a | NQO2 | SwissTargetPrediction |
| baohuoside ii / ikarisoside a | PTGS2 | TCMSP |
| baohuoside ii / ikarisoside a | ADRA2C | SwissTargetPrediction |
| baohuoside ii / ikarisoside a | NMUR2 | SwissTargetPrediction |
| baohuoside ii / ikarisoside a | ADRA2A | SwissTargetPrediction |
| baohuoside ii / ikarisoside a | CD38 | SwissTargetPrediction |
| baohuoside ii / ikarisoside a | KLK1 | SwissTargetPrediction |
| baohuoside ii / ikarisoside a | KLK2 | SwissTargetPrediction |
| baohuoside ii / ikarisoside a | PRKCG | SwissTargetPrediction |
| baohuoside ii / ikarisoside a | PRKCD | SwissTargetPrediction |
| baohuoside ii / ikarisoside a | PRKCA | SwissTargetPrediction |
| baohuoside ii / ikarisoside a | PRKCB | SwissTargetPrediction |
| baohuoside ii / ikarisoside a | PRKCE | SwissTargetPrediction |
| baohuoside ii / ikarisoside a | PRKCH | SwissTargetPrediction |
| baohuoside ii / ikarisoside a | ADORA1 | SwissTargetPrediction |
| baohuoside ii / ikarisoside a | CHEK2 | SwissTargetPrediction |
| baohuoside ii / ikarisoside a | CHEK1 | SwissTargetPrediction |
| baohuoside ii / ikarisoside a | HSP90AB1 | SwissTargetPrediction |
| baohuoside ii / ikarisoside a | IL2 | SwissTargetPrediction |
| baohuoside ii / ikarisoside a | IDH1 | SwissTargetPrediction |
| baohuoside ii / ikarisoside a | PTGS1 | SwissTargetPrediction |
| baohuoside ii / ikarisoside a | FASN | SwissTargetPrediction |
| baohuoside ii / ikarisoside a | DUSP3 | SwissTargetPrediction |
| baohuoside ii / ikarisoside a | KISS1R | SwissTargetPrediction |
| baohuoside ii / ikarisoside a | CTNNB1 | SwissTargetPrediction |
| baohuoside ii / ikarisoside a | ALOX5 | SwissTargetPrediction |
| baohuoside ii / ikarisoside a | PTPN1 | SwissTargetPrediction |
| baohuoside ii / ikarisoside a | TNNC1 TNNT2 TNNI3 | SwissTargetPrediction |
| baohuoside ii / ikarisoside a | F10 | SwissTargetPrediction |
| baohuoside ii / ikarisoside a | SERPINE1 | SwissTargetPrediction |
| baohuoside ii / ikarisoside a | KCNH2 | SwissTargetPrediction |
| baohuoside ii / ikarisoside a | CCR1 | SwissTargetPrediction |
| baohuoside ii / ikarisoside a | PRKCZ | SwissTargetPrediction |
| baohuoside ii / ikarisoside a | IKBKB | SwissTargetPrediction |
| baohuoside ii / ikarisoside a | ITGB1 ITGA4 | SwissTargetPrediction |
| baohuoside ii / ikarisoside a | PDK1 | SwissTargetPrediction |
| baohuoside ii / ikarisoside a | HSP90AA1 | SwissTargetPrediction |
| baohuoside ii / ikarisoside a | PRKACA | SwissTargetPrediction |
| baohuoside ii / ikarisoside a | TNF | SwissTargetPrediction |
| baohuoside ii / ikarisoside a | SQLE | SwissTargetPrediction |
| baohuoside ii / ikarisoside a | ADRB1 | SwissTargetPrediction |
| baohuoside ii / ikarisoside a | VCP | SwissTargetPrediction |
| baohuoside ii / ikarisoside a | ESR1 | TCMSP |
| baohuoside ii / ikarisoside a | MELK | SwissTargetPrediction |
| baohuoside ii / ikarisoside a | BCL2L1 | SwissTargetPrediction |
| baohuoside ii / ikarisoside a | LDHA | SwissTargetPrediction |
| baohuoside ii / ikarisoside a | BCL2 | SwissTargetPrediction |
| baohuoside ii / ikarisoside a | LDHB | SwissTargetPrediction |
| baohuoside ii / ikarisoside a | XDH | SwissTargetPrediction |
| baohuoside ii / ikarisoside a | CDK1 CCNB1 | SwissTargetPrediction |
| baohuoside ii / ikarisoside a | CYP19A1 | SwissTargetPrediction |
| baohuoside ii / ikarisoside a | GCGR | SwissTargetPrediction |
| baohuoside ii / ikarisoside a | PSMG3 | SwissTargetPrediction |
| baohuoside ii / ikarisoside a | WEE1 | SwissTargetPrediction |
| baohuoside ii / ikarisoside a | SLC29A1 | SwissTargetPrediction |
| baohuoside ii / ikarisoside a | KDM2A | SwissTargetPrediction |
| baohuoside ii / ikarisoside a | KDM6B | SwissTargetPrediction |
| baohuoside ii / ikarisoside a | DHODH | SwissTargetPrediction |
| baohuoside ii / ikarisoside a | TP53 | SwissTargetPrediction |
| baohuoside ii / ikarisoside a | RAF1 | SwissTargetPrediction |
| baohuoside ii / ikarisoside a | MTOR | SwissTargetPrediction |
| baohuoside ii / ikarisoside a | PIK3CA | SwissTargetPrediction |
| baohuoside ii / ikarisoside a | LCK | SwissTargetPrediction |
| baohuoside ii / ikarisoside a | FBP1 | SwissTargetPrediction |
| baohuoside ii / ikarisoside a | ERN1 | SwissTargetPrediction |
| baohuoside ii / ikarisoside a | GRK6 | SwissTargetPrediction |
| baohuoside ii / ikarisoside a | TRAP1 | SwissTargetPrediction |
| baohuoside ii / ikarisoside a | CCR4 | SwissTargetPrediction |
| baohuoside ii / ikarisoside a | AGTR1 | SwissTargetPrediction |
| baohuoside ii / ikarisoside a | F7 | TCMSP |
| baohuoside ii / ikarisoside a | TDP1 | SwissTargetPrediction |
| baohuoside ii / ikarisoside a | FKBP1A | SwissTargetPrediction |
| baohuoside ii / ikarisoside a | FKBP5 | SwissTargetPrediction |
| baohuoside ii / ikarisoside a | ADRB2 | SwissTargetPrediction |
| baohuoside ii / ikarisoside a | ADRA1D | SwissTargetPrediction |
| baohuoside ii / ikarisoside a | PTGER3 | SwissTargetPrediction |
| baohuoside ii / ikarisoside a | HSP90B1 | SwissTargetPrediction |
| baohuoside ii / ikarisoside a | CNOT7 | SwissTargetPrediction |
| baohuoside ii / ikarisoside a | ADCYAP1R1 | SwissTargetPrediction |
| baohuoside ii / ikarisoside a | HDAC1 | SwissTargetPrediction |
| baohuoside ii / ikarisoside a | ABCB1 | SwissTargetPrediction |
| baohuoside ii / ikarisoside a | KLKB1 | SwissTargetPrediction |
| baohuoside ii / ikarisoside a | SIRT1 | SwissTargetPrediction |
| baohuoside ii / ikarisoside a | ABCG2 | SwissTargetPrediction |
| baohuoside ii / ikarisoside a | ADORA3 | SwissTargetPrediction |
| baohuoside ii / ikarisoside a | GRK7 | SwissTargetPrediction |
| baohuoside ii / ikarisoside a | GRK3 | SwissTargetPrediction |
| baohuoside ii / ikarisoside a | GRK2 | SwissTargetPrediction |
| baohuoside ii / ikarisoside a | GRK1 | SwissTargetPrediction |
| baohuoside ii / ikarisoside a | GRK5 | SwissTargetPrediction |
| baohuoside ii / ikarisoside a | GRK4 | SwissTargetPrediction |
| baohuoside ii / ikarisoside a | NOS2 | TCMSP |
| baohuoside ii / ikarisoside a | AR | TCMSP |
| baohuoside ii / ikarisoside a | SCN5A | TCMSP |
| baohuoside ii / ikarisoside a | PPARG | TCMSP |
| baohuoside ii / ikarisoside a | KDR | TCMSP |
| baohuoside ii / ikarisoside a | MAPK14 | TCMSP |
| baohuoside ii / ikarisoside a | GSK3B | TCMSP |
| baohuoside ii / ikarisoside a | PRSS1 | TCMSP |
| baohuoside ii / ikarisoside a | CCNA2 | TCMSP |
| baohuoside ii / ikarisoside a | NCOA2 | TCMSP |
| tripterifordin | CDC25A | SwissTargetPrediction |
| tripterifordin | PTPN1 | SwissTargetPrediction |
| tripterifordin | CYP19A1 | SwissTargetPrediction |
| tripterifordin | HMGCR | SwissTargetPrediction |
| tripterifordin | HSD11B1 | SwissTargetPrediction |
| tripterifordin | VDR | SwissTargetPrediction |
| tripterifordin | HSD17B3 | SwissTargetPrediction |
| tripterifordin | UGT2B7 | SwissTargetPrediction |
| tripterifordin | SIRT2 | SwissTargetPrediction |
| tripterifordin | ACHE | SwissTargetPrediction |
| tripterifordin | TRPV1 | SwissTargetPrediction |
| tripterifordin | OPRK1 | SwissTargetPrediction |
| tripterifordin | AR | SwissTargetPrediction |
| tripterifordin | SRD5A1 | SwissTargetPrediction |
| tripterifordin | CA1 | SwissTargetPrediction |
| tripterifordin | CA9 | SwissTargetPrediction |
| tripterifordin | CCR1 | SwissTargetPrediction |
| tripterifordin | HIF1A | SwissTargetPrediction |
| tripterifordin | SHBG | SwissTargetPrediction |
| tripterifordin | C5AR1 | SwissTargetPrediction |
| tripterifordin | CYP17A1 | SwissTargetPrediction |
| tripterifordin | PRKCA | SwissTargetPrediction |
| tripterifordin | APP | SwissTargetPrediction |
| tripterifordin | BACE1 | SwissTargetPrediction |
| tripterifordin | SERPINA6 | SwissTargetPrediction |
| tripterifordin | PRSS1 | SwissTargetPrediction |
| tripterifordin | CTRC | SwissTargetPrediction |
| tripterifordin | LIPA | SwissTargetPrediction |
| tripterifordin | SLC18A3 | SwissTargetPrediction |
| tripterifordin | NR1I3 | SwissTargetPrediction |
| tripterifordin | PSEN2 PSENEN NCSTN APH1A PSEN1 APH1B | SwissTargetPrediction |
| tripterifordin | RHOA | SwissTargetPrediction |
| tripterifordin | EPHX2 | SwissTargetPrediction |
| tripterifordin | NR3C1 | TCMSP |
| tripterifordin | NR3C2 | TCMSP |
| tripterifordin | PGR | SwissTargetPrediction |
| tripterifordin | CA2 | SwissTargetPrediction |
| tripterifordin | TRPV4 | SwissTargetPrediction |
| tripterifordin | BRD4 | SwissTargetPrediction |
| tripterifordin | PABPC1 | SwissTargetPrediction |
| tripterifordin | IDO1 | SwissTargetPrediction |
| tripterifordin | PRCP | SwissTargetPrediction |
| tripterifordin | SLC6A9 | SwissTargetPrediction |
| tripterifordin | GABBR2 GABBR1 | SwissTargetPrediction |
| tripterifordin | PREP | SwissTargetPrediction |
| tripterifordin | PYGL | SwissTargetPrediction |
| tripterifordin | CTSS | SwissTargetPrediction |
| tripterifordin | PRKCB | SwissTargetPrediction |
| tripterifordin | RASGRP1 | SwissTargetPrediction |
| tripterifordin | PPM1B | SwissTargetPrediction |
| tripterifordin | PPP1CC | SwissTargetPrediction |
| tripterifordin | ADORA3 | SwissTargetPrediction |
| tripterifordin | MAPK3 | SwissTargetPrediction |
| tripterifordin | OPRM1 | SwissTargetPrediction |
| tripterifordin | PTGS1 | SwissTargetPrediction |
| tripterifordin | PPP5C | SwissTargetPrediction |
| tripterifordin | PPP1CA | SwissTargetPrediction |
| tripterifordin | PPARG | SwissTargetPrediction |
| tripterifordin | P2RX7 | SwissTargetPrediction |
| tripterifordin | MDM2 | SwissTargetPrediction |
| tripterifordin | CDC25B | SwissTargetPrediction |
| tripterifordin | RAPGEF4 | SwissTargetPrediction |
| tripterifordin | GCGR | SwissTargetPrediction |
| tripterifordin | PIK3CB | SwissTargetPrediction |
| tripterifordin | MMP3 | SwissTargetPrediction |
| tripterifordin | MMP9 | SwissTargetPrediction |
| tripterifordin | MMP1 | SwissTargetPrediction |
| tripterifordin | PTGES | SwissTargetPrediction |
| tripterifordin | SCN9A | SwissTargetPrediction |
| tripterifordin | HDAC6 | SwissTargetPrediction |
| tripterifordin | CCNB3 CDK1 CCNB1 CCNB2 | SwissTargetPrediction |
| tripterifordin | CDK1 | SwissTargetPrediction |
| tripterifordin | PRKCG | SwissTargetPrediction |
| tripterifordin | PRKCE | SwissTargetPrediction |
| tripterifordin | PRKCH | SwissTargetPrediction |
| tripterifordin | PRKCQ | SwissTargetPrediction |
| tripterifordin | PTGS2 | SwissTargetPrediction |
| tripterifordin | MTNR1A | SwissTargetPrediction |
| tripterifordin | MTNR1B | SwissTargetPrediction |
| tripterifordin | UGCG | SwissTargetPrediction |
| tripterifordin | FASN | SwissTargetPrediction |
| tripterifordin | KCNQ2 | SwissTargetPrediction |
| tripterifordin | MMP13 | SwissTargetPrediction |
| tripterifordin | PAM | SwissTargetPrediction |
| tripterifordin | CDK5R1 CDK5 | SwissTargetPrediction |
| tripterifordin | MAPK14 | SwissTargetPrediction |
| tripterifordin | KCNQ1 | SwissTargetPrediction |
| tripterifordin | OPRL1 | SwissTargetPrediction |
| tripterifordin | OPRD1 | SwissTargetPrediction |
| tripterifordin | JAK2 | SwissTargetPrediction |
| tripterifordin | FDFT1 | SwissTargetPrediction |
| tripterifordin | AKR1C3 | SwissTargetPrediction |
| tripterifordin | SLC10A2 | SwissTargetPrediction |
| tripterifordin | PKM | SwissTargetPrediction |
| tripterifordin | PIK3CD | SwissTargetPrediction |
| tripterifordin | CALCRL | SwissTargetPrediction |
| tripterifordin | PIK3CA | SwissTargetPrediction |
| tripterifordin | PRMT3 | SwissTargetPrediction |
| tripterifordin | HSD17B7 | SwissTargetPrediction |
| tripterifordin | HSP90B1 | SwissTargetPrediction |
| sagittatoside b | PDE5A | SwissTargetPrediction |
| sagittatoside b | CA7 | SwissTargetPrediction |
| sagittatoside b | CA12 | SwissTargetPrediction |
| sagittatoside b | CA4 | SwissTargetPrediction |
| sagittatoside b | RPS6KA3 | SwissTargetPrediction |
| sagittatoside b | AKR1B1 | SwissTargetPrediction |
| sagittatoside b | NQO2 | SwissTargetPrediction |
| sagittatoside b | CA2 | SwissTargetPrediction |
| sagittatoside b | NOX4 | SwissTargetPrediction |
| sagittatoside b | NMUR2 | SwissTargetPrediction |
| sagittatoside b | ADRA2A | SwissTargetPrediction |
| sagittatoside b | ADRA2C | SwissTargetPrediction |
| sagittatoside b | ACHE | SwissTargetPrediction |
| sagittatoside b | PTGS2 | SwissTargetPrediction |
| sagittatoside b | CD38 | SwissTargetPrediction |
| sagittatoside b | SLC5A2 | SwissTargetPrediction |
| sagittatoside b | IL2 | SwissTargetPrediction |
| sagittatoside b | XDH | SwissTargetPrediction |
| sagittatoside b | SLC5A1 | SwissTargetPrediction |
| sagittatoside b | TRAP1 | SwissTargetPrediction |
| sagittatoside b | HSP90AA1 | SwissTargetPrediction |
| sagittatoside b | HSP90AB1 | SwissTargetPrediction |
| sagittatoside b | BCL2 | SwissTargetPrediction |
| sagittatoside b | TDP1 | SwissTargetPrediction |
| sagittatoside b | ADORA1 | SwissTargetPrediction |
| sagittatoside b | ALOX5 | SwissTargetPrediction |
| sagittatoside b | CA14 | SwissTargetPrediction |
| sagittatoside b | PRKCA | SwissTargetPrediction |
| sagittatoside b | PRKCB | SwissTargetPrediction |
| sagittatoside b | PRKCE | SwissTargetPrediction |
| sagittatoside b | ITGA3 | SwissTargetPrediction |
| sagittatoside b | GCGR | SwissTargetPrediction |
| sagittatoside b | SQLE | SwissTargetPrediction |
| sagittatoside b | PRKCD | SwissTargetPrediction |
| sagittatoside b | SERPINE1 | SwissTargetPrediction |
| sagittatoside b | KISS1R | SwissTargetPrediction |
| sagittatoside b | KDM2A | SwissTargetPrediction |
| sagittatoside b | KDM6B | SwissTargetPrediction |
| sagittatoside b | PTGER3 | SwissTargetPrediction |
| sagittatoside b | F10 | SwissTargetPrediction |
| sagittatoside b | PRKCH | SwissTargetPrediction |
| sagittatoside b | PDK1 | SwissTargetPrediction |
| sagittatoside b | F2 | SwissTargetPrediction |
| sagittatoside b | ABCB1 | SwissTargetPrediction |
| sagittatoside b | AMY1A | SwissTargetPrediction |
| sagittatoside b | PRKCG | SwissTargetPrediction |
| sagittatoside b | CHEK2 | SwissTargetPrediction |
| sagittatoside b | CHEK1 | SwissTargetPrediction |
| sagittatoside b | KLK1 | SwissTargetPrediction |
| sagittatoside b | KLK2 | SwissTargetPrediction |
| sagittatoside b | PTPN2 | SwissTargetPrediction |
| sagittatoside b | NOS2 | SwissTargetPrediction |
| sagittatoside b | BCL2L1 | SwissTargetPrediction |
| sagittatoside b | ADRA1D | SwissTargetPrediction |
| sagittatoside b | ADRA1A | SwissTargetPrediction |
| sagittatoside b | ADRA1B | SwissTargetPrediction |
| sagittatoside b | KCNH2 | SwissTargetPrediction |
| sagittatoside b | CCR1 | SwissTargetPrediction |
| sagittatoside b | DNM2 | SwissTargetPrediction |
| sagittatoside b | TERT | SwissTargetPrediction |
| sagittatoside b | HDAC1 | SwissTargetPrediction |
| sagittatoside b | ITGB1 ITGA4 | SwissTargetPrediction |
| sagittatoside b | ABCB11 | SwissTargetPrediction |
| sagittatoside b | PTPRA | SwissTargetPrediction |
| sagittatoside b | LCK | SwissTargetPrediction |
| sagittatoside b | ABCG2 | SwissTargetPrediction |
| sagittatoside b | ADRB2 | SwissTargetPrediction |
| sagittatoside b | TP53 | SwissTargetPrediction |
| sagittatoside b | TNF | SwissTargetPrediction |
| sagittatoside b | MTOR | SwissTargetPrediction |
| sagittatoside b | PIK3CA | SwissTargetPrediction |
| sagittatoside b | VCP | SwissTargetPrediction |
| sagittatoside b | ESR1 | SwissTargetPrediction |
| sagittatoside b | MPG | SwissTargetPrediction |
| sagittatoside b | PRKACA | SwissTargetPrediction |
| sagittatoside b | LDHA | SwissTargetPrediction |
| sagittatoside b | LDHB | SwissTargetPrediction |
| sagittatoside b | CYP1B1 | SwissTargetPrediction |
| sagittatoside b | TNNC1 TNNT2 TNNI3 | SwissTargetPrediction |
| sagittatoside b | MELK | SwissTargetPrediction |
| sagittatoside b | ADRB1 | SwissTargetPrediction |
| sagittatoside b | KDM1A | SwissTargetPrediction |
| sagittatoside b | ALDH2 | SwissTargetPrediction |
| sagittatoside b | KCNA3 | SwissTargetPrediction |
| sagittatoside b | BCHE | SwissTargetPrediction |
| sagittatoside b | PTPN1 | SwissTargetPrediction |
| sagittatoside b | PLG | SwissTargetPrediction |
| sagittatoside b | CA13 | SwissTargetPrediction |
| sagittatoside b | EGFR | SwissTargetPrediction |
| sagittatoside b | CYP19A1 | SwissTargetPrediction |
| sagittatoside b | ADORA3 | SwissTargetPrediction |
| sagittatoside b | SRC | SwissTargetPrediction |
| sagittatoside b | APP | SwissTargetPrediction |
| sagittatoside b | SIGMAR1 | SwissTargetPrediction |
| sagittatoside b | BACE1 | SwissTargetPrediction |
| sagittatoside b | CA1 | SwissTargetPrediction |
| sagittatoside b | CA9 | SwissTargetPrediction |
| sagittatoside b | NFKB1 | SwissTargetPrediction |
| sagittatoside b | CYP1A1 | SwissTargetPrediction |
| sagittatoside b | CYP1A2 | SwissTargetPrediction |
| 2″-o-rhamnosylicariside ii/rha-icariside | PDE5A | SwissTargetPrediction |
| 2″-o-rhamnosylicariside ii/rha-icariside | AKR1B1 | SwissTargetPrediction |
| 2″-o-rhamnosylicariside ii/rha-icariside | NQO2 | SwissTargetPrediction |
| 2″-o-rhamnosylicariside ii/rha-icariside | RPS6KA3 | SwissTargetPrediction |
| 2″-o-rhamnosylicariside ii/rha-icariside | CA7 | SwissTargetPrediction |
| 2″-o-rhamnosylicariside ii/rha-icariside | CA12 | SwissTargetPrediction |
| 2″-o-rhamnosylicariside ii/rha-icariside | CA4 | SwissTargetPrediction |
| 2″-o-rhamnosylicariside ii/rha-icariside | NOX4 | SwissTargetPrediction |
| 2″-o-rhamnosylicariside ii/rha-icariside | CA2 | SwissTargetPrediction |
| 2″-o-rhamnosylicariside ii/rha-icariside | NMUR2 | SwissTargetPrediction |
| 2″-o-rhamnosylicariside ii/rha-icariside | ADRA2A | SwissTargetPrediction |
| 2″-o-rhamnosylicariside ii/rha-icariside | ADRA2C | SwissTargetPrediction |
| 2″-o-rhamnosylicariside ii/rha-icariside | ACHE | SwissTargetPrediction |
| 2″-o-rhamnosylicariside ii/rha-icariside | PTGS2 | SwissTargetPrediction |
| 2″-o-rhamnosylicariside ii/rha-icariside | CD38 | SwissTargetPrediction |
| 2″-o-rhamnosylicariside ii/rha-icariside | SLC5A2 | SwissTargetPrediction |
| 2″-o-rhamnosylicariside ii/rha-icariside | IL2 | SwissTargetPrediction |
| 2″-o-rhamnosylicariside ii/rha-icariside | ITGA3 | SwissTargetPrediction |
| 2″-o-rhamnosylicariside ii/rha-icariside | PRKCE | SwissTargetPrediction |
| 2″-o-rhamnosylicariside ii/rha-icariside | PTGER3 | SwissTargetPrediction |
| 2″-o-rhamnosylicariside ii/rha-icariside | BCL2 | SwissTargetPrediction |
| 2″-o-rhamnosylicariside ii/rha-icariside | ALOX5 | SwissTargetPrediction |
| 2″-o-rhamnosylicariside ii/rha-icariside | NOS2 | SwissTargetPrediction |
| 2″-o-rhamnosylicariside ii/rha-icariside | ADORA1 | SwissTargetPrediction |
| 2″-o-rhamnosylicariside ii/rha-icariside | XDH | SwissTargetPrediction |
| 2″-o-rhamnosylicariside ii/rha-icariside | PRKCD | SwissTargetPrediction |
| 2″-o-rhamnosylicariside ii/rha-icariside | PRKCA | SwissTargetPrediction |
| 2″-o-rhamnosylicariside ii/rha-icariside | PRKCB | SwissTargetPrediction |
| 2″-o-rhamnosylicariside ii/rha-icariside | CA14 | SwissTargetPrediction |
| 2″-o-rhamnosylicariside ii/rha-icariside | PRKCH | SwissTargetPrediction |
| 2″-o-rhamnosylicariside ii/rha-icariside | ABCB1 | SwissTargetPrediction |
| 2″-o-rhamnosylicariside ii/rha-icariside | TRAP1 | SwissTargetPrediction |
| 2″-o-rhamnosylicariside ii/rha-icariside | TDP1 | SwissTargetPrediction |
| 2″-o-rhamnosylicariside ii/rha-icariside | HSP90AA1 | SwissTargetPrediction |
| 2″-o-rhamnosylicariside ii/rha-icariside | PTPRA | SwissTargetPrediction |
| 2″-o-rhamnosylicariside ii/rha-icariside | HSP90AB1 | SwissTargetPrediction |
| 2″-o-rhamnosylicariside ii/rha-icariside | KISS1R | SwissTargetPrediction |
| 2″-o-rhamnosylicariside ii/rha-icariside | PDK1 | SwissTargetPrediction |
| 2″-o-rhamnosylicariside ii/rha-icariside | F2 | SwissTargetPrediction |
| 2″-o-rhamnosylicariside ii/rha-icariside | F10 | SwissTargetPrediction |
| 2″-o-rhamnosylicariside ii/rha-icariside | KDM1A | SwissTargetPrediction |
| 2″-o-rhamnosylicariside ii/rha-icariside | SERPINE1 | SwissTargetPrediction |
| 2″-o-rhamnosylicariside ii/rha-icariside | SQLE | SwissTargetPrediction |
| 2″-o-rhamnosylicariside ii/rha-icariside | PRKCG | SwissTargetPrediction |
| 2″-o-rhamnosylicariside ii/rha-icariside | SLC5A1 | SwissTargetPrediction |
| 2″-o-rhamnosylicariside ii/rha-icariside | AMY1A | SwissTargetPrediction |
| 2″-o-rhamnosylicariside ii/rha-icariside | KDM2A | SwissTargetPrediction |
| 2″-o-rhamnosylicariside ii/rha-icariside | KDM6B | SwissTargetPrediction |
| 2″-o-rhamnosylicariside ii/rha-icariside | HDAC1 | SwissTargetPrediction |
| 2″-o-rhamnosylicariside ii/rha-icariside | BCL2L1 | SwissTargetPrediction |
| 2″-o-rhamnosylicariside ii/rha-icariside | FKBP1A | SwissTargetPrediction |
| 2″-o-rhamnosylicariside ii/rha-icariside | FKBP5 | SwissTargetPrediction |
| 2″-o-rhamnosylicariside ii/rha-icariside | GCGR | SwissTargetPrediction |
| 2″-o-rhamnosylicariside ii/rha-icariside | KCNH2 | SwissTargetPrediction |
| 2″-o-rhamnosylicariside ii/rha-icariside | CCR1 | SwissTargetPrediction |
| 2″-o-rhamnosylicariside ii/rha-icariside | TERT | SwissTargetPrediction |
| 2″-o-rhamnosylicariside ii/rha-icariside | ABCG2 | SwissTargetPrediction |
| 2″-o-rhamnosylicariside ii/rha-icariside | CTSB | SwissTargetPrediction |
| 2″-o-rhamnosylicariside ii/rha-icariside | AKT1 | SwissTargetPrediction |
| 2″-o-rhamnosylicariside ii/rha-icariside | TNF | SwissTargetPrediction |
| 2″-o-rhamnosylicariside ii/rha-icariside | DNM2 | SwissTargetPrediction |
| 2″-o-rhamnosylicariside ii/rha-icariside | CYP1B1 | SwissTargetPrediction |
| 2″-o-rhamnosylicariside ii/rha-icariside | KLKB1 | SwissTargetPrediction |
| 2″-o-rhamnosylicariside ii/rha-icariside | F11 | SwissTargetPrediction |
| 2″-o-rhamnosylicariside ii/rha-icariside | ALDH2 | SwissTargetPrediction |
| 2″-o-rhamnosylicariside ii/rha-icariside | KCNA3 | SwissTargetPrediction |
| 2″-o-rhamnosylicariside ii/rha-icariside | BCHE | SwissTargetPrediction |
| 2″-o-rhamnosylicariside ii/rha-icariside | PTPN1 | SwissTargetPrediction |
| 2″-o-rhamnosylicariside ii/rha-icariside | CYP19A1 | SwissTargetPrediction |
| 2″-o-rhamnosylicariside ii/rha-icariside | PLG | SwissTargetPrediction |
| 2″-o-rhamnosylicariside ii/rha-icariside | MCL1 | SwissTargetPrediction |
| 2″-o-rhamnosylicariside ii/rha-icariside | CA13 | SwissTargetPrediction |
| 2″-o-rhamnosylicariside ii/rha-icariside | EGFR | SwissTargetPrediction |
| 2″-o-rhamnosylicariside ii/rha-icariside | ADORA3 | SwissTargetPrediction |
| 2″-o-rhamnosylicariside ii/rha-icariside | APP | SwissTargetPrediction |
| 2″-o-rhamnosylicariside ii/rha-icariside | RELA | SwissTargetPrediction |
| 2″-o-rhamnosylicariside ii/rha-icariside | SRC | SwissTargetPrediction |
| 2″-o-rhamnosylicariside ii/rha-icariside | PPARG | SwissTargetPrediction |
| 2″-o-rhamnosylicariside ii/rha-icariside | SIGMAR1 | SwissTargetPrediction |
| 2″-o-rhamnosylicariside ii/rha-icariside | DRD2 | SwissTargetPrediction |
| 2″-o-rhamnosylicariside ii/rha-icariside | CA1 | SwissTargetPrediction |
| 2″-o-rhamnosylicariside ii/rha-icariside | CA9 | SwissTargetPrediction |
| 2″-o-rhamnosylicariside ii/rha-icariside | NFKB1 | SwissTargetPrediction |
| 2″-o-rhamnosylicariside ii/rha-icariside | CYP1A1 | SwissTargetPrediction |
| 2″-o-rhamnosylicariside ii/rha-icariside | CYP1A2 | SwissTargetPrediction |
| 2″-o-rhamnosylicariside ii/rha-icariside | PLA2G1B | SwissTargetPrediction |
| 2″-o-rhamnosylicariside ii/rha-icariside | SOAT1 | SwissTargetPrediction |
| 2″-o-rhamnosylicariside ii/rha-icariside | SOAT2 | SwissTargetPrediction |
| 2″-o-rhamnosylicariside ii/rha-icariside | BACE1 | SwissTargetPrediction |
| 2″-o-rhamnosylicariside ii/rha-icariside | ADORA2A | SwissTargetPrediction |
| 2″-o-rhamnosylicariside ii/rha-icariside | ABCC1 | SwissTargetPrediction |
| 2″-o-rhamnosylicariside ii/rha-icariside | OPRD1 | SwissTargetPrediction |
| 2″-o-rhamnosylicariside ii/rha-icariside | OPRM1 | SwissTargetPrediction |
| icaritin | PDE5A | SwissTargetPrediction |
| icaritin | ABCB1 | SwissTargetPrediction |
| icaritin | CYP19A1 | SwissTargetPrediction |
| icaritin | ABCG2 | SwissTargetPrediction |
| icaritin | PTPN1 | SwissTargetPrediction |
| icaritin | AKT1 | SwissTargetPrediction |
| icaritin | ALOX15 | SwissTargetPrediction |
| icaritin | MCL1 | SwissTargetPrediction |
| icaritin | HSP90AA1 | SwissTargetPrediction |
| icaritin | CYP1B1 | SwissTargetPrediction |
| icaritin | BACE1 | SwissTargetPrediction |
| icaritin | CDK1 | SwissTargetPrediction |
| icaritin | HSP90AB1 | SwissTargetPrediction |
| icaritin | BCHE | SwissTargetPrediction |
| icaritin | ACHE | TCMSP |
| icaritin | PDK1 | SwissTargetPrediction |
| icaritin | HSP90B1 | SwissTargetPrediction |
| icaritin | CDK2 | SwissTargetPrediction |
| icaritin | TYR | SwissTargetPrediction |
| icaritin | ABCC1 | SwissTargetPrediction |
| icaritin | AHR | SwissTargetPrediction |
| icaritin | ESRRA | SwissTargetPrediction |
| icaritin | GSK3B | TCMSP |
| icaritin | ALOX12 | SwissTargetPrediction |
| icaritin | HSD17B2 | SwissTargetPrediction |
| icaritin | HSD17B1 | SwissTargetPrediction |
| icaritin | CA2 | SwissTargetPrediction |
| icaritin | ESR2 | TCMSP |
| icaritin | PTPRS | SwissTargetPrediction |
| icaritin | DAPK1 | SwissTargetPrediction |
| icaritin | MPG | SwissTargetPrediction |
| icaritin | CA12 | SwissTargetPrediction |
| icaritin | PLAU | SwissTargetPrediction |
| icaritin | CDK6 | SwissTargetPrediction |
| icaritin | PDE10A | SwissTargetPrediction |
| icaritin | EGFR | SwissTargetPrediction |
| icaritin | MAPT | SwissTargetPrediction |
| icaritin | KDM4E | SwissTargetPrediction |
| icaritin | TOP2A | SwissTargetPrediction |
| icaritin | MYLK | SwissTargetPrediction |
| icaritin | MPO | SwissTargetPrediction |
| icaritin | PIK3R1 | SwissTargetPrediction |
| icaritin | PYGL | SwissTargetPrediction |
| icaritin | SYK | SwissTargetPrediction |
| icaritin | MMP3 | SwissTargetPrediction |
| icaritin | CA3 | SwissTargetPrediction |
| icaritin | PLK1 | SwissTargetPrediction |
| icaritin | CSNK2A1 | SwissTargetPrediction |
| icaritin | NEK2 | SwissTargetPrediction |
| icaritin | NEK6 | SwissTargetPrediction |
| icaritin | PLA2G1B | SwissTargetPrediction |
| icaritin | CA5A | SwissTargetPrediction |
| icaritin | APEX1 | SwissTargetPrediction |
| icaritin | AKR1C2 | SwissTargetPrediction |
| icaritin | AKR1C1 | SwissTargetPrediction |
| icaritin | AKR1C3 | SwissTargetPrediction |
| icaritin | AKR1C4 | SwissTargetPrediction |
| icaritin | CA13 | SwissTargetPrediction |
| icaritin | PPARG | TCMSP |
| icaritin | CALCA | SwissTargetPrediction |
| icaritin | CDK5R1 CDK5 | SwissTargetPrediction |
| icaritin | GPR84 | SwissTargetPrediction |
| icaritin | CFTR | SwissTargetPrediction |
| icaritin | AKR1B1 | SwissTargetPrediction |
| icaritin | NOS2 | TCMSP |
| icaritin | GCGR | SwissTargetPrediction |
| icaritin | PFKFB3 | SwissTargetPrediction |
| icaritin | FBP1 | SwissTargetPrediction |
| icaritin | DHFR | SwissTargetPrediction |
| icaritin | MELK | SwissTargetPrediction |
| icaritin | NOX4 | SwissTargetPrediction |
| icaritin | MAOA | SwissTargetPrediction |
| icaritin | DRD4 | SwissTargetPrediction |
| icaritin | OPRK1 | SwissTargetPrediction |
| icaritin | EP300 | SwissTargetPrediction |
| icaritin | CNOT7 | SwissTargetPrediction |
| icaritin | ALK | SwissTargetPrediction |
| icaritin | EGLN1 | SwissTargetPrediction |
| icaritin | ADRB2 | TCMSP |
| icaritin | ADRB1 | SwissTargetPrediction |
| icaritin | ERBB2 | SwissTargetPrediction |
| icaritin | FLT3 | SwissTargetPrediction |
| icaritin | PIM1 | SwissTargetPrediction |
| icaritin | CTNNB1 | SwissTargetPrediction |
| icaritin | CA1 | SwissTargetPrediction |
| icaritin | CA9 | SwissTargetPrediction |
| icaritin | CHEK2 | SwissTargetPrediction |
| icaritin | ARG1 | SwissTargetPrediction |
| icaritin | ALOX5 | SwissTargetPrediction |
| icaritin | GABRB3 GABRA3 GABRG2 | SwissTargetPrediction |
| icaritin | GABRB3 GABRG2 GABRA1 | SwissTargetPrediction |
| icaritin | TNKS2 | SwissTargetPrediction |
| icaritin | TNKS | SwissTargetPrediction |
| icaritin | PRKCZ | SwissTargetPrediction |
| icaritin | NAE1 | SwissTargetPrediction |
| icaritin | BCL2 | SwissTargetPrediction |
| icaritin | PTGS1 | TCMSP |
| icaritin | AGTR1 | SwissTargetPrediction |
| icaritin | PTAFR | SwissTargetPrediction |
| icaritin | PTGS2 | TCMSP |
| icaritin | CHRM3 | TCMSP |
| icaritin | KCNH2 | TCMSP |
| icaritin | CHRM1 | TCMSP |
| icaritin | ESR1 | TCMSP |
| icaritin | AR | TCMSP |
| icaritin | SCN5A | TCMSP |
| icaritin | CHRM5 | TCMSP |
| icaritin | RXRA | TCMSP |
| icaritin | ADRA1B | TCMSP |
| icaritin | MAPK14 | TCMSP |
| icaritin | CHEK1 | TCMSP |
| icaritin | RXRB | TCMSP |
| icaritin | PRSS1 | TCMSP |
| icaritin | NCOA2 | TCMSP |
| icaritin | F7 | TCMSP |
| icaritin | KDR | TCMSP |
| icaritin | CCNA2 | TCMSP |
| icaritin | NCOA1 | TCMSP |
| baohuoside i /icarisid | PDE5A | SwissTargetPrediction |
| baohuoside i /icarisid | CA7 | SwissTargetPrediction |
| baohuoside i /icarisid | CA12 | SwissTargetPrediction |
| baohuoside i /icarisid | CA4 | SwissTargetPrediction |
| baohuoside i /icarisid | RPS6KA3 | SwissTargetPrediction |
| baohuoside i /icarisid | CA2 | SwissTargetPrediction |
| baohuoside i /icarisid | AKR1B1 | SwissTargetPrediction |
| baohuoside i /icarisid | NOX4 | SwissTargetPrediction |
| baohuoside i /icarisid | ACHE | SwissTargetPrediction |
| baohuoside i /icarisid | ADRA2C | SwissTargetPrediction |
| baohuoside i /icarisid | NQO2 | SwissTargetPrediction |
| baohuoside i /icarisid | PTGS2 | TCMSP |
| baohuoside i /icarisid | NMUR2 | SwissTargetPrediction |
| baohuoside i /icarisid | ADRA2A | SwissTargetPrediction |
| baohuoside i /icarisid | CD38 | SwissTargetPrediction |
| baohuoside i /icarisid | PRKCE | SwissTargetPrediction |
| baohuoside i /icarisid | CHEK2 | SwissTargetPrediction |
| baohuoside i /icarisid | CHEK1 | SwissTargetPrediction |
| baohuoside i /icarisid | ADORA1 | SwissTargetPrediction |
| baohuoside i /icarisid | PRKCA | SwissTargetPrediction |
| baohuoside i /icarisid | PRKCB | SwissTargetPrediction |
| baohuoside i /icarisid | KLK1 | SwissTargetPrediction |
| baohuoside i /icarisid | KLK2 | SwissTargetPrediction |
| baohuoside i /icarisid | PRKCD | SwissTargetPrediction |
| baohuoside i /icarisid | HSP90AB1 | SwissTargetPrediction |
| baohuoside i /icarisid | TNNC1 TNNT2 TNNI3 | SwissTargetPrediction |
| baohuoside i /icarisid | IL2 | SwissTargetPrediction |
| baohuoside i /icarisid | CTNNB1 | SwissTargetPrediction |
| baohuoside i /icarisid | KCNH2 | SwissTargetPrediction |
| baohuoside i /icarisid | CCR1 | SwissTargetPrediction |
| baohuoside i /icarisid | PRKCG | SwissTargetPrediction |
| baohuoside i /icarisid | PRKCH | SwissTargetPrediction |
| baohuoside i /icarisid | PDK1 | SwissTargetPrediction |
| baohuoside i /icarisid | TNF | SwissTargetPrediction |
| baohuoside i /icarisid | IDH1 | SwissTargetPrediction |
| baohuoside i /icarisid | PTGS1 | SwissTargetPrediction |
| baohuoside i /icarisid | FASN | SwissTargetPrediction |
| baohuoside i /icarisid | F10 | SwissTargetPrediction |
| baohuoside i /icarisid | KISS1R | SwissTargetPrediction |
| baohuoside i /icarisid | HSP90AA1 | SwissTargetPrediction |
| baohuoside i /icarisid | MELK | SwissTargetPrediction |
| baohuoside i /icarisid | VCP | SwissTargetPrediction |
| baohuoside i /icarisid | ESR1 | TCMSP |
| baohuoside i /icarisid | AGTR1 | SwissTargetPrediction |
| baohuoside i /icarisid | BCL2L1 | SwissTargetPrediction |
| baohuoside i /icarisid | LDHA | SwissTargetPrediction |
| baohuoside i /icarisid | BCL2 | SwissTargetPrediction |
| baohuoside i /icarisid | LDHB | SwissTargetPrediction |
| baohuoside i /icarisid | TRAP1 | SwissTargetPrediction |
| baohuoside i /icarisid | XDH | SwissTargetPrediction |
| baohuoside i /icarisid | CDK1 CCNB1 | SwissTargetPrediction |
| baohuoside i /icarisid | SERPINE1 | SwissTargetPrediction |
| baohuoside i /icarisid | MTOR | SwissTargetPrediction |
| baohuoside i /icarisid | PIK3CA | SwissTargetPrediction |
| baohuoside i /icarisid | GCGR | SwissTargetPrediction |
| baohuoside i /icarisid | CYP19A1 | SwissTargetPrediction |
| baohuoside i /icarisid | PTPN1 | SwissTargetPrediction |
| baohuoside i /icarisid | ALOX5 | SwissTargetPrediction |
| baohuoside i /icarisid | ADRB1 | SwissTargetPrediction |
| baohuoside i /icarisid | PTGER3 | SwissTargetPrediction |
| baohuoside i /icarisid | OPRK1 | SwissTargetPrediction |
| baohuoside i /icarisid | IKBKB | SwissTargetPrediction |
| baohuoside i /icarisid | ADRB2 | SwissTargetPrediction |
| baohuoside i /icarisid | ADRA1D | SwissTargetPrediction |
| baohuoside i /icarisid | ITGB1 ITGA4 | SwissTargetPrediction |
| baohuoside i /icarisid | PRKCI | SwissTargetPrediction |
| baohuoside i /icarisid | F7 | TCMSP |
| baohuoside i /icarisid | SQLE | SwissTargetPrediction |
| baohuoside i /icarisid | ADCYAP1R1 | SwissTargetPrediction |
| baohuoside i /icarisid | HSP90B1 | SwissTargetPrediction |
| baohuoside i /icarisid | RAF1 | SwissTargetPrediction |
| baohuoside i /icarisid | KDM2A | SwissTargetPrediction |
| baohuoside i /icarisid | KDM6B | SwissTargetPrediction |
| baohuoside i /icarisid | RARA | SwissTargetPrediction |
| baohuoside i /icarisid | BAD | SwissTargetPrediction |
| baohuoside i /icarisid | FBP1 | SwissTargetPrediction |
| baohuoside i /icarisid | WEE1 | SwissTargetPrediction |
| baohuoside i /icarisid | DHODH | SwissTargetPrediction |
| baohuoside i /icarisid | CCR4 | SwissTargetPrediction |
| baohuoside i /icarisid | PSMG3 | SwissTargetPrediction |
| baohuoside i /icarisid | KLKB1 | SwissTargetPrediction |
| baohuoside i /icarisid | DUSP3 | SwissTargetPrediction |
| baohuoside i /icarisid | PRKACA | SwissTargetPrediction |
| baohuoside i /icarisid | ERN1 | SwissTargetPrediction |
| baohuoside i /icarisid | PIM2 | SwissTargetPrediction |
| baohuoside i /icarisid | PIM3 | SwissTargetPrediction |
| baohuoside i /icarisid | ABCB1 | SwissTargetPrediction |
| baohuoside i /icarisid | FKBP1A | SwissTargetPrediction |
| baohuoside i /icarisid | FKBP5 | SwissTargetPrediction |
| baohuoside i /icarisid | HDAC1 | SwissTargetPrediction |
| baohuoside i /icarisid | CASP1 | SwissTargetPrediction |
| baohuoside i /icarisid | F11 | SwissTargetPrediction |
| baohuoside i /icarisid | CCND1 CDK4 | SwissTargetPrediction |
| baohuoside i /icarisid | CCNE2 CDK2 CCNE1 | SwissTargetPrediction |
| baohuoside i /icarisid | ACP1 | SwissTargetPrediction |
| baohuoside i /icarisid | CNOT7 | SwissTargetPrediction |
| baohuoside i /icarisid | TDP1 | SwissTargetPrediction |
| baohuoside i /icarisid | EGLN1 | SwissTargetPrediction |
| baohuoside i /icarisid | CXCR2 | SwissTargetPrediction |
| baohuoside i /icarisid | ABL1 | SwissTargetPrediction |
| baohuoside i /icarisid | NOS2 | TCMSP |
| baohuoside i /icarisid | AR | TCMSP |
| baohuoside i /icarisid | PPARG | TCMSP |
| baohuoside i /icarisid | KDR | TCMSP |
| baohuoside i /icarisid | MAPK14 | TCMSP |
| baohuoside i /icarisid | GSK3B | TCMSP |
| baohuoside i /icarisid | PRSS1 | TCMSP |
| baohuoside i /icarisid | CCNA2 | TCMSP |
| baohuoside i /icarisid | NCOA2 | TCMSP |
| wilfortrine | REN | SwissTargetPrediction |
| wilfortrine | PDE10A | SwissTargetPrediction |
| wilfortrine | ADAM17 | SwissTargetPrediction |
| wilfortrine | ADORA1 | SwissTargetPrediction |
| wilfortrine | ADORA2A | SwissTargetPrediction |
| wilfortrine | ADORA2B | SwissTargetPrediction |
| wilfortrine | CTSD | SwissTargetPrediction |
| wilfortrine | PIK3CG | SwissTargetPrediction |
| wilfortrine | TACR1 | SwissTargetPrediction |
| wilfortrine | IGF1R | SwissTargetPrediction |
| wilfortrine | FKBP1A | SwissTargetPrediction |
| wilfortrine | LIPC | SwissTargetPrediction |
| wilfortrine | MTOR | SwissTargetPrediction |
| wilfortrine | CTSS | SwissTargetPrediction |
| wilfortrine | BACE1 | SwissTargetPrediction |
| wilfortrine | LIPG | SwissTargetPrediction |
| wilfortrine | TACR2 | SwissTargetPrediction |
| wilfortrine | MMP13 | SwissTargetPrediction |
| wilfortrine | MMP3 | SwissTargetPrediction |
| wilfortrine | MMP9 | SwissTargetPrediction |
| wilfortrine | MMP1 | SwissTargetPrediction |
| wilfortrine | MMP7 | SwissTargetPrediction |
| wilfortrine | ELANE | SwissTargetPrediction |
| wilfortrine | PIK3CB | SwissTargetPrediction |
| wilfortrine | AVPR2 | SwissTargetPrediction |
| wilfortrine | AVPR1A | SwissTargetPrediction |
| wilfortrine | AVPR1B | SwissTargetPrediction |
| wilfortrine | OXTR | SwissTargetPrediction |
| wilfortrine | EIF4A1 | SwissTargetPrediction |
| wilfortrine | CTSE | SwissTargetPrediction |
| wilfortrine | PGA5 | SwissTargetPrediction |
| wilfortrine | IRAK4 | SwissTargetPrediction |
| wilfortrine | MAPK1 | SwissTargetPrediction |
| wilfortrine | PRKCA | SwissTargetPrediction |
| wilfortrine | CXCR2 | SwissTargetPrediction |
| wilfortrine | CXCR1 | SwissTargetPrediction |
| wilfortrine | CA2 | SwissTargetPrediction |
| wilfortrine | CA7 | SwissTargetPrediction |
| wilfortrine | CA1 | SwissTargetPrediction |
| wilfortrine | NTRK1 | SwissTargetPrediction |
| wilfortrine | CA6 | SwissTargetPrediction |
| wilfortrine | CA12 | SwissTargetPrediction |
| wilfortrine | MMP2 | SwissTargetPrediction |
| wilfortrine | CA14 | SwissTargetPrediction |
| wilfortrine | CA9 | SwissTargetPrediction |
| wilfortrine | CA4 | SwissTargetPrediction |
| wilfortrine | CA13 | SwissTargetPrediction |
| wilfortrine | CA5B | SwissTargetPrediction |
| wilfortrine | MMP8 | SwissTargetPrediction |
| wilfortrine | CA5A | SwissTargetPrediction |
| wilfortrine | IMPDH1 | SwissTargetPrediction |
| wilfortrine | IMPDH2 | SwissTargetPrediction |
| wilfortrine | HDAC1 | SwissTargetPrediction |
| wilfortrine | CTSK | SwissTargetPrediction |
| wilfortrine | AURKB | SwissTargetPrediction |
| wilfortrine | CCNA2 CDK2 | SwissTargetPrediction |
| wilfortrine | HSD11B1 | SwissTargetPrediction |
| wilfortrine | AURKA | SwissTargetPrediction |
| wilfortrine | EDNRA | SwissTargetPrediction |
| wilfortrine | TRAP1 | SwissTargetPrediction |
| wilfortrine | ERBB2 | SwissTargetPrediction |
| wilfortrine | HSP90AA1 | SwissTargetPrediction |
| wilfortrine | HSP90AB1 | SwissTargetPrediction |
| wilfortrine | HRAS | SwissTargetPrediction |
| wilfortrine | BACE2 | SwissTargetPrediction |
| wilfortrine | PRKCD | SwissTargetPrediction |
| wilfortrine | CFD | SwissTargetPrediction |
| wilfortrine | SLC5A2 | SwissTargetPrediction |
| wilfortrine | SIRT2 | SwissTargetPrediction |
| wilfortrine | FLT3 | SwissTargetPrediction |
| wilfortrine | SYK | SwissTargetPrediction |
| wilfortrine | CHEK1 | SwissTargetPrediction |
| wilfortrine | BTK | SwissTargetPrediction |
| wilfortrine | WEE1 | SwissTargetPrediction |
| wilfortrine | TMIGD3 | SwissTargetPrediction |
| wilfortrine | ADAM10 | SwissTargetPrediction |
| wilfortrine | PSMB8 | SwissTargetPrediction |
| wilfortrine | F9 | SwissTargetPrediction |
| wilfortrine | GHRHR | SwissTargetPrediction |
| wilfortrine | EGFR | SwissTargetPrediction |
| wilfortrine | F2 | SwissTargetPrediction |
| wilfortrine | CYP3A4 | SwissTargetPrediction |
| wilfortrine | MCL1 | SwissTargetPrediction |
| wilfortrine | PSMB5 | SwissTargetPrediction |
| wilfortrine | TRPV1 | SwissTargetPrediction |
| wilfortrine | EDNRB | SwissTargetPrediction |
| wilfortrine | F10 | SwissTargetPrediction |
| wilfortrine | CCKBR | SwissTargetPrediction |
| wilfortrine | MET | SwissTargetPrediction |
| wilfortrine | CCKAR | SwissTargetPrediction |
| wilfortrine | PGC | SwissTargetPrediction |
| wilfortrine | TUBB3 | SwissTargetPrediction |
| wilfortrine | AURKC | SwissTargetPrediction |
| wilfortrine | MERTK | SwissTargetPrediction |
| wilfortrine | JAK3 | SwissTargetPrediction |
| wilfortrine | PTAFR | SwissTargetPrediction |
| wilfortrine | CNR2 | SwissTargetPrediction |
| wilfortrine | JAK2 | SwissTargetPrediction |
| wilfortrine | PTPN1 | SwissTargetPrediction |
| wilfortrine | PSMB2 | SwissTargetPrediction |
| peritassine a | MMP1 | SwissTargetPrediction |
| peritassine a | BACE1 | SwissTargetPrediction |
| peritassine a | CCNB3 CDK1 CCNB1 CCNB2 | SwissTargetPrediction |
| peritassine a | CTSD | SwissTargetPrediction |
| peritassine a | CDK1 | SwissTargetPrediction |
| peritassine a | FAP | SwissTargetPrediction |
| peritassine a | BCHE | SwissTargetPrediction |
| peritassine a | PTPN1 | SwissTargetPrediction |
| peritassine a | F9 | SwissTargetPrediction |
| peritassine a | NTRK1 | SwissTargetPrediction |
| peritassine a | FKBP1A | SwissTargetPrediction |
| peritassine a | MAP3K11 | SwissTargetPrediction |
| peritassine a | MAP3K9 | SwissTargetPrediction |
| peritassine a | MAP3K10 | SwissTargetPrediction |
| peritassine a | CETP | SwissTargetPrediction |
| peritassine a | CCNE2 CDK2 CCNE1 | SwissTargetPrediction |
| peritassine a | SRC | SwissTargetPrediction |
| peritassine a | MMP2 | SwissTargetPrediction |
| peritassine a | LTB4R | SwissTargetPrediction |
| peritassine a | CDK2 | SwissTargetPrediction |
| peritassine a | CDK5 | SwissTargetPrediction |
| peritassine a | BDKRB1 | SwissTargetPrediction |
| peritassine a | TYMS | SwissTargetPrediction |
| peritassine a | GBA | SwissTargetPrediction |
| peritassine a | AURKA | SwissTargetPrediction |
| peritassine a | SLC2A1 | SwissTargetPrediction |
| peritassine a | SYK | SwissTargetPrediction |
| peritassine a | CX3CR1 | SwissTargetPrediction |
| peritassine a | FLT3 | SwissTargetPrediction |
| peritassine a | ADORA2B | SwissTargetPrediction |
| peritassine a | PIK3CG | SwissTargetPrediction |
| peritassine a | PTGER3 | SwissTargetPrediction |
| peritassine a | CRHR1 | SwissTargetPrediction |
| peritassine a | RET | SwissTargetPrediction |
| peritassine a | PIK3CD | SwissTargetPrediction |
| peritassine a | PIK3CA | SwissTargetPrediction |
| peritassine a | HSP90AB1 | SwissTargetPrediction |
| peritassine a | ERBB2 | SwissTargetPrediction |
| peritassine a | EGFR | SwissTargetPrediction |
| peritassine a | JAK3 | SwissTargetPrediction |
| peritassine a | CDK7 | SwissTargetPrediction |
| peritassine a | CDK9 | SwissTargetPrediction |
| peritassine a | FBP1 | SwissTargetPrediction |
| peritassine a | HDAC6 | SwissTargetPrediction |
| peritassine a | HDAC1 | SwissTargetPrediction |
| peritassine a | CCKAR | SwissTargetPrediction |
| peritassine a | PSEN1 | SwissTargetPrediction |
| peritassine a | CXCR1 | SwissTargetPrediction |
| peritassine a | IGF1R | SwissTargetPrediction |
| peritassine a | MMP3 | SwissTargetPrediction |
| peritassine a | ABCB1 | SwissTargetPrediction |
| peritassine a | ADAM17 | SwissTargetPrediction |
| peritassine a | CASP3 | SwissTargetPrediction |
| peritassine a | CASP8 | SwissTargetPrediction |
| peritassine a | CASP1 | SwissTargetPrediction |
| peritassine a | PSEN2 PSENEN NCSTN APH1A PSEN1 APH1B | SwissTargetPrediction |
| peritassine a | CDK5R1 CDK5 | SwissTargetPrediction |
| peritassine a | DYRK1A | SwissTargetPrediction |
| peritassine a | MET | SwissTargetPrediction |
| peritassine a | PLAT | SwissTargetPrediction |
| peritassine a | F2 | SwissTargetPrediction |
| peritassine a | PRSS1 | SwissTargetPrediction |
| peritassine a | F10 | SwissTargetPrediction |
| peritassine a | F11 | SwissTargetPrediction |
| peritassine a | PRSS3 | SwissTargetPrediction |
| peritassine a | GCK | SwissTargetPrediction |
| peritassine a | CAPN1 | SwissTargetPrediction |
| peritassine a | PDE5A | SwissTargetPrediction |
| peritassine a | MAPK1 | SwissTargetPrediction |
| peritassine a | CXCR2 | SwissTargetPrediction |
| peritassine a | MAPK14 | SwissTargetPrediction |
| peritassine a | PDE10A | SwissTargetPrediction |
| peritassine a | CA2 | SwissTargetPrediction |
| peritassine a | CA7 | SwissTargetPrediction |
| peritassine a | CA1 | SwissTargetPrediction |
| peritassine a | CA6 | SwissTargetPrediction |
| peritassine a | CA12 | SwissTargetPrediction |
| peritassine a | CA14 | SwissTargetPrediction |
| peritassine a | CA9 | SwissTargetPrediction |
| peritassine a | CA4 | SwissTargetPrediction |
| peritassine a | CA13 | SwissTargetPrediction |
| peritassine a | CA5B | SwissTargetPrediction |
| peritassine a | CA5A | SwissTargetPrediction |
| peritassine a | ESR2 | SwissTargetPrediction |
| peritassine a | PYGL | SwissTargetPrediction |
| peritassine a | MMP9 | SwissTargetPrediction |
| peritassine a | CCNE1 CDK2 | SwissTargetPrediction |
| peritassine a | CDK7 CCNH | SwissTargetPrediction |
| peritassine a | CDK9 CCNT1 | SwissTargetPrediction |
| peritassine a | CTSS | SwissTargetPrediction |
| peritassine a | ADORA1 | SwissTargetPrediction |
| peritassine a | ADAM10 | SwissTargetPrediction |
| peritassine a | LIPC | SwissTargetPrediction |
| peritassine a | LIPG | SwissTargetPrediction |
| peritassine a | ADORA2A | SwissTargetPrediction |
| peritassine a | PAK3 | SwissTargetPrediction |
| peritassine a | PAK2 | SwissTargetPrediction |
| peritassine a | PAK1 | SwissTargetPrediction |
| peritassine a | CDK2 CCNA1 CCNA2 | SwissTargetPrediction |
| peritassine a | CDC7 | SwissTargetPrediction |
| wilfordine | ABCB1 | SwissTargetPrediction |
| wilfordine | PSMB9 | SwissTargetPrediction |
| wilfordine | ADAM17 | SwissTargetPrediction |
| wilfordine | CA2 | SwissTargetPrediction |
| wilfordine | CA7 | SwissTargetPrediction |
| wilfordine | CA1 | SwissTargetPrediction |
| wilfordine | CA6 | SwissTargetPrediction |
| wilfordine | CA12 | SwissTargetPrediction |
| wilfordine | CA14 | SwissTargetPrediction |
| wilfordine | CA9 | SwissTargetPrediction |
| wilfordine | CA4 | SwissTargetPrediction |
| wilfordine | CA13 | SwissTargetPrediction |
| wilfordine | CA5B | SwissTargetPrediction |
| wilfordine | CA5A | SwissTargetPrediction |
| wilfordine | CTSD | SwissTargetPrediction |
| wilfordine | TYMS | SwissTargetPrediction |
| wilfordine | CAPN1 | SwissTargetPrediction |
| wilfordine | PDE5A | SwissTargetPrediction |
| wilfordine | CCNE1 CDK2 | SwissTargetPrediction |
| wilfordine | CDK5R1 CDK5 | SwissTargetPrediction |
| wilfordine | CCNB3 CDK1 CCNB1 CCNB2 | SwissTargetPrediction |
| wilfordine | CDK9 CCNT1 | SwissTargetPrediction |
| wilfordine | MMP13 | SwissTargetPrediction |
| wilfordine | MMP3 | SwissTargetPrediction |
| wilfordine | MMP2 | SwissTargetPrediction |
| wilfordine | MMP14 | SwissTargetPrediction |
| wilfordine | ADAM10 | SwissTargetPrediction |
| wilfordine | CASP3 | SwissTargetPrediction |
| wilfordine | MAPK1 | SwissTargetPrediction |
| wilfordine | DYRK1A | SwissTargetPrediction |
| wilfordine | CDC7 | SwissTargetPrediction |
| wilfordine | CTSS | SwissTargetPrediction |
| wilfordine | PDE10A | SwissTargetPrediction |
| wilfordine | PTPN1 | SwissTargetPrediction |
| wilfordine | PSMB5 | SwissTargetPrediction |
| wilfordine | LIPC | SwissTargetPrediction |
| wilfordine | LIPG | SwissTargetPrediction |
| wilfordine | NTRK1 | SwissTargetPrediction |
| wilfordine | GCK | SwissTargetPrediction |
| wilfordine | PIK3CA | SwissTargetPrediction |
| wilfordine | CFD | SwissTargetPrediction |
| wilfordine | MMP9 | SwissTargetPrediction |
| wilfordine | CASP8 | SwissTargetPrediction |
| wilfordine | MMP8 | SwissTargetPrediction |
| wilfordine | CASP1 | SwissTargetPrediction |
| wilfordine | PRKCA | SwissTargetPrediction |
| wilfordine | FBP1 | SwissTargetPrediction |
| wilfordine | HSPA5 | SwissTargetPrediction |
| wilfordine | SYK | SwissTargetPrediction |
| wilfordine | CDK1 | SwissTargetPrediction |
| wilfordine | BACE1 | SwissTargetPrediction |
| wilfordine | HRH1 | SwissTargetPrediction |
| wilfordine | MTOR | SwissTargetPrediction |
| wilfordine | CCR3 | SwissTargetPrediction |
| wilfordine | IRAK4 | SwissTargetPrediction |
| wilfordine | CCKBR | SwissTargetPrediction |
| wilfordine | ADCY1 | SwissTargetPrediction |
| wilfordine | STAT3 | SwissTargetPrediction |
| wilfordine | SLC2A1 | SwissTargetPrediction |
| wilfordine | P2RX3 | SwissTargetPrediction |
| wilfordine | AVPR2 | SwissTargetPrediction |
| wilfordine | AVPR1B | SwissTargetPrediction |
| wilfordine | OXTR | SwissTargetPrediction |
| wilfordine | RASGRP3 | SwissTargetPrediction |
| wilfordine | BDKRB1 | SwissTargetPrediction |
| wilfordine | MCL1 | SwissTargetPrediction |
| wilfordine | AURKB | SwissTargetPrediction |
| wilfordine | TACR2 | SwissTargetPrediction |
| wilfordine | TACR1 | SwissTargetPrediction |
| wilfordine | PYGL | SwissTargetPrediction |
| wilfordine | CASP6 | SwissTargetPrediction |
| wilfordine | CASP7 | SwissTargetPrediction |
| wilfordine | AURKA | SwissTargetPrediction |
| wilfordine | CDK7 CCNH | SwissTargetPrediction |
| wilfordine | CXCR2 | SwissTargetPrediction |
| wilfordine | CXCR1 | SwissTargetPrediction |
| wilfordine | EDNRB | SwissTargetPrediction |
| wilfordine | PIK3CA PIK3R1 | SwissTargetPrediction |
| wilfordine | EDNRA | SwissTargetPrediction |
| wilfordine | SRC | SwissTargetPrediction |
| wilfordine | IDO1 | SwissTargetPrediction |
| wilfordine | ESR2 | SwissTargetPrediction |
| wilfordine | PRKCD | SwissTargetPrediction |
| wilfordine | MET | SwissTargetPrediction |
| wilfordine | PDPK1 | SwissTargetPrediction |
| wilfordine | PAK3 | SwissTargetPrediction |
| wilfordine | PAK2 | SwissTargetPrediction |
| wilfordine | PAK1 | SwissTargetPrediction |
| wilfordine | F9 | SwissTargetPrediction |
| wilfordine | TNNC1 TNNT2 TNNI3 | SwissTargetPrediction |
| wilfordine | MAPK14 | SwissTargetPrediction |
| wilfordine | HK2 | SwissTargetPrediction |
| wilfordine | HK1 | SwissTargetPrediction |
| wilfordine | PIK3CG | SwissTargetPrediction |
| wilfordine | NOS2 | SwissTargetPrediction |
| wilfordine | BTK | SwissTargetPrediction |
| wilfordine | ERBB2 | SwissTargetPrediction |
| wilfordine | AR | SwissTargetPrediction |
| wilfordine | EGFR | SwissTargetPrediction |
| wilfordine | MMP1 | SwissTargetPrediction |
| wilfornine d | ABCB1 | SwissTargetPrediction |
| wilfornine d | ADAM17 | SwissTargetPrediction |
| wilfornine d | PTPN1 | SwissTargetPrediction |
| wilfornine d | CES2 | SwissTargetPrediction |
| wilfornine d | PDE5A | SwissTargetPrediction |
| wilfornine d | AURKB | SwissTargetPrediction |
| wilfornine d | AURKA | SwissTargetPrediction |
| wilfornine d | CAPN1 | SwissTargetPrediction |
| wilfornine d | CTSS | SwissTargetPrediction |
| wilfornine d | CDK5R1 CDK5 | SwissTargetPrediction |
| wilfornine d | DYRK1A | SwissTargetPrediction |
| wilfornine d | CDC7 | SwissTargetPrediction |
| wilfornine d | PRKCA | SwissTargetPrediction |
| wilfornine d | GCK | SwissTargetPrediction |
| wilfornine d | REN | SwissTargetPrediction |
| wilfornine d | ADAM10 | SwissTargetPrediction |
| wilfornine d | ADORA1 | SwissTargetPrediction |
| wilfornine d | CASP3 | SwissTargetPrediction |
| wilfornine d | ADORA2A | SwissTargetPrediction |
| wilfornine d | ADORA3 | SwissTargetPrediction |
| wilfornine d | CASP8 | SwissTargetPrediction |
| wilfornine d | CASP1 | SwissTargetPrediction |
| wilfornine d | CA2 | SwissTargetPrediction |
| wilfornine d | CA7 | SwissTargetPrediction |
| wilfornine d | CA1 | SwissTargetPrediction |
| wilfornine d | CA6 | SwissTargetPrediction |
| wilfornine d | CA12 | SwissTargetPrediction |
| wilfornine d | CA14 | SwissTargetPrediction |
| wilfornine d | CA9 | SwissTargetPrediction |
| wilfornine d | CA4 | SwissTargetPrediction |
| wilfornine d | CA13 | SwissTargetPrediction |
| wilfornine d | CA5B | SwissTargetPrediction |
| wilfornine d | PDE10A | SwissTargetPrediction |
| wilfornine d | CA5A | SwissTargetPrediction |
| wilfornine d | KDM1A | SwissTargetPrediction |
| wilfornine d | PYGL | SwissTargetPrediction |
| wilfornine d | CTSD | SwissTargetPrediction |
| wilfornine d | NTRK1 | SwissTargetPrediction |
| wilfornine d | HSP90AB1 | SwissTargetPrediction |
| wilfornine d | MAPK14 | SwissTargetPrediction |
| wilfornine d | CCNE1 CDK2 | SwissTargetPrediction |
| wilfornine d | ESR2 | SwissTargetPrediction |
| wilfornine d | RASGRP3 | SwissTargetPrediction |
| wilfornine d | MMP13 | SwissTargetPrediction |
| wilfornine d | MMP12 | SwissTargetPrediction |
| wilfornine d | CXCR2 | SwissTargetPrediction |
| wilfornine d | KCNA3 | SwissTargetPrediction |
| wilfornine d | CDK7 CCNH | SwissTargetPrediction |
| wilfornine d | CDK9 CCNT1 | SwissTargetPrediction |
| wilfornine d | CFD | SwissTargetPrediction |
| wilfornine d | MAPK1 | SwissTargetPrediction |
| wilfornine d | SYK | SwissTargetPrediction |
| wilfornine d | PAK3 | SwissTargetPrediction |
| wilfornine d | PAK2 | SwissTargetPrediction |
| wilfornine d | PAK1 | SwissTargetPrediction |
| wilfornine d | CXCR1 | SwissTargetPrediction |
| wilfornine d | MMP8 | SwissTargetPrediction |
| wilfornine d | PLAT | SwissTargetPrediction |
| wilfornine d | TYMS | SwissTargetPrediction |
| wilfornine d | F2 | SwissTargetPrediction |
| wilfornine d | CDK2 CCNA1 CCNA2 | SwissTargetPrediction |
| wilfornine d | PRSS1 | SwissTargetPrediction |
| wilfornine d | F10 | SwissTargetPrediction |
| wilfornine d | F11 | SwissTargetPrediction |
| wilfornine d | PRSS3 | SwissTargetPrediction |
| wilfornine d | BACE1 | SwissTargetPrediction |
| wilfornine d | FBP1 | SwissTargetPrediction |
| wilfornine d | EDNRB | SwissTargetPrediction |
| wilfornine d | TACR2 | SwissTargetPrediction |
| wilfornine d | EDNRA | SwissTargetPrediction |
| wilfornine d | MAP3K11 | SwissTargetPrediction |
| wilfornine d | MAP3K9 | SwissTargetPrediction |
| wilfornine d | MAP3K10 | SwissTargetPrediction |
| wilfornine d | MMP9 | SwissTargetPrediction |
| wilfornine d | IGF1R | SwissTargetPrediction |
| wilfornine d | LIPC | SwissTargetPrediction |
| wilfornine d | CCR6 | SwissTargetPrediction |
| wilfornine d | CXCR3 | SwissTargetPrediction |
| wilfornine d | SLC5A1 | SwissTargetPrediction |
| wilfornine d | LIPG | SwissTargetPrediction |
| wilfornine d | CHRM4 | SwissTargetPrediction |
| wilfornine d | CHRM5 | SwissTargetPrediction |
| wilfornine d | CCNB3 CDK1 CCNB1 CCNB2 | SwissTargetPrediction |
| wilfornine d | CHRM2 | SwissTargetPrediction |
| wilfornine d | CHRM1 | SwissTargetPrediction |
| wilfornine d | CHRM3 | SwissTargetPrediction |
| wilfornine d | CDK1 | SwissTargetPrediction |
| wilfornine d | MET | SwissTargetPrediction |
| wilfornine d | MMP3 | SwissTargetPrediction |
| wilfornine d | MMP2 | SwissTargetPrediction |
| wilfornine d | MMP14 | SwissTargetPrediction |
| wilfornine d | PIK3CG | SwissTargetPrediction |
| wilfornine d | PTGER3 | SwissTargetPrediction |
| wilfornine d | HSPA5 | SwissTargetPrediction |
| wilfornine d | AVPR2 | SwissTargetPrediction |
| wilfornine d | AVPR1A | SwissTargetPrediction |
| wilfornine d | AVPR1B | SwissTargetPrediction |
| wilfornine d | OXTR | SwissTargetPrediction |
| wilfornine d | FAAH | SwissTargetPrediction |
| wilfornine d | STAT3 | SwissTargetPrediction |
| wilforgine | REN | SwissTargetPrediction |
| wilforgine | PDE10A | SwissTargetPrediction |
| wilforgine | TACR1 | SwissTargetPrediction |
| wilforgine | CTSK | SwissTargetPrediction |
| wilforgine | PIK3CG | SwissTargetPrediction |
| wilforgine | ADAM17 | SwissTargetPrediction |
| wilforgine | CTSD | SwissTargetPrediction |
| wilforgine | CTSE | SwissTargetPrediction |
| wilforgine | BACE1 | SwissTargetPrediction |
| wilforgine | IGF1R | SwissTargetPrediction |
| wilforgine | CA2 | SwissTargetPrediction |
| wilforgine | CA7 | SwissTargetPrediction |
| wilforgine | CA1 | SwissTargetPrediction |
| wilforgine | CA6 | SwissTargetPrediction |
| wilforgine | MMP9 | SwissTargetPrediction |
| wilforgine | CA12 | SwissTargetPrediction |
| wilforgine | MMP2 | SwissTargetPrediction |
| wilforgine | CA14 | SwissTargetPrediction |
| wilforgine | CA9 | SwissTargetPrediction |
| wilforgine | CA4 | SwissTargetPrediction |
| wilforgine | CA13 | SwissTargetPrediction |
| wilforgine | CA5B | SwissTargetPrediction |
| wilforgine | F2R | SwissTargetPrediction |
| wilforgine | CA5A | SwissTargetPrediction |
| wilforgine | PRKCD | SwissTargetPrediction |
| wilforgine | PRKCA | SwissTargetPrediction |
| wilforgine | HDAC1 | SwissTargetPrediction |
| wilforgine | NTRK1 | SwissTargetPrediction |
| wilforgine | CXCR2 | SwissTargetPrediction |
| wilforgine | CXCR1 | SwissTargetPrediction |
| wilforgine | LIPC | SwissTargetPrediction |
| wilforgine | PGA5 | SwissTargetPrediction |
| wilforgine | LIPG | SwissTargetPrediction |
| wilforgine | F2 | SwissTargetPrediction |
| wilforgine | GBA | SwissTargetPrediction |
| wilforgine | MMP13 | SwissTargetPrediction |
| wilforgine | MMP3 | SwissTargetPrediction |
| wilforgine | MMP1 | SwissTargetPrediction |
| wilforgine | MMP7 | SwissTargetPrediction |
| wilforgine | BCL2 | SwissTargetPrediction |
| wilforgine | PGC | SwissTargetPrediction |
| wilforgine | ELANE | SwissTargetPrediction |
| wilforgine | MTOR | SwissTargetPrediction |
| wilforgine | PTAFR | SwissTargetPrediction |
| wilforgine | CCNA2 CDK2 | SwissTargetPrediction |
| wilforgine | MAPK1 | SwissTargetPrediction |
| wilforgine | FGFR2 | SwissTargetPrediction |
| wilforgine | INSR | SwissTargetPrediction |
| wilforgine | EGFR | SwissTargetPrediction |
| wilforgine | ADORA1 | SwissTargetPrediction |
| wilforgine | ADORA2A | SwissTargetPrediction |
| wilforgine | ADORA2B | SwissTargetPrediction |
| wilforgine | LCK | SwissTargetPrediction |
| wilforgine | PTK2 | SwissTargetPrediction |
| wilforgine | KDR | SwissTargetPrediction |
| wilforgine | CTSS | SwissTargetPrediction |
| wilforgine | MAP2K1 | SwissTargetPrediction |
| wilforgine | MET | SwissTargetPrediction |
| wilforgine | ESR1 | SwissTargetPrediction |
| wilforgine | SAE1 UBA2 | SwissTargetPrediction |
| wilforgine | CFD | SwissTargetPrediction |
| wilforgine | EDNRA | SwissTargetPrediction |
| wilforgine | EPHB4 | SwissTargetPrediction |
| wilforgine | CCKAR | SwissTargetPrediction |
| wilforgine | JAK3 | SwissTargetPrediction |
| wilforgine | F10 | SwissTargetPrediction |
| wilforgine | SRC | SwissTargetPrediction |
| wilforgine | JAK2 | SwissTargetPrediction |
| wilforgine | CCKBR | SwissTargetPrediction |
| wilforgine | IMPDH1 | SwissTargetPrediction |
| wilforgine | IMPDH2 | SwissTargetPrediction |
| wilforgine | IRAK4 | SwissTargetPrediction |
| wilforgine | CCR6 | SwissTargetPrediction |
| wilforgine | CXCR3 | SwissTargetPrediction |
| wilforgine | ERBB2 | SwissTargetPrediction |
| wilforgine | P2RX3 | SwissTargetPrediction |
| wilforgine | HSP90AB1 | SwissTargetPrediction |
| wilforgine | PSMB5 | SwissTargetPrediction |
| wilforgine | ADAM10 | SwissTargetPrediction |
| wilforgine | AURKB | SwissTargetPrediction |
| wilforgine | CNR2 | SwissTargetPrediction |
| wilforgine | AURKC | SwissTargetPrediction |
| wilforgine | AURKA | SwissTargetPrediction |
| wilforgine | KLK3 | SwissTargetPrediction |
| wilforgine | HSP90AA1 | SwissTargetPrediction |
| wilforgine | HSD11B1 | SwissTargetPrediction |
| wilforgine | PDE5A | SwissTargetPrediction |
| wilforgine | PDE4B | SwissTargetPrediction |
| wilforgine | EDNRB | SwissTargetPrediction |
| wilforgine | SCARB1 | SwissTargetPrediction |
| wilforgine | MMP14 | SwissTargetPrediction |
| wilforgine | MMP12 | SwissTargetPrediction |
| wilforgine | SIRT2 | SwissTargetPrediction |
| wilforgine | MMP8 | SwissTargetPrediction |
| wilforgine | CDK5R1 CDK5 | SwissTargetPrediction |
| wilforgine | GHRHR | SwissTargetPrediction |
| wilforgine | DYRK1A | SwissTargetPrediction |
| wilforgine | TACR2 | SwissTargetPrediction |
| wilforgine | KDM5A | SwissTargetPrediction |
| wilforgine | PIK3CB | SwissTargetPrediction |
| triptophenolide | NEK1 | SwissTargetPrediction |
| triptophenolide | PARP1 | SwissTargetPrediction |
| triptophenolide | PRKCZ | SwissTargetPrediction |
| triptophenolide | DRD2 | SwissTargetPrediction |
| triptophenolide | PLA2G7 | SwissTargetPrediction |
| triptophenolide | DRD4 | SwissTargetPrediction |
| triptophenolide | HTR2A | SwissTargetPrediction |
| triptophenolide | HTR1B | SwissTargetPrediction |
| triptophenolide | KIF11 | SwissTargetPrediction |
| triptophenolide | NISCH | SwissTargetPrediction |
| triptophenolide | ERBB2 | SwissTargetPrediction |
| triptophenolide | EGFR | SwissTargetPrediction |
| triptophenolide | SRD5A2 | SwissTargetPrediction |
| triptophenolide | DAPK3 | SwissTargetPrediction |
| triptophenolide | DAPK1 | SwissTargetPrediction |
| triptophenolide | DAPK2 | SwissTargetPrediction |
| triptophenolide | STK17B | SwissTargetPrediction |
| triptophenolide | STK17A | SwissTargetPrediction |
| triptophenolide | PABPC1 | SwissTargetPrediction |
| triptophenolide | FLT1 | SwissTargetPrediction |
| triptophenolide | HTR1D | SwissTargetPrediction |
| triptophenolide | ADRA1D | TCMSP |
| triptophenolide | ADRA1A | TCMSP |
| triptophenolide | ADRA1B | TCMSP |
| triptophenolide | BRAF | SwissTargetPrediction |
| triptophenolide | BACE2 | SwissTargetPrediction |
| triptophenolide | HSD17B1 | SwissTargetPrediction |
| triptophenolide | ADORA2A | SwissTargetPrediction |
| triptophenolide | EEF2K | SwissTargetPrediction |
| triptophenolide | SRD5A1 | SwissTargetPrediction |
| triptophenolide | DRD1 | SwissTargetPrediction |
| triptophenolide | TSPO | SwissTargetPrediction |
| triptophenolide | CDK2 | SwissTargetPrediction |
| triptophenolide | CDK4 | SwissTargetPrediction |
| triptophenolide | HDAC6 | SwissTargetPrediction |
| triptophenolide | MIF | SwissTargetPrediction |
| triptophenolide | HDAC1 | SwissTargetPrediction |
| triptophenolide | CDK5R1 CDK5 | SwissTargetPrediction |
| triptophenolide | PSEN2 PSENEN NCSTN APH1A PSEN1 APH1B | SwissTargetPrediction |
| triptophenolide | MAPKAPK2 | SwissTargetPrediction |
| triptophenolide | DYRK1A | SwissTargetPrediction |
| triptophenolide | EPHX2 | SwissTargetPrediction |
| triptophenolide | S1PR3 | SwissTargetPrediction |
| triptophenolide | GRM5 | SwissTargetPrediction |
| triptophenolide | AURKC | SwissTargetPrediction |
| triptophenolide | PTK6 | SwissTargetPrediction |
| triptophenolide | AURKA | SwissTargetPrediction |
| triptophenolide | ACVR1 | SwissTargetPrediction |
| triptophenolide | ADRA2A | SwissTargetPrediction |
| triptophenolide | ADRA2C | SwissTargetPrediction |
| triptophenolide | ADRA2B | SwissTargetPrediction |
| triptophenolide | JAK3 | SwissTargetPrediction |
| triptophenolide | JAK2 | SwissTargetPrediction |
| triptophenolide | MAP4K5 | SwissTargetPrediction |
| triptophenolide | MAP4K3 | SwissTargetPrediction |
| triptophenolide | COMT | SwissTargetPrediction |
| triptophenolide | CASR | SwissTargetPrediction |
| triptophenolide | TYMS | SwissTargetPrediction |
| triptophenolide | VDR | SwissTargetPrediction |
| triptophenolide | MGLL | SwissTargetPrediction |
| triptophenolide | LTA4H | SwissTargetPrediction |
| triptophenolide | F10 | SwissTargetPrediction |
| triptophenolide | HCRTR2 | SwissTargetPrediction |
| triptophenolide | F2 | SwissTargetPrediction |
| triptophenolide | PRSS1 | SwissTargetPrediction |
| triptophenolide | CTRC | SwissTargetPrediction |
| triptophenolide | MAPK14 | SwissTargetPrediction |
| triptophenolide | MMP13 | SwissTargetPrediction |
| triptophenolide | MMP1 | SwissTargetPrediction |
| triptophenolide | ADAM17 | SwissTargetPrediction |
| triptophenolide | JAK1 | SwissTargetPrediction |
| triptophenolide | HDAC8 | SwissTargetPrediction |
| triptophenolide | DCTPP1 | SwissTargetPrediction |
| triptophenolide | CYP24A1 | SwissTargetPrediction |
| triptophenolide | CNR1 | SwissTargetPrediction |
| triptophenolide | FAAH | SwissTargetPrediction |
| triptophenolide | RPS6KA3 | SwissTargetPrediction |
| triptophenolide | P2RX7 | SwissTargetPrediction |
| triptophenolide | HDAC3 | SwissTargetPrediction |
| triptophenolide | NR1H4 | SwissTargetPrediction |
| triptophenolide | GCGR | SwissTargetPrediction |
| triptophenolide | ROCK2 | SwissTargetPrediction |
| triptophenolide | PLA2G2A | SwissTargetPrediction |
| triptophenolide | LIMK1 | SwissTargetPrediction |
| triptophenolide | TAAR1 | SwissTargetPrediction |
| triptophenolide | LIMK2 | SwissTargetPrediction |
| triptophenolide | CSF1R | SwissTargetPrediction |
| triptophenolide | MTNR1A | SwissTargetPrediction |
| triptophenolide | MTNR1B | SwissTargetPrediction |
| triptophenolide | ELANE | SwissTargetPrediction |
| triptophenolide | CTSD | SwissTargetPrediction |
| triptophenolide | CALCRL | SwissTargetPrediction |
| triptophenolide | CXCR3 | SwissTargetPrediction |
| triptophenolide | RAF1 | SwissTargetPrediction |
| triptophenolide | PYGL | SwissTargetPrediction |
| triptophenolide | CCKBR | SwissTargetPrediction |
| triptophenolide | CDK9 | SwissTargetPrediction |
| triptophenolide | LDLR | SwissTargetPrediction |
| triptophenolide | PIK3CA PIK3R1 | SwissTargetPrediction |
| triptophenolide | HDAC7 | SwissTargetPrediction |
| triptophenolide | CHRM3 | TCMSP |
| triptophenolide | KCNH2 | TCMSP |
| triptophenolide | CHRM1 | TCMSP |
| triptophenolide | SCN5A | TCMSP |
| triptophenolide | CHRM5 | TCMSP |
| triptophenolide | PTGS2 | TCMSP |
| triptophenolide | RXRA | TCMSP |
| triptophenolide | OPRD1 | TCMSP |
| triptophenolide | PGR | TCMSP |
| triptophenolide | CHRM2 | TCMSP |
| triptophenolide | ADRB2 | TCMSP |
| triptophenolide | OPRM1 | TCMSP |
| triptophenolide | NCOA2 | TCMSP |
| triptophenolide | NCOA1 | TCMSP |
| euonine | CXCR2 | SwissTargetPrediction |
| euonine | FKBP1A | SwissTargetPrediction |
| euonine | CFD | SwissTargetPrediction |
| euonine | TACR2 | SwissTargetPrediction |
| euonine | TACR1 | SwissTargetPrediction |
| euonine | ELANE | SwissTargetPrediction |
| euonine | CTSG | SwissTargetPrediction |
| euonine | CCNB3 CDK1 CCNB1 CCNB2 | SwissTargetPrediction |
| euonine | PRKCA | SwissTargetPrediction |
| euonine | CDK2 | SwissTargetPrediction |
| euonine | CDK1 | SwissTargetPrediction |
| euonine | PRKCE | SwissTargetPrediction |
| euonine | RASGRP3 | SwissTargetPrediction |
| euonine | RASGRP1 | SwissTargetPrediction |
| euonine | CASP8 | SwissTargetPrediction |
| euonine | PAK4 | SwissTargetPrediction |
| euonine | PAK1 | SwissTargetPrediction |
| euonine | P2RX3 | SwissTargetPrediction |
| euonine | GCK | SwissTargetPrediction |
| euonine | MMP7 | SwissTargetPrediction |
| euonine | BACE1 | SwissTargetPrediction |
| euonine | CDK5R1 CDK5 | SwissTargetPrediction |
| euonine | CDK2 CCNA1 CCNA2 | SwissTargetPrediction |
| euonine | ADORA1 | SwissTargetPrediction |
| euonine | DYRK1A | SwissTargetPrediction |
| euonine | CASP3 | SwissTargetPrediction |
| euonine | ADORA2B | SwissTargetPrediction |
| euonine | NOS2 | SwissTargetPrediction |
| euonine | TRPA1 | SwissTargetPrediction |
| euonine | PIK3CG | SwissTargetPrediction |
| euonine | CHRM4 | SwissTargetPrediction |
| euonine | CHRM5 | SwissTargetPrediction |
| euonine | CHRM2 | SwissTargetPrediction |
| euonine | CHRM1 | SwissTargetPrediction |
| euonine | CHRM3 | SwissTargetPrediction |
| euonine | CTSS | SwissTargetPrediction |
| euonine | AGTR1 | SwissTargetPrediction |
| euonine | MMP14 | SwissTargetPrediction |
| euonine | KLK7 | SwissTargetPrediction |
| euonine | SYK | SwissTargetPrediction |
| euonine | EDNRB | SwissTargetPrediction |
| euonine | EDNRA | SwissTargetPrediction |
| euonine | MTOR | SwissTargetPrediction |
| euonine | PIK3CA | SwissTargetPrediction |
| euonine | BDKRB1 | SwissTargetPrediction |
| euonine | ADCY1 | SwissTargetPrediction |
| euonine | CTSK | SwissTargetPrediction |
| euonine | MMP3 | SwissTargetPrediction |
| euonine | MMP9 | SwissTargetPrediction |
| euonine | CASP6 | SwissTargetPrediction |
| euonine | CASP7 | SwissTargetPrediction |
| euonine | HDAC6 | SwissTargetPrediction |
| euonine | NCOR2 HDAC3 | SwissTargetPrediction |
| euonine | CAPN2 | SwissTargetPrediction |
| euonine | HDAC8 | SwissTargetPrediction |
| euonine | IRAK4 | SwissTargetPrediction |
| euonine | SRC | SwissTargetPrediction |
| euonine | CPT1A | SwissTargetPrediction |
| euonine | SEM1 PSMD8 PSMD4 PSMD13 PSMD7 PSMD6 PSMD11 PSMD12 PSMD3 PSMD2 PSMC5 PSMC3 PSMC6 PSMC4 PSMC1 PSMC2 ADRM1 PSMD14 PSMD1 PSMB7 PSMB4 PSMB3 PSMB11 PSMB10 PSMA8 PSMA7 PSMA6 PSMA5 PSMA4 PSMA3 PSMA2 PSMA1 PSMB6 PSMB9 PSMB8 PSMB5 PSMB1 PSMB2 | SwissTargetPrediction |
| euonine | PIK3CB | SwissTargetPrediction |
| euonine | TNF | SwissTargetPrediction |
| euonine | PSMB9 | SwissTargetPrediction |
| euonine | IMPDH2 | SwissTargetPrediction |
| euonine | LIPC | SwissTargetPrediction |
| euonine | PTAFR | SwissTargetPrediction |
| euonine | SLC2A1 | SwissTargetPrediction |
| euonine | STAT3 | SwissTargetPrediction |
| euonine | HSP90AB1 | SwissTargetPrediction |
| euonine | KLK5 | SwissTargetPrediction |
| euonine | LIPG | SwissTargetPrediction |
| euonine | LIMK2 | SwissTargetPrediction |
| euonine | TYMS | SwissTargetPrediction |
| euonine | EGFR | SwissTargetPrediction |
| euonine | PYGL | SwissTargetPrediction |
| euonine | FCER2 | SwissTargetPrediction |
| euonine | MET | SwissTargetPrediction |
| euonine | CX3CR1 | SwissTargetPrediction |
| euonine | ABCB1 | SwissTargetPrediction |
| euonine | PDE10A | SwissTargetPrediction |
| euonine | PTPN1 | SwissTargetPrediction |
| euonine | IGF1R | SwissTargetPrediction |
| euonine | ADAM17 | SwissTargetPrediction |
| euonine | PSMB5 | SwissTargetPrediction |
| euonine | MMP1 | SwissTargetPrediction |
| euonine | AVPR2 | SwissTargetPrediction |
| euonine | AVPR1B | SwissTargetPrediction |
| euonine | OXTR | SwissTargetPrediction |
| euonine | MMP13 | SwissTargetPrediction |
| euonine | MMP12 | SwissTargetPrediction |
| euonine | ADAM10 | SwissTargetPrediction |
| euonine | CTSD | SwissTargetPrediction |
| euonine | PDE5A | SwissTargetPrediction |
| euonine | AR | SwissTargetPrediction |
| euonine | CDC7 | SwissTargetPrediction |
| euonine | AURKB | SwissTargetPrediction |
| euonine | CAPN1 | SwissTargetPrediction |
| euonine | AURKA | SwissTargetPrediction |
| euonine | F2 | SwissTargetPrediction |
| euonine | F10 | SwissTargetPrediction |
| euonine | CASP1 | SwissTargetPrediction |
| wilfornine a | ADAM17 | SwissTargetPrediction |
| wilfornine a | PDE10A | SwissTargetPrediction |
| wilfornine a | ABCB1 | SwissTargetPrediction |
| wilfornine a | CASP3 | SwissTargetPrediction |
| wilfornine a | CASP8 | SwissTargetPrediction |
| wilfornine a | CAPN1 | SwissTargetPrediction |
| wilfornine a | CDK5R1 CDK5 | SwissTargetPrediction |
| wilfornine a | DYRK1A | SwissTargetPrediction |
| wilfornine a | CTSD | SwissTargetPrediction |
| wilfornine a | TACR2 | SwissTargetPrediction |
| wilfornine a | ADAM10 | SwissTargetPrediction |
| wilfornine a | AURKB | SwissTargetPrediction |
| wilfornine a | CTSS | SwissTargetPrediction |
| wilfornine a | AURKA | SwissTargetPrediction |
| wilfornine a | AVPR2 | SwissTargetPrediction |
| wilfornine a | AVPR1B | SwissTargetPrediction |
| wilfornine a | OXTR | SwissTargetPrediction |
| wilfornine a | ESR2 | SwissTargetPrediction |
| wilfornine a | KCNA3 | SwissTargetPrediction |
| wilfornine a | CCNE1 CDK2 | SwissTargetPrediction |
| wilfornine a | CA2 | SwissTargetPrediction |
| wilfornine a | CDK7 CCNH | SwissTargetPrediction |
| wilfornine a | CDK9 CCNT1 | SwissTargetPrediction |
| wilfornine a | CA7 | SwissTargetPrediction |
| wilfornine a | CA1 | SwissTargetPrediction |
| wilfornine a | CA6 | SwissTargetPrediction |
| wilfornine a | CA12 | SwissTargetPrediction |
| wilfornine a | CA14 | SwissTargetPrediction |
| wilfornine a | CA9 | SwissTargetPrediction |
| wilfornine a | CA4 | SwissTargetPrediction |
| wilfornine a | CA13 | SwissTargetPrediction |
| wilfornine a | CA5B | SwissTargetPrediction |
| wilfornine a | CA5A | SwissTargetPrediction |
| wilfornine a | CES2 | SwissTargetPrediction |
| wilfornine a | MAPK1 | SwissTargetPrediction |
| wilfornine a | KDM1A | SwissTargetPrediction |
| wilfornine a | NTRK1 | SwissTargetPrediction |
| wilfornine a | CCNB3 CDK1 CCNB1 CCNB2 | SwissTargetPrediction |
| wilfornine a | SYK | SwissTargetPrediction |
| wilfornine a | CDK1 | SwissTargetPrediction |
| wilfornine a | CXCR2 | SwissTargetPrediction |
| wilfornine a | CXCR1 | SwissTargetPrediction |
| wilfornine a | CX3CR1 | SwissTargetPrediction |
| wilfornine a | BACE1 | SwissTargetPrediction |
| wilfornine a | PDE5A | SwissTargetPrediction |
| wilfornine a | CFD | SwissTargetPrediction |
| wilfornine a | PYGL | SwissTargetPrediction |
| wilfornine a | PRKCA | SwissTargetPrediction |
| wilfornine a | MAPK14 | SwissTargetPrediction |
| wilfornine a | FAP | SwissTargetPrediction |
| wilfornine a | IGF1R | SwissTargetPrediction |
| wilfornine a | RASGRP3 | SwissTargetPrediction |
| wilfornine a | CDK2 CCNA1 CCNA2 | SwissTargetPrediction |
| wilfornine a | MAP3K11 | SwissTargetPrediction |
| wilfornine a | MAP3K9 | SwissTargetPrediction |
| wilfornine a | MAP3K10 | SwissTargetPrediction |
| wilfornine a | PAK3 | SwissTargetPrediction |
| wilfornine a | PARP1 | SwissTargetPrediction |
| wilfornine a | GCK | SwissTargetPrediction |
| wilfornine a | PAK2 | SwissTargetPrediction |
| wilfornine a | PAK1 | SwissTargetPrediction |
| wilfornine a | FAAH | SwissTargetPrediction |
| wilfornine a | JAK3 | SwissTargetPrediction |
| wilfornine a | MMP3 | SwissTargetPrediction |
| wilfornine a | JAK2 | SwissTargetPrediction |
| wilfornine a | CTSE | SwissTargetPrediction |
| wilfornine a | MMP2 | SwissTargetPrediction |
| wilfornine a | EGFR | SwissTargetPrediction |
| wilfornine a | PLAT | SwissTargetPrediction |
| wilfornine a | F2 | SwissTargetPrediction |
| wilfornine a | PRSS1 | SwissTargetPrediction |
| wilfornine a | LIPC | SwissTargetPrediction |
| wilfornine a | F10 | SwissTargetPrediction |
| wilfornine a | SRC | SwissTargetPrediction |
| wilfornine a | F11 | SwissTargetPrediction |
| wilfornine a | PRSS3 | SwissTargetPrediction |
| wilfornine a | LIPG | SwissTargetPrediction |
| wilfornine a | FKBP1A | SwissTargetPrediction |
| wilfornine a | GBA | SwissTargetPrediction |
| wilfornine a | CDK2 | SwissTargetPrediction |
| wilfornine a | CDK7 | SwissTargetPrediction |
| wilfornine a | CDK9 | SwissTargetPrediction |
| wilfornine a | CDK5 | SwissTargetPrediction |
| wilfornine a | PRKCD | SwissTargetPrediction |
| wilfornine a | KLK5 | SwissTargetPrediction |
| wilfornine a | BDKRB1 | SwissTargetPrediction |
| wilfornine a | EDNRB | SwissTargetPrediction |
| wilfornine a | EDNRA | SwissTargetPrediction |
| wilfornine a | CTSK | SwissTargetPrediction |
| wilfornine a | KDR | SwissTargetPrediction |
| wilfornine a | MMP9 | SwissTargetPrediction |
| wilfornine a | KLK7 | SwissTargetPrediction |
| wilfornine a | ADORA2B | SwissTargetPrediction |
| wilfornine a | PSMB5 | SwissTargetPrediction |
| wilfornine a | ZAP70 | SwissTargetPrediction |
| wilfornine a | MET | SwissTargetPrediction |
| wilfornine a | BTK | SwissTargetPrediction |
| wilfornine a | CRHR1 | SwissTargetPrediction |
| wilfornine a | CDK1 CCNB1 | SwissTargetPrediction |
| wilfornine a | BACE2 | SwissTargetPrediction |
| wilforine | ABCB1 | SwissTargetPrediction |
| wilforine | IGF1R | SwissTargetPrediction |
| wilforine | PDE10A | SwissTargetPrediction |
| wilforine | PTPN1 | SwissTargetPrediction |
| wilforine | ELANE | SwissTargetPrediction |
| wilforine | CTSD | SwissTargetPrediction |
| wilforine | AVPR2 | SwissTargetPrediction |
| wilforine | AVPR1B | SwissTargetPrediction |
| wilforine | OXTR | SwissTargetPrediction |
| wilforine | F2 | SwissTargetPrediction |
| wilforine | ADAM17 | SwissTargetPrediction |
| wilforine | CCNB3 CDK1 CCNB1 CCNB2 | SwissTargetPrediction |
| wilforine | CTSS | SwissTargetPrediction |
| wilforine | P2RX3 | SwissTargetPrediction |
| wilforine | CDK2 | SwissTargetPrediction |
| wilforine | CDK1 | SwissTargetPrediction |
| wilforine | MMP1 | SwissTargetPrediction |
| wilforine | MMP13 | SwissTargetPrediction |
| wilforine | MMP7 | SwissTargetPrediction |
| wilforine | MMP12 | SwissTargetPrediction |
| wilforine | PSMB5 | SwissTargetPrediction |
| wilforine | BACE1 | SwissTargetPrediction |
| wilforine | PRKCE | SwissTargetPrediction |
| wilforine | RASGRP1 | SwissTargetPrediction |
| wilforine | F10 | SwissTargetPrediction |
| wilforine | CASP8 | SwissTargetPrediction |
| wilforine | CASP1 | SwissTargetPrediction |
| wilforine | CFD | SwissTargetPrediction |
| wilforine | AURKB | SwissTargetPrediction |
| wilforine | TACR2 | SwissTargetPrediction |
| wilforine | TACR1 | SwissTargetPrediction |
| wilforine | AURKA | SwissTargetPrediction |
| wilforine | PDE5A | SwissTargetPrediction |
| wilforine | HDAC1 | SwissTargetPrediction |
| wilforine | ADAM10 | SwissTargetPrediction |
| wilforine | CAPN1 | SwissTargetPrediction |
| wilforine | PAK4 | SwissTargetPrediction |
| wilforine | PAK1 | SwissTargetPrediction |
| wilforine | CDC7 | SwissTargetPrediction |
| wilforine | CHRM4 | SwissTargetPrediction |
| wilforine | CHRM5 | SwissTargetPrediction |
| wilforine | CHRM2 | SwissTargetPrediction |
| wilforine | CHRM1 | SwissTargetPrediction |
| wilforine | CHRM3 | SwissTargetPrediction |
| wilforine | GCK | SwissTargetPrediction |
| wilforine | CMA1 | SwissTargetPrediction |
| wilforine | CTSG | SwissTargetPrediction |
| wilforine | ADORA1 | SwissTargetPrediction |
| wilforine | ADORA2B | SwissTargetPrediction |
| wilforine | CTSK | SwissTargetPrediction |
| wilforine | CAPN2 | SwissTargetPrediction |
| wilforine | NOS2 | SwissTargetPrediction |
| wilforine | HDAC6 | SwissTargetPrediction |
| wilforine | NCOR2 HDAC3 | SwissTargetPrediction |
| wilforine | LIPC | SwissTargetPrediction |
| wilforine | DRD2 | SwissTargetPrediction |
| wilforine | DRD3 | SwissTargetPrediction |
| wilforine | HDAC8 | SwissTargetPrediction |
| wilforine | LIPG | SwissTargetPrediction |
| wilforine | CASP3 | SwissTargetPrediction |
| wilforine | CASP6 | SwissTargetPrediction |
| wilforine | CASP7 | SwissTargetPrediction |
| wilforine | MMP3 | SwissTargetPrediction |
| wilforine | MMP9 | SwissTargetPrediction |
| wilforine | AR | SwissTargetPrediction |
| wilforine | TYMS | SwissTargetPrediction |
| wilforine | LIPE | SwissTargetPrediction |
| wilforine | AGTR1 | SwissTargetPrediction |
| wilforine | PIK3CB | SwissTargetPrediction |
| wilforine | MET | SwissTargetPrediction |
| wilforine | SEM1 PSMD8 PSMD4 PSMD13 PSMD7 PSMD6 PSMD11 PSMD12 PSMD3 PSMD2 PSMC5 PSMC3 PSMC6 PSMC4 PSMC1 PSMC2 ADRM1 PSMD14 PSMD1 PSMB7 PSMB4 PSMB3 PSMB11 PSMB10 PSMA8 PSMA7 PSMA6 PSMA5 PSMA4 PSMA3 PSMA2 PSMA1 PSMB6 PSMB9 PSMB8 PSMB5 PSMB1 PSMB2 | SwissTargetPrediction |
| wilforine | TRPA1 | SwissTargetPrediction |
| wilforine | MCL1 | SwissTargetPrediction |
| wilforine | BCL2L1 | SwissTargetPrediction |
| wilforine | BCL2 | SwissTargetPrediction |
| wilforine | EDNRB | SwissTargetPrediction |
| wilforine | PTAFR | SwissTargetPrediction |
| wilforine | FNTA FNTB | SwissTargetPrediction |
| wilforine | MTOR | SwissTargetPrediction |
| wilforine | PIK3CA | SwissTargetPrediction |
| wilforine | BRAF | SwissTargetPrediction |
| wilforine | JAK3 | SwissTargetPrediction |
| wilforine | MAPK8 | SwissTargetPrediction |
| wilforine | CCR1 | SwissTargetPrediction |
| wilforine | CXCR2 | SwissTargetPrediction |
| wilforine | JAK1 | SwissTargetPrediction |
| wilforine | JAK2 | SwissTargetPrediction |
| wilforine | MMP2 | SwissTargetPrediction |
| wilforine | TYK2 | SwissTargetPrediction |
| wilforine | MMP14 | SwissTargetPrediction |
| wilforine | MMP8 | SwissTargetPrediction |
| wilforine | PDK1 | SwissTargetPrediction |
| wilforine | CDK5R1 CDK5 | SwissTargetPrediction |
| wilforine | CCNE1 CDK2 | SwissTargetPrediction |
| wilforine | PSMB9 | SwissTargetPrediction |
| wilforine | CDK9 CCNT1 | SwissTargetPrediction |
| wilforine | PIK3CG | SwissTargetPrediction |
| wilforine | BDKRB1 | SwissTargetPrediction |
| wilforine | LIMK2 | SwissTargetPrediction |
| wilforine | ADCY1 | SwissTargetPrediction |
| celastrol | TLR9 | SwissTargetPrediction |
| celastrol | PTPN11 | SwissTargetPrediction |
| celastrol | AKR1B1 | TCMSP |
| celastrol | TERT | SwissTargetPrediction |
| celastrol | HSF1 | SwissTargetPrediction |
| celastrol | CYP19A1 | SwissTargetPrediction |
| celastrol | PLA2G1B | SwissTargetPrediction |
| celastrol | CDC25B | SwissTargetPrediction |
| celastrol | PTPRF | SwissTargetPrediction |
| celastrol | ACP1 | SwissTargetPrediction |
| celastrol | PDE4D | SwissTargetPrediction |
| celastrol | SHBG | SwissTargetPrediction |
| celastrol | ALOX5AP | SwissTargetPrediction |
| celastrol | MPEG1 | SwissTargetPrediction |
| celastrol | KAT2B | SwissTargetPrediction |
| celastrol | NR3C2 | SwissTargetPrediction |
| celastrol | AR | SwissTargetPrediction |
| celastrol | NR1H4 | SwissTargetPrediction |
| celastrol | PLA2G4A | SwissTargetPrediction |
| celastrol | PLCG1 | SwissTargetPrediction |
| celastrol | PTGDR | SwissTargetPrediction |
| celastrol | TBXA2R | SwissTargetPrediction |
| celastrol | TRPA1 | SwissTargetPrediction |
| celastrol | EDNRA | SwissTargetPrediction |
| celastrol | SLC10A1 | SwissTargetPrediction |
| celastrol | MCL1 | SwissTargetPrediction |
| celastrol | MDM2 | SwissTargetPrediction |
| celastrol | AGTR1 | SwissTargetPrediction |
| celastrol | SLC10A2 | SwissTargetPrediction |
| celastrol | UBLCP1 | SwissTargetPrediction |
| celastrol | GPBAR1 | SwissTargetPrediction |
| celastrol | CD81 | SwissTargetPrediction |
| celastrol | IKBKB | SwissTargetPrediction |
| celastrol | PTGDR2 | SwissTargetPrediction |
| celastrol | PTPN1 | SwissTargetPrediction |
| celastrol | MMP3 | SwissTargetPrediction |
| celastrol | MMP1 | TCMSP |
| celastrol | MMP2 | TCMSP |
| celastrol | EPHA2 | SwissTargetPrediction |
| celastrol | ACE | SwissTargetPrediction |
| celastrol | PPARG | SwissTargetPrediction |
| celastrol | PGR | SwissTargetPrediction |
| celastrol | CCKBR | SwissTargetPrediction |
| celastrol | SAE1 UBA2 | SwissTargetPrediction |
| celastrol | NR3C1 | TCMSP |
| celastrol | ITGA2B ITGB3 | SwissTargetPrediction |
| celastrol | TYMS | SwissTargetPrediction |
| celastrol | THRA | SwissTargetPrediction |
| celastrol | THRB | SwissTargetPrediction |
| celastrol | HDAC6 | SwissTargetPrediction |
| celastrol | HDAC1 | SwissTargetPrediction |
| celastrol | F2 | SwissTargetPrediction |
| celastrol | POLB | SwissTargetPrediction |
| celastrol | OPRM1 | SwissTargetPrediction |
| celastrol | OPRD1 | SwissTargetPrediction |
| celastrol | OPRK1 | SwissTargetPrediction |
| celastrol | PTGFR | SwissTargetPrediction |
| celastrol | ALOX5 | SwissTargetPrediction |
| celastrol | HCAR2 | SwissTargetPrediction |
| celastrol | SRD5A2 | SwissTargetPrediction |
| celastrol | AKR1C3 | SwissTargetPrediction |
| celastrol | ITGB1 ITGA4 | SwissTargetPrediction |
| celastrol | CES2 | SwissTargetPrediction |
| celastrol | ITGAV ITGB3 | SwissTargetPrediction |
| celastrol | BMP1 | SwissTargetPrediction |
| celastrol | TRPM8 | SwissTargetPrediction |
| celastrol | PTGER3 | SwissTargetPrediction |
| celastrol | CYSLTR1 | SwissTargetPrediction |
| celastrol | XIAP | SwissTargetPrediction |
| celastrol | TBXAS1 | SwissTargetPrediction |
| celastrol | MAPKAPK2 | SwissTargetPrediction |
| celastrol | REN | SwissTargetPrediction |
| celastrol | PRKCA | SwissTargetPrediction |
| celastrol | RASGRP3 | SwissTargetPrediction |
| celastrol | F10 | SwissTargetPrediction |
| celastrol | ATP12A | SwissTargetPrediction |
| celastrol | ALDH2 | SwissTargetPrediction |
| celastrol | CYP17A1 | SwissTargetPrediction |
| celastrol | ST3GAL1 | SwissTargetPrediction |
| celastrol | CMA1 | SwissTargetPrediction |
| celastrol | EDNRB | SwissTargetPrediction |
| celastrol | CALCRL | SwissTargetPrediction |
| celastrol | STS | SwissTargetPrediction |
| celastrol | NR1H2 | SwissTargetPrediction |
| celastrol | S1PR2 | SwissTargetPrediction |
| celastrol | ITGA4 | SwissTargetPrediction |
| celastrol | RCE1 | SwissTargetPrediction |
| celastrol | CPB2 | SwissTargetPrediction |
| celastrol | F9 | SwissTargetPrediction |
| celastrol | DPP4 | SwissTargetPrediction |
| celastrol | HSD11B1 | SwissTargetPrediction |
| celastrol | PGGT1B FNTA | SwissTargetPrediction |
| celastrol | TUBB1 | SwissTargetPrediction |
| celastrol | JAK3 | SwissTargetPrediction |
| celastrol | JAK1 | SwissTargetPrediction |
| celastrol | JAK2 | SwissTargetPrediction |
| celastrol | CTSS | SwissTargetPrediction |
| celastrol | CTSA | SwissTargetPrediction |
| celastrol | ALOX12 | SwissTargetPrediction |
| celastrol | IL6 | SwissTargetPrediction |
| celastrol | VEGFA | TCMSP |
| celastrol | CCND1 | TCMSP |
| celastrol | BCL2 | TCMSP |
| celastrol | BCL2L1 | TCMSP |
| celastrol | BAX | TCMSP |
| celastrol | FLT1 | TCMSP |
| celastrol | KDR | TCMSP |
| celastrol | MMP9 | TCMSP |
| celastrol | JUN | TCMSP |
| celastrol | PTGS2 | TCMSP |
| celastrol | BIRC5 | TCMSP |
| celastrol | BIRC2 | TCMSP |
| celastrol | CFLAR | TCMSP |
| celastrol | CDC37 | TCMSP |
| celastrol | TIMP1 | TCMSP |
| celastrol | TIMP2 | TCMSP |
| celastrol | MYC | TCMSP |
| celastrol | FN1 | TCMSP |
| celastrol | COL7A1 | TCMSP |
| celastrol | COL4A4 | TCMSP |

Table S4. Target prediction of RA.

| Disease | Target | Origin |
| --- | --- | --- |
| RA | KRAS | GeneCards |
| RA | HRAS | GeneCards |
| RA | NRAS | GeneCards |
| RA | RAF1 | GeneCards |
| RA | BRAF | GeneCards |
| RA | SOS1 | GeneCards |
| RA | NF1 | GeneCards |
| RA | RRAS | GeneCards |
| RA | RASA2 | GeneCards |
| RA | EGFR | GeneCards |
| RA | PIK3CA | GeneCards |
| RA | RAP1A | GeneCards |
| RA | RASA1 | GeneCards |
| RA | TP53 | GeneCards |
| RA | MAP2K1 | GeneCards |
| RA | RALA | GeneCards |
| RA | MRAS | GeneCards |
| RA | IL6 | GeneCards |
| RA | AKT1 | GeneCards |
| RA | RRAS2 | GeneCards |
| RA | RASGRF1 | GeneCards |
| RA | SRC | GeneCards |
| RA | RASGRP1 | GeneCards |
| RA | SOS2 | GeneCards |
| RA | MAPK1 | GeneCards |
| RA | RAC1 | GeneCards |
| RA | RASGRF2 | GeneCards |
| RA | SHOC2 | GeneCards |
| RA | CDKN2A | GeneCards |
| RA | RASSF5 | GeneCards |
| RA | PTPN22 | GeneCards |
| RA | KIT | GeneCards |
| RA | GRB2 | GeneCards |
| RA | RASSF1 | GeneCards |
| RA | FGFR2 | GeneCards |
| RA | RET | GeneCards |
| RA | PTPN11 | GeneCards |
| RA | FGFR1 | GeneCards |
| RA | ERBB2 | GeneCards |
| RA | PTEN | GeneCards |
| RA | IL10 | GeneCards |
| RA | RIT1 | GeneCards |
| RA | FASLG | GeneCards |
| RA | SHC1 | GeneCards |
| RA | RALB | GeneCards |
| RA | MAP2K2 | GeneCards |
| RA | CTNNB1 | GeneCards |
| RA | RHOA | GeneCards |
| RA | RALGDS | GeneCards |
| RA | CCND1 | GeneCards |
| RA | PADI4 | GeneCards |
| RA | SYNGAP1 | GeneCards |
| RA | NTRK1 | GeneCards |
| RA | CDH1 | GeneCards |
| RA | NFKBIA | GeneCards |
| RA | MTOR | GeneCards |
| RA | PDGFRB | GeneCards |
| RA | EGF | GeneCards |
| RA | RASA4 | GeneCards |
| RA | MAPK8 | GeneCards |
| RA | RIN1 | GeneCards |
| RA | LZTR1 | GeneCards |
| RA | RB1 | GeneCards |
| RA | JUN | GeneCards |
| RA | RAC2 | GeneCards |
| RA | MIR155 | GeneCards |
| RA | FGFR3 | GeneCards |
| RA | CDK4 | GeneCards |
| RA | MIR146A | GeneCards |
| RA | RASA3 | GeneCards |
| RA | RASGRP4 | GeneCards |
| RA | MAPK3 | GeneCards |
| RA | TERT | GeneCards |
| RA | RAP1B | GeneCards |
| RA | RASAL1 | GeneCards |
| RA | MIR150 | GeneCards |
| RA | MYC | GeneCards |
| RA | APC | GeneCards |
| RA | FOS | GeneCards |
| RA | RASGRP3 | GeneCards |
| RA | SMAD4 | GeneCards |
| RA | SLC22A4 | GeneCards |
| RA | CASP8 | GeneCards |
| RA | RREB1 | GeneCards |
| RA | TNF | GeneCards |
| RA | RASAL2 | GeneCards |
| RA | PIK3CG | GeneCards |
| RA | RAPGEF2 | GeneCards |
| RA | PLA2G2A | GeneCards |
| RA | VEGFA | GeneCards |
| RA | CDKN1A | GeneCards |
| RA | RASGRP2 | GeneCards |
| RA | FLT3 | GeneCards |
| RA | ERAS | GeneCards |
| RA | MIR21 | GeneCards |
| RA | JAK2 | GeneCards |
| RA | MLH1 | GeneCards |
| RA | MAP3K8 | GeneCards |
| RA | NFKBIL1 | GeneCards |
| RA | BRCA1 | GeneCards |
| RA | BAX | GeneCards |
| RA | RASAL3 | GeneCards |
| RA | RAC3 | GeneCards |
| RA | ITGB2 | GeneCards |
| RA | HLA-DRB1 | GeneCards |
| RA | MIR145 | GeneCards |
| RA | SPRED1 | GeneCards |
| RA | CDC42 | GeneCards |
| RA | ATM | GeneCards |
| RA | MIR34A | GeneCards |
| RA | KSR1 | GeneCards |
| RA | U2AF1 | GeneCards |
| RA | TGFB1 | GeneCards |
| RA | MIR132 | GeneCards |
| RA | MIR125A | GeneCards |
| RA | BRCA2 | GeneCards |
| RA | TLR2 | GeneCards |
| RA | IGF1R | GeneCards |
| RA | MIR31 | GeneCards |
| RA | RAP2B | GeneCards |
| RA | FGF2 | GeneCards |
| RA | ARAF | GeneCards |
| RA | MIR17 | GeneCards |
| RA | PPARG | GeneCards |
| RA | MAPK10 | GeneCards |
| RA | RARA | GeneCards |
| RA | DAB2IP | GeneCards |
| RA | MET | GeneCards |
| RA | CD44 | GeneCards |
| RA | MIR126 | GeneCards |
| RA | RAP2A | GeneCards |
| RA | ALK | GeneCards |
| RA | MAPK14 | GeneCards |
| RA | MIR143 | GeneCards |
| RA | IL2 | GeneCards |
| RA | ESR1 | GeneCards |
| RA | RERG | GeneCards |
| RA | RAB5C | GeneCards |
| RA | RHEB | GeneCards |
| RA | EP300 | GeneCards |
| RA | CDK2 | GeneCards |
| RA | RAB5A | GeneCards |
| RA | PIK3CB | GeneCards |
| RA | RSU1 | GeneCards |
| RA | RASSF2 | GeneCards |
| RA | RHOB | GeneCards |
| RA | RAN | GeneCards |
| RA | CIITA | GeneCards |
| RA | GATA2 | GeneCards |
| RA | MIR127 | GeneCards |
| RA | IGF1 | GeneCards |
| RA | CD244 | GeneCards |
| RA | IL1B | GeneCards |
| RA | CXCL8 | GeneCards |
| RA | RAD51 | GeneCards |
| RA | RASL11A | GeneCards |
| RA | STAT3 | GeneCards |
| RA | RIT2 | GeneCards |
| RA | MMP9 | GeneCards |
| RA | CBL | GeneCards |
| RA | AURKA | GeneCards |
| RA | RAB5B | GeneCards |
| RA | MIR221 | GeneCards |
| RA | MIR27A | GeneCards |
| RA | MIR222 | GeneCards |
| RA | RARB | GeneCards |
| RA | RHOD | GeneCards |
| RA | STK11 | GeneCards |
| RA | MIR15A | GeneCards |
| RA | MIR141 | GeneCards |
| RA | CASP3 | GeneCards |
| RA | NKIRAS2 | GeneCards |
| RA | KDR | GeneCards |
| RA | NKIRAS1 | GeneCards |
| RA | PTGS2 | GeneCards |
| RA | RHOC | GeneCards |
| RA | MIRLET7A1 | GeneCards |
| RA | MIR205 | GeneCards |
| RA | MIR182 | GeneCards |
| RA | RIN2 | GeneCards |
| RA | KSR2 | GeneCards |
| RA | RASSF6 | GeneCards |
| RA | RAB8A | GeneCards |
| RA | CSF1 | GeneCards |
| RA | MSH2 | GeneCards |
| RA | ANXA5 | GeneCards |
| RA | MIR200A | GeneCards |
| RA | MMP2 | GeneCards |
| RA | FAS | GeneCards |
| RA | RASD1 | GeneCards |
| RA | HGF | GeneCards |
| RA | RGL2 | GeneCards |
| RA | RAB25 | GeneCards |
| RA | RAB27A | GeneCards |
| RA | PIK3R1 | GeneCards |
| RA | MIR483 | GeneCards |
| RA | RAB11A | GeneCards |
| RA | BCL2L1 | GeneCards |
| RA | CRP | GeneCards |
| RA | MIR34C | GeneCards |
| RA | CSF2 | GeneCards |
| RA | ASXL1 | GeneCards |
| RA | CNKSR1 | GeneCards |
| RA | MIR34B | GeneCards |
| RA | MIR10A | GeneCards |
| RA | MIR20A | GeneCards |
| RA | RAP2C | GeneCards |
| RA | MIR214 | GeneCards |
| RA | MIR200C | GeneCards |
| RA | ESR2 | GeneCards |
| RA | BCL2 | GeneCards |
| RA | PLCE1 | GeneCards |
| RA | MIR96 | GeneCards |
| RA | MIR128-2 | GeneCards |
| RA | MIR183 | GeneCards |
| RA | CHEK2 | GeneCards |
| RA | MIR203A | GeneCards |
| RA | MIR210 | GeneCards |
| RA | CXCL12 | GeneCards |
| RA | IKBKB | GeneCards |
| RA | PLCG1 | GeneCards |
| RA | FLT1 | GeneCards |
| RA | RAB3A | GeneCards |
| RA | MIR148A | GeneCards |
| RA | CSF1R | GeneCards |
| RA | NGF | GeneCards |
| RA | MUC1 | GeneCards |
| RA | IL2RA | GeneCards |
| RA | RAPH1 | GeneCards |
| RA | STAT1 | GeneCards |
| RA | IDH2 | GeneCards |
| RA | MIR100 | GeneCards |
| RA | IFNG | GeneCards |
| RA | RAPGEF5 | GeneCards |
| RA | MIR25 | GeneCards |
| RA | RAB11B | GeneCards |
| RA | MIR32 | GeneCards |
| RA | MIRLET7B | GeneCards |
| RA | MAP2K4 | GeneCards |
| RA | TIMP1 | GeneCards |
| RA | GNAS | GeneCards |
| RA | RAB7A | GeneCards |
| RA | HIF1A | GeneCards |
| RA | RAB10 | GeneCards |
| RA | PTK2 | GeneCards |
| RA | CDKN1B | GeneCards |
| RA | IRF1 | GeneCards |
| RA | RASIP1 | GeneCards |
| RA | RAB13 | GeneCards |
| RA | IQGAP1 | GeneCards |
| RA | PRKD1 | GeneCards |
| RA | ANGPT1 | GeneCards |
| RA | MIR106B | GeneCards |
| RA | MIR335 | GeneCards |
| RA | TGFA | GeneCards |
| RA | NOTCH1 | GeneCards |
| RA | RAB9B | GeneCards |
| RA | IL2RB | GeneCards |
| RA | TET2 | GeneCards |
| RA | IL4 | GeneCards |
| RA | RAB35 | GeneCards |
| RA | CNKSR2 | GeneCards |
| RA | PRKCA | GeneCards |
| RA | MMP1 | GeneCards |
| RA | RIN3 | GeneCards |
| RA | RAB23 | GeneCards |
| RA | STAT4 | GeneCards |
| RA | MDM2 | GeneCards |
| RA | RABL3 | GeneCards |
| RA | TEK | GeneCards |
| RA | RAB31 | GeneCards |
| RA | RAPGEF6 | GeneCards |
| RA | MIR18A | GeneCards |
| RA | RAB36 | GeneCards |
| RA | ERCC6 | GeneCards |
| RA | CXCR4 | GeneCards |
| RA | FN1 | GeneCards |
| RA | MIRLET7D | GeneCards |
| RA | IDH1 | GeneCards |
| RA | TIMP2 | GeneCards |
| RA | IL17A | GeneCards |
| RA | MIR140 | GeneCards |
| RA | CCNA2 | GeneCards |
| RA | CRABP2 | GeneCards |
| RA | RRAD | GeneCards |
| RA | IQGAP3 | GeneCards |
| RA | KMT2A | GeneCards |
| RA | RAB3B | GeneCards |
| RA | RAB2A | GeneCards |
| RA | RAB33A | GeneCards |
| RA | MIR93 | GeneCards |
| RA | MIR181A2 | GeneCards |
| RA | MIR107 | GeneCards |
| RA | DNMT1 | GeneCards |
| RA | NFE2L2 | GeneCards |
| RA | RAB14 | GeneCards |
| RA | RXRA | GeneCards |
| RA | MMP3 | GeneCards |
| RA | ABCB1 | GeneCards |
| RA | HDAC9 | GeneCards |
| RA | MIR10B | GeneCards |
| RA | MIR19A | GeneCards |
| RA | GDNF | GeneCards |
| RA | CAV1 | GeneCards |
| RA | RAB4A | GeneCards |
| RA | MIR199B | GeneCards |
| RA | CD4 | GeneCards |
| RA | PDGFRA | GeneCards |
| RA | MALAT1 | GeneCards |
| RA | PIK3CD | GeneCards |
| RA | RASSF3 | GeneCards |
| RA | MIR451A | GeneCards |
| RA | SETBP1 | GeneCards |
| RA | GREM1 | GeneCards |
| RA | ITGAM | GeneCards |
| RA | RASL12 | GeneCards |
| RA | MIR200B | GeneCards |
| RA | NPM1 | GeneCards |
| RA | RASL11B | GeneCards |
| RA | ITGA8 | GeneCards |
| RA | RUNX1 | GeneCards |
| RA | RASL10A | GeneCards |
| RA | RAB1A | GeneCards |
| RA | RAB6A | GeneCards |
| RA | TMCO1 | GeneCards |
| RA | NFKB1 | GeneCards |
| RA | RASGEF1A | GeneCards |
| RA | MIR204 | GeneCards |
| RA | RAPGEF1 | GeneCards |
| RA | RAB30 | GeneCards |
| RA | ITGB1 | GeneCards |
| RA | MIR378A | GeneCards |
| RA | TGFB2 | GeneCards |
| RA | DDR2 | GeneCards |
| RA | AREG | GeneCards |
| RA | MIR499A | GeneCards |
| RA | MIR30E | GeneCards |
| RA | KITLG | GeneCards |
| RA | DNMT3A | GeneCards |
| RA | CTLA4 | GeneCards |
| RA | ODC1 | GeneCards |
| RA | RAB28 | GeneCards |
| RA | G3BP1 | GeneCards |
| RA | RXRG | GeneCards |
| RA | CYP26A1 | GeneCards |
| RA | H19 | GeneCards |
| RA | CTSB | GeneCards |
| RA | PPP2R1B | GeneCards |
| RA | RELA | GeneCards |
| RA | RHOH | GeneCards |
| RA | ALB | GeneCards |
| RA | MIR29A | GeneCards |
| RA | DIRAS1 | GeneCards |
| RA | RAB4B | GeneCards |
| RA | RAB18 | GeneCards |
| RA | SPP1 | GeneCards |
| RA | RASEF | GeneCards |
| RA | JAK1 | GeneCards |
| RA | INS | GeneCards |
| RA | TNFRSF1B | GeneCards |
| RA | CD79A | GeneCards |
| RA | RASA4B | GeneCards |
| RA | HOTAIR | GeneCards |
| RA | RCE1 | GeneCards |
| RA | ALDH1A2 | GeneCards |
| RA | CCL2 | GeneCards |
| RA | MIRLET7C | GeneCards |
| RA | TIAM1 | GeneCards |
| RA | MEN1 | GeneCards |
| RA | RASSF7 | GeneCards |
| RA | CCR6 | GeneCards |
| RA | SPRY4 | GeneCards |
| RA | MSH3 | GeneCards |
| RA | RASSF8 | GeneCards |
| RA | PTCH1 | GeneCards |
| RA | ALDH1A1 | GeneCards |
| RA | TNFRSF1A | GeneCards |
| RA | MIR372 | GeneCards |
| RA | MIRLET7E | GeneCards |
| RA | MIR342 | GeneCards |
| RA | MIR29C | GeneCards |
| RA | MIR324 | GeneCards |
| RA | RAB34 | GeneCards |
| RA | MIF | GeneCards |
| RA | CAT | GeneCards |
| RA | RHOV | GeneCards |
| RA | CASP9 | GeneCards |
| RA | RAB29 | GeneCards |
| RA | RXRB | GeneCards |
| RA | RRAGB | GeneCards |
| RA | NOS2 | GeneCards |
| RA | RASSF4 | GeneCards |
| RA | NTRK2 | GeneCards |
| RA | CSF3 | GeneCards |
| RA | IL1RN | GeneCards |
| RA | UCA1 | GeneCards |
| RA | INSR | GeneCards |
| RA | NBN | GeneCards |
| RA | RAB3C | GeneCards |
| RA | RALBP1 | GeneCards |
| RA | DIRAS2 | GeneCards |
| RA | FLT4 | GeneCards |
| RA | MAP2K7 | GeneCards |
| RA | AXIN2 | GeneCards |
| RA | RARS1 | GeneCards |
| RA | ICAM1 | GeneCards |
| RA | IGFBP3 | GeneCards |
| RA | IGF2 | GeneCards |
| RA | RHOG | GeneCards |
| RA | CD274 | GeneCards |
| RA | RAB24 | GeneCards |
| RA | PDCD1 | GeneCards |
| RA | BMP4 | GeneCards |
| RA | ITGAL | GeneCards |
| RA | RHOJ | GeneCards |
| RA | BAD | GeneCards |
| RA | RAB1B | GeneCards |
| RA | BIRC5 | GeneCards |
| RA | MCL1 | GeneCards |
| RA | MPO | GeneCards |
| RA | MIR26A1 | GeneCards |
| RA | RASGEF1B | GeneCards |
| RA | ARHGEF2 | GeneCards |
| RA | BDNF | GeneCards |
| RA | SERPINA3 | GeneCards |
| RA | AKT3 | GeneCards |
| RA | AKT2 | GeneCards |
| RA | DIRAS3 | GeneCards |
| RA | ERBB3 | GeneCards |
| RA | MKI67 | GeneCards |
| RA | FNTB | GeneCards |
| RA | IL3 | GeneCards |
| RA | GSTP1 | GeneCards |
| RA | ENO1 | GeneCards |
| RA | CREB1 | GeneCards |
| RA | MIR30D | GeneCards |
| RA | MIR486-1 | GeneCards |
| RA | MSH6 | GeneCards |
| RA | RAB22A | GeneCards |
| RA | FGF10 | GeneCards |
| RA | MMP13 | GeneCards |
| RA | VEGFC | GeneCards |
| RA | CD40 | GeneCards |
| RA | GAPDH | GeneCards |
| RA | RAB27B | GeneCards |
| RA | RAB40C | GeneCards |
| RA | REL | GeneCards |
| RA | SLC2A1 | GeneCards |
| RA | RAB33B | GeneCards |
| RA | CHUK | GeneCards |
| RA | EGR1 | GeneCards |
| RA | PTHLH | GeneCards |
| RA | RASL10B | GeneCards |
| RA | MIR133B | GeneCards |
| RA | CYP2A6 | GeneCards |
| RA | RPS6KB1 | GeneCards |
| RA | CTSL | GeneCards |
| RA | IL1A | GeneCards |
| RA | FCRL3 | GeneCards |
| RA | FGFR4 | GeneCards |
| RA | TNFRSF10B | GeneCards |
| RA | RASSF9 | GeneCards |
| RA | RAB9A | GeneCards |
| RA | EZH2 | GeneCards |
| RA | CDK6 | GeneCards |
| RA | HLA-DQB1 | GeneCards |
| RA | SMO | GeneCards |
| RA | A2ML1 | GeneCards |
| RA | NCAM1 | GeneCards |
| RA | RAB3D | GeneCards |
| RA | THBS1 | GeneCards |
| RA | PLAU | GeneCards |
| RA | HSP90AA1 | GeneCards |
| RA | MIR130A | GeneCards |
| RA | FCGR3A | GeneCards |
| RA | TNFSF11 | GeneCards |
| RA | IFNA1 | GeneCards |
| RA | RAB39A | GeneCards |
| RA | ACP5 | GeneCards |
| RA | IL7 | GeneCards |
| RA | ETV6 | GeneCards |
| RA | TFRC | GeneCards |
| RA | TNFSF10 | GeneCards |
| RA | PARP1 | GeneCards |
| RA | IFNB1 | GeneCards |
| RA | HLA-DPB1 | GeneCards |
| RA | RAB32 | GeneCards |
| RA | AFDN | GeneCards |
| RA | MIR373 | GeneCards |
| RA | FHIT | GeneCards |
| RA | SELL | GeneCards |
| RA | GNAQ | GeneCards |
| RA | TYMS | GeneCards |
| RA | MIR192 | GeneCards |
| RA | IL6ST | GeneCards |
| RA | PRTN3 | GeneCards |
| RA | IL15 | GeneCards |
| RA | SP1 | GeneCards |
| RA | TNFRSF10A | GeneCards |
| RA | RAB2B | GeneCards |
| RA | RAB38 | GeneCards |
| RA | MIRLET7G | GeneCards |
| RA | CDK1 | GeneCards |
| RA | CYP1A1 | GeneCards |
| RA | LCK | GeneCards |
| RA | TLR4 | GeneCards |
| RA | XRCC3 | GeneCards |
| RA | BMP6 | GeneCards |
| RA | SRSF2 | GeneCards |
| RA | MIRLET7A3 | GeneCards |
| RA | DCK | GeneCards |
| RA | NME1 | GeneCards |
| RA | SPRY1 | GeneCards |
| RA | LOX | GeneCards |
| RA | TRAF1 | GeneCards |
| RA | MTHFR | GeneCards |
| RA | HLA-DMA | GeneCards |
| RA | CYCS | GeneCards |
| RA | MIR146B | GeneCards |
| RA | RRAGC | GeneCards |
| RA | PROM1 | GeneCards |
| RA | SERPINH1 | GeneCards |
| RA | APBB1IP | GeneCards |
| RA | SRF | GeneCards |
| RA | RAB21 | GeneCards |
| RA | CFLAR | GeneCards |
| RA | SPRED2 | GeneCards |
| RA | PLK1 | GeneCards |
| RA | MIR185 | GeneCards |
| RA | RAB26 | GeneCards |
| RA | XIAP | GeneCards |
| RA | NUP214 | GeneCards |
| RA | FNTA | GeneCards |
| RA | GFRA1 | GeneCards |
| RA | CD28 | GeneCards |
| RA | CD69 | GeneCards |
| RA | ABL1 | GeneCards |
| RA | HMGB1 | GeneCards |
| RA | CCR5 | GeneCards |
| RA | HLA-DQA1 | GeneCards |
| RA | TGFBR2 | GeneCards |
| RA | LRRK2 | GeneCards |
| RA | PML | GeneCards |
| RA | IL6R | GeneCards |
| RA | CYP26B1 | GeneCards |
| RA | IL11 | GeneCards |
| RA | COL2A1 | GeneCards |
| RA | MIR98 | GeneCards |
| RA | CCL3 | GeneCards |
| RA | CD80 | GeneCards |
| RA | EPOR | GeneCards |
| RA | OSM | GeneCards |
| RA | CCL5 | GeneCards |
| RA | RAB8B | GeneCards |
| RA | TRAF3 | GeneCards |
| RA | MIR193B | GeneCards |
| RA | RAB37 | GeneCards |
| RA | TNFRSF11B | GeneCards |
| RA | RHEBL1 | GeneCards |
| RA | TGFB3 | GeneCards |
| RA | RRAGA | GeneCards |
| RA | RAB6B | GeneCards |
| RA | MGMT | GeneCards |
| RA | SYK | GeneCards |
| RA | MIR137 | GeneCards |
| RA | ACAN | GeneCards |
| RA | PIK3R3 | GeneCards |
| RA | EPO | GeneCards |
| RA | HSPD1 | GeneCards |
| RA | IL18 | GeneCards |
| RA | PLA2G10 | GeneCards |
| RA | FGF8 | GeneCards |
| RA | VCAM1 | GeneCards |
| RA | CTNND1 | GeneCards |
| RA | HSPB1 | GeneCards |
| RA | IRF5 | GeneCards |
| RA | RAB39B | GeneCards |
| RA | BCL10 | GeneCards |
| RA | MIR30B | GeneCards |
| RA | BUB1 | GeneCards |
| RA | RAB42 | GeneCards |
| RA | RAB43 | GeneCards |
| RA | GNB1 | GeneCards |
| RA | GRP | GeneCards |
| RA | TLR9 | GeneCards |
| RA | SLC22A18 | GeneCards |
| RA | LIF | GeneCards |
| RA | HSPA5 | GeneCards |
| RA | CXCL1 | GeneCards |
| RA | NR3C1 | GeneCards |
| RA | MMP7 | GeneCards |
| RA | E2F1 | GeneCards |
| RA | MBL2 | GeneCards |
| RA | HLA-DMB | GeneCards |
| RA | IL32 | GeneCards |
| RA | PALB2 | GeneCards |
| RA | FCGR2A | GeneCards |
| RA | SNAI1 | GeneCards |
| RA | IL1R1 | GeneCards |
| RA | ABCC1 | GeneCards |
| RA | HLA-DRA | GeneCards |
| RA | RRAGD | GeneCards |
| RA | RHOQ | GeneCards |
| RA | MIR429 | GeneCards |
| RA | MIR502 | GeneCards |
| RA | CDH2 | GeneCards |
| RA | HLA-DQA2 | GeneCards |
| RA | ALOX5 | GeneCards |
| RA | FOXP3 | GeneCards |
| RA | EPHA2 | GeneCards |
| RA | TP63 | GeneCards |
| RA | RGL1 | GeneCards |
| RA | PXN | GeneCards |
| RA | KRT7 | GeneCards |
| RA | RAB17 | GeneCards |
| RA | RAB40B | GeneCards |
| RA | NRG1 | GeneCards |
| RA | IL13 | GeneCards |
| RA | CXCR5 | GeneCards |
| RA | PRKCB | GeneCards |
| RA | SELE | GeneCards |
| RA | VAV1 | GeneCards |
| RA | IL1RAPL2 | GeneCards |
| RA | ZFAS1 | GeneCards |
| RA | S100A9 | GeneCards |
| RA | PLAUR | GeneCards |
| RA | CTNNA1 | GeneCards |
| RA | RAB12 | GeneCards |
| RA | MICB | GeneCards |
| RA | HLA-DOA | GeneCards |
| RA | HLA-DRB5 | GeneCards |
| RA | MEG3 | GeneCards |
| RA | ELK1 | GeneCards |
| RA | ANXA1 | GeneCards |
| RA | SAA1 | GeneCards |
| RA | MAPK9 | GeneCards |
| RA | CD40LG | GeneCards |
| RA | PDPK1 | GeneCards |
| RA | CXCR3 | GeneCards |
| RA | BGLAP | GeneCards |
| RA | MST1R | GeneCards |
| RA | CEACAM5 | GeneCards |
| RA | PRKN | GeneCards |
| RA | FCGR2B | GeneCards |
| RA | SF3B1 | GeneCards |
| RA | MIR199A1 | GeneCards |
| RA | LGALS3 | GeneCards |
| RA | RASD2 | GeneCards |
| RA | BCL2L11 | GeneCards |
| RA | MIR30A | GeneCards |
| RA | ELANE | GeneCards |
| RA | CDKN2B | GeneCards |
| RA | GRAP | GeneCards |
| RA | CEBPA | GeneCards |
| RA | CD86 | GeneCards |
| RA | FCGR3B | GeneCards |
| RA | H2AC18 | GeneCards |
| RA | IL23A | GeneCards |
| RA | RABL6 | GeneCards |
| RA | MACIR | GeneCards |
| RA | RAB41 | GeneCards |
| RA | RERGL | GeneCards |
| RA | WRN | GeneCards |
| RA | ACVR1B | GeneCards |
| RA | CYP1B1 | GeneCards |
| RA | CR1 | GeneCards |
| RA | TTR | GeneCards |
| RA | RAB20 | GeneCards |
| RA | CDX2 | GeneCards |
| RA | KRT20 | GeneCards |
| RA | IRS1 | GeneCards |
| RA | RADIL | GeneCards |
| RA | TYMP | GeneCards |
| RA | ERBB4 | GeneCards |
| RA | SLC19A1 | GeneCards |
| RA | WT1 | GeneCards |
| RA | IL22 | GeneCards |
| RA | CXCL10 | GeneCards |
| RA | CDKN2B-AS1 | GeneCards |
| RA | KRT19 | GeneCards |
| RA | GSTM1 | GeneCards |
| RA | JAK3 | GeneCards |
| RA | DNMT3B | GeneCards |
| RA | KNDC1 | GeneCards |
| RA | TSC1 | GeneCards |
| RA | LTA | GeneCards |
| RA | TUG1 | GeneCards |
| RA | PTH | GeneCards |
| RA | CCNB1 | GeneCards |
| RA | ILK | GeneCards |
| RA | RAP1GAP | GeneCards |
| RA | CXCL2 | GeneCards |
| RA | CD82 | GeneCards |
| RA | RHOT1 | GeneCards |
| RA | STAT5A | GeneCards |
| RA | RHOT2 | GeneCards |
| RA | GDF15 | GeneCards |
| RA | LIG4 | GeneCards |
| RA | IL18R1 | GeneCards |
| RA | EIF4EBP1 | GeneCards |
| RA | STAT5B | GeneCards |
| RA | RAB15 | GeneCards |
| RA | RAB19 | GeneCards |
| RA | RAB44 | GeneCards |
| RA | PALLD | GeneCards |
| RA | DAPK1 | GeneCards |
| RA | SOD2 | GeneCards |
| RA | MAP2K3 | GeneCards |
| RA | CFTR | GeneCards |
| RA | COMP | GeneCards |
| RA | AXL | GeneCards |
| RA | MIR181A1 | GeneCards |
| RA | MUC5AC | GeneCards |
| RA | DMTF1 | GeneCards |
| RA | SOX2 | GeneCards |
| RA | GPI | GeneCards |
| RA | FGF1 | GeneCards |
| RA | MYB | GeneCards |
| RA | RAB7B | GeneCards |
| RA | CYP3A4 | GeneCards |
| RA | KMT2D | GeneCards |
| RA | MXRA5 | GeneCards |
| RA | MIR320A | GeneCards |
| RA | MIR197 | GeneCards |
| RA | CCAT1 | GeneCards |
| RA | RASSF10 | GeneCards |
| RA | TOP1 | GeneCards |
| RA | BMP2 | GeneCards |
| RA | NQO1 | GeneCards |
| RA | GADD45A | GeneCards |
| RA | TSC2 | GeneCards |
| RA | HMMR | GeneCards |
| RA | PAK1 | GeneCards |
| RA | SMAD2 | GeneCards |
| RA | JAM3 | GeneCards |
| RA | NR4A2 | GeneCards |
| RA | RTEL1 | GeneCards |
| RA | GAS5 | GeneCards |
| RA | CCL4 | GeneCards |
| RA | HP | GeneCards |
| RA | GGT1 | GeneCards |
| RA | TNFSF13B | GeneCards |
| RA | NAT2 | GeneCards |
| RA | CCR1 | GeneCards |
| RA | RHOF | GeneCards |
| RA | HDAC1 | GeneCards |
| RA | ITGA2B | GeneCards |
| RA | EPHB2 | GeneCards |
| RA | PIK3R2 | GeneCards |
| RA | MYCN | GeneCards |
| RA | KRT8 | GeneCards |
| RA | CX3CR1 | GeneCards |
| RA | YWHAE | GeneCards |
| RA | CCND2 | GeneCards |
| RA | RAB6C | GeneCards |
| RA | PCNA | GeneCards |
| RA | NEAT1 | GeneCards |
| RA | IL26 | GeneCards |
| RA | BAK1 | GeneCards |
| RA | APOH | GeneCards |
| RA | CDKN3 | GeneCards |
| RA | IL24 | GeneCards |
| RA | CTSK | GeneCards |
| RA | TNFRSF11A | GeneCards |
| RA | IL10RA | GeneCards |
| RA | CX3CL1 | GeneCards |
| RA | MIR142 | GeneCards |
| RA | PDGFB | GeneCards |
| RA | ADAMTS4 | GeneCards |
| RA | RELB | GeneCards |
| RA | CXCL6 | GeneCards |
| RA | PTGS1 | GeneCards |
| RA | AURKB | GeneCards |
| RA | CTSG | GeneCards |
| RA | PMS2 | GeneCards |
| RA | HSF1 | GeneCards |
| RA | NGFR | GeneCards |
| RA | NKX2-1 | GeneCards |
| RA | SYVN1 | GeneCards |
| RA | CDK5 | GeneCards |
| RA | PTK2B | GeneCards |
| RA | BCR | GeneCards |
| RA | CYP2E1 | GeneCards |
| RA | SLC29A1 | GeneCards |
| RA | PVT1 | GeneCards |
| RA | MECOM | GeneCards |
| RA | CD247 | GeneCards |
| RA | PLB1 | GeneCards |
| RA | EZR | GeneCards |
| RA | MAP3K1 | GeneCards |
| RA | PTPRC | GeneCards |
| RA | RAB40A | GeneCards |
| RA | MPL | GeneCards |
| RA | LECT2 | GeneCards |
| RA | ICMT | GeneCards |
| RA | GNRH1 | GeneCards |
| RA | RABL2A | GeneCards |
| RA | HBEGF | GeneCards |
| RA | S100A12 | GeneCards |
| RA | PPP1CC | GeneCards |
| RA | IL16 | GeneCards |
| RA | FLG | GeneCards |
| RA | PAX8 | GeneCards |
| RA | ITGA5 | GeneCards |
| RA | TNFRSF13C | GeneCards |
| RA | MMP12 | GeneCards |
| RA | CCR7 | GeneCards |
| RA | KLF4 | GeneCards |
| RA | ADAM17 | GeneCards |
| RA | CHI3L1 | GeneCards |
| RA | XRCC1 | GeneCards |
| RA | CRABP1 | GeneCards |
| RA | STK4 | GeneCards |
| RA | PRRC2A | GeneCards |
| RA | IL19 | GeneCards |
| RA | HLA-B | GeneCards |
| RA | SMAD3 | GeneCards |
| RA | CCN2 | GeneCards |
| RA | MIR196A1 | GeneCards |
| RA | SERPINE1 | GeneCards |
| RA | CCR2 | GeneCards |
| RA | CCL20 | GeneCards |
| RA | SLC11A1 | GeneCards |
| RA | ABCG2 | GeneCards |
| RA | FOXO3 | GeneCards |
| RA | IL23R | GeneCards |
| RA | ETS1 | GeneCards |
| RA | RETN | GeneCards |
| RA | CAST | GeneCards |
| RA | RARG | GeneCards |
| RA | RHOU | GeneCards |
| RA | IFI27 | GeneCards |
| RA | AGTR2 | GeneCards |
| RA | FSTL1 | GeneCards |
| RA | PPP1CB | GeneCards |
| RA | PTPN3 | GeneCards |
| RA | SAA4 | GeneCards |
| RA | FBXW7 | GeneCards |
| RA | XIST | GeneCards |
| RA | TNFSF13 | GeneCards |
| RA | NRP1 | GeneCards |
| RA | IQGAP2 | GeneCards |
| RA | IL37 | GeneCards |
| RA | ANGPT2 | GeneCards |
| RA | TP73 | GeneCards |
| RA | CREBBP | GeneCards |
| RA | MUC4 | GeneCards |
| RA | MMP8 | GeneCards |
| RA | CXCL5 | GeneCards |
| RA | MAFB | GeneCards |
| RA | ARHGAP5 | GeneCards |
| RA | MIR195 | GeneCards |
| RA | VEGFD | GeneCards |
| RA | PRKCG | GeneCards |
| RA | CCKBR | GeneCards |
| RA | DDX39B | GeneCards |
| RA | EPCAM | GeneCards |
| RA | BRAP | GeneCards |
| RA | B2M | GeneCards |
| RA | KRT18 | GeneCards |
| RA | REM2 | GeneCards |
| RA | TERC | GeneCards |
| RA | IL17RA | GeneCards |
| RA | PRKACA | GeneCards |
| RA | SIRT1 | GeneCards |
| RA | RBP4 | GeneCards |
| RA | ITGA6 | GeneCards |
| RA | CXCL9 | GeneCards |
| RA | ARF6 | GeneCards |
| RA | IL18BP | GeneCards |
| RA | TAGAP | GeneCards |
| RA | MIR223 | GeneCards |
| RA | FREM1 | GeneCards |
| RA | DDX41 | GeneCards |
| RA | NSD1 | GeneCards |
| RA | DNAJC21 | GeneCards |
| RA | SPRY4-IT1 | GeneCards |
| RA | YAP1 | GeneCards |
| RA | RAPGEF4 | GeneCards |
| RA | MAPK7 | GeneCards |
| RA | RABL2B | GeneCards |
| RA | MIR9-1 | GeneCards |
| RA | NF2 | GeneCards |
| RA | CARD8 | GeneCards |
| RA | ROS1 | GeneCards |
| RA | BTNL2 | GeneCards |
| RA | DHODH | GeneCards |
| RA | ATIC | GeneCards |
| RA | TNIP1 | GeneCards |
| RA | TWIST1 | GeneCards |
| RA | CCND3 | GeneCards |
| RA | FOLR2 | GeneCards |
| RA | ITGA2 | GeneCards |
| RA | ZEB1 | GeneCards |
| RA | TOP2A | GeneCards |
| RA | PRKG1 | GeneCards |
| RA | SH2D1A | GeneCards |
| RA | STEAP4 | GeneCards |
| RA | NLRP1 | GeneCards |
| RA | BST1 | GeneCards |
| RA | VWF | GeneCards |
| RA | LTB | GeneCards |
| RA | CLEC16A | GeneCards |
| RA | BTLA | GeneCards |
| RA | FYN | GeneCards |
| RA | PLG | GeneCards |
| RA | TIMP3 | GeneCards |
| RA | CASP7 | GeneCards |
| RA | ABI1 | GeneCards |
| RA | MT-CO1 | GeneCards |
| RA | SPRY2 | GeneCards |
| RA | HTRA1 | GeneCards |
| RA | TBC1D10C | GeneCards |
| RA | TRAF6 | GeneCards |
| RA | NPSR1 | GeneCards |
| RA | CD8A | GeneCards |
| RA | FBXL19-AS1 | GeneCards |
| RA | BMI1 | GeneCards |
| RA | ARF1 | GeneCards |
| RA | GEM | GeneCards |
| RA | ACTB | GeneCards |
| RA | SIGLEC1 | GeneCards |
| RA | TNFAIP6 | GeneCards |
| RA | CEBPB | GeneCards |
| RA | CCRL2 | GeneCards |
| RA | SIAE | GeneCards |
| RA | SNHG29 | GeneCards |
| RA | CYP17A1 | GeneCards |
| RA | REM1 | GeneCards |
| RA | HAS1 | GeneCards |
| RA | OLAH | GeneCards |
| RA | ZNF334 | GeneCards |
| RA | PHB | GeneCards |
| RA | CILP | GeneCards |
| RA | TKT | GeneCards |
| RA | SIPA1 | GeneCards |
| RA | RGL3 | GeneCards |
| RA | BCAR1 | GeneCards |
| RA | RRM1 | GeneCards |
| RA | CHGA | GeneCards |
| RA | SIX1 | GeneCards |
| RA | MIR23A | GeneCards |
| RA | SERPINB5 | GeneCards |
| RA | MVP | GeneCards |
| RA | CYP26C1 | GeneCards |
| RA | CEACAM3 | GeneCards |
| RA | CTAG1B | GeneCards |
| RA | CASC2 | GeneCards |
| RA | IFT22 | GeneCards |
| RA | MUC2 | GeneCards |
| RA | BSG | GeneCards |
| RA | MSLN | GeneCards |
| RA | FUT4 | GeneCards |
| RA | TGFBR1 | GeneCards |
| RA | IL22RA1 | GeneCards |
| RA | PRSS2 | GeneCards |
| RA | SUPT20H | GeneCards |
| RA | HNF1A-AS1 | GeneCards |
| RA | SNHG28 | GeneCards |
| RA | MIR424 | GeneCards |
| RA | LINC-ROR | GeneCards |
| RA | ELK3 | GeneCards |
| RA | EREG | GeneCards |
| RA | GPRC5A | GeneCards |
| RA | VHL | GeneCards |
| RA | PRKCI | GeneCards |
| RA | MIR193A | GeneCards |
| RA | EPS8 | GeneCards |
| RA | MIR212 | GeneCards |
| RA | CALR | GeneCards |
| RA | NAT1 | GeneCards |
| RA | MIR202 | GeneCards |
| RA | TNFRSF9 | GeneCards |
| RA | RUNX3 | GeneCards |
| RA | RGS14 | GeneCards |
| RA | SKP2 | GeneCards |
| RA | MIR301A | GeneCards |
| RA | DKK1 | GeneCards |
| RA | CALM1 | GeneCards |
| RA | ARHGAP26 | GeneCards |
| RA | PAX2 | GeneCards |
| RA | FGA | GeneCards |
| RA | E2F3 | GeneCards |
| RA | DYRK1B | GeneCards |
| RA | MMP14 | GeneCards |
| RA | RND1 | GeneCards |
| RA | F2RL3 | GeneCards |
| RA | MIR139 | GeneCards |
| RA | MIR144 | GeneCards |
| RA | PEBP1 | GeneCards |
| RA | MIR181C | GeneCards |
| RA | CD24 | GeneCards |
| RA | RINL | GeneCards |
| RA | S100A4 | GeneCards |
| RA | OGG1 | GeneCards |
| RA | SETD2 | GeneCards |
| RA | KRT14 | GeneCards |
| RA | CEACAM6 | GeneCards |
| RA | ETV5 | GeneCards |
| RA | MIR338 | GeneCards |

Table S5. Overlapping of the predicted targets of ingredients and RA.

| Ingredient -Target | Disease-Target | Venn |
| --- | --- | --- |
| GABRA3 GABRB2 GABRG2 | KRAS | HRAS |
| GABRA2 GABRB2 GABRG2 | HRAS | RAF1 |
| SLC6A1 | NRAS | BRAF |
| ACE | RAF1 | EGFR |
| REN | BRAF | PIK3CA |
| GABRR1 | SOS1 | RASA1 |
| EGLN1 | NF1 | TP53 |
| ANPEP | RRAS | MAP2K1 |
| GABBR2 GABBR1 | RASA2 | IL6 |
| F2 | EGFR | AKT1 |
| LTA4H | PIK3CA | SRC |
| OAT | RAP1A | RASGRP1 |
| ADORA3 | RASA1 | MAPK1 |
| ITGA2B ITGB3 | TP53 | PTPN22 |
| NR1H4 | MAP2K1 | KIT |
| GABBR1 | RALA | GRB2 |
| PLA2G2A | MRAS | RASSF1 |
| PLA2G5 | IL6 | FGFR2 |
| NOS1 | AKT1 | RET |
| NOS2 | RRAS2 | PTPN11 |
| NOS3 | RASGRF1 | FGFR1 |
| MME | SRC | ERBB2 |
| HSD17B3 | RASGRP1 | IL10 |
| DPP4 | SOS2 | CTNNB1 |
| XIAP | MAPK1 | RHOA |
| CTSK | RAC1 | CCND1 |
| KISS1R | RASGRF2 | NTRK1 |
| LAP3 | SHOC2 | NFKBIA |
| HSD11B1 | CDKN2A | MTOR |
| SLC1A2 | RASSF5 | PDGFRB |
| HLA-A | PTPN22 | EGF |
| ABAT | KIT | MAPK8 |
| SLC6A12 | GRB2 | RB1 |
| ACE2 | RASSF1 | JUN |
| PTGS2 | FGFR2 | CDK4 |
| CPN1 | RET | MAPK3 |
| SLC5A1 | PTPN11 | TERT |
| CAPN1 | FGFR1 | MYC |
| CTSB | ERBB2 | FOS |
| CHRNA4 CHRNB2 | PTEN | RASGRP3 |
| CHRNA3 CHRNB4 | IL10 | CASP8 |
| BBOX1 | RIT1 | TNF |
| EPHX2 | FASLG | PIK3CG |
| CNR1 | SHC1 | PLA2G2A |
| GABRA1 GABRB2 GABRG2 | RALB | VEGFA |
| SLC6A11 | MAP2K2 | CDKN1A |
| SLC6A13 | CTNNB1 | FLT3 |
| DNPEP | RHOA | JAK2 |
| PRKCA | RALGDS | BAX |
| CA2 | CCND1 | IGF1R |
| CA1 | PADI4 | FGF2 |
| CA12 | SYNGAP1 | PPARG |
| CA9 | NTRK1 | RARA |
| DPP7 | CDH1 | MET |
| SIRT2 | NFKBIA | ALK |
| FAP | MTOR | MAPK14 |
| NAAA | PDGFRB | IL2 |
| GFPT1 | EGF | ESR1 |
| SLC5A7 | RASA4 | EP300 |
| PGC | MAPK8 | CDK2 |
| DLG4 | RIN1 | PIK3CB |
| CTSH | LZTR1 | IL1B |
| GRM8 | RB1 | CXCL8 |
| CTSL | JUN | RAD51 |
| PLA2G4A | RAC2 | STAT3 |
| GRIK1 | MIR155 | MMP9 |
| GRIA1 | FGFR3 | AURKA |
| GRIK5 | CDK4 | CASP3 |
| GRIA4 | MIR146A | KDR |
| GRIK2 | RASA3 | PTGS2 |
| GRIK3 | RASGRP4 | MMP2 |
| BACE1 | MAPK3 | PIK3R1 |
| FKBP1A | TERT | BCL2L1 |
| SLC1A1 | RAP1B | CRP |
| GRM4 | RASAL1 | ESR2 |
| GRM5 | MIR150 | BCL2 |
| GRM7 | MYC | CHEK2 |
| PLA2G1B | APC | IKBKB |
| SLC18A3 | FOS | PLCG1 |
| METAP1 | RASGRP3 | FLT1 |
| SRD5A2 | SMAD4 | CSF1R |
| CNR2 | SLC22A4 | STAT1 |
| DDAH1 | CASP8 | IFNG |
| CTSD | RREB1 | TIMP1 |
| XPNPEP1 | TNF | HIF1A |
| XPNPEP2 | RASAL2 | PTK2 |
| PRKCE | PIK3CG | IRF1 |
| PABPC1 | RAPGEF2 | IL4 |
| NTSR1 | PLA2G2A | PRKCA |
| FPR2 | VEGFA | MMP1 |
| ENPEP | CDKN1A | MDM2 |
| GAA | RASGRP2 | CXCR4 |
| SI | FLT3 | FN1 |
| GSTK1 | ERAS | IDH1 |
| CFB | MIR21 | TIMP2 |
| GNPAT | JAK2 | CCNA2 |
| AR | MLH1 | NFE2L2 |
| LSS | MAP3K8 | RXRA |
| CHRNA7 | NFKBIL1 | MMP3 |
| GANC | BRCA1 | ABCB1 |
| GABRA1 | BAX | CAV1 |
| PPIA | RASAL3 | PIK3CD |
| BCHE | RAC3 | NFKB1 |
| ADH1B | ITGB2 | ODC1 |
| ADH1C | HLA-DRB1 | CTSB |
| LYZ | MIR145 | RELA |
| DAO | SPRED1 | SPP1 |
| GATM | CDC42 | JAK1 |
| P4HA1 | ATM | RCE1 |
| PRSS3 | MIR34A | CCL2 |
| ADH1A | KSR1 | CCR6 |
| HDAC3 | U2AF1 | MIF |
| CACNA2D1 | TGFB1 | CAT |
| RNPEP | MIR132 | CASP9 |
| KMO | MIR125A | RXRB |
| TH | BRCA2 | NOS2 |
| GRIA2 | TLR2 | INSR |
| GBA | IGF1R | FLT4 |
| PYGL | MIR31 | ICAM1 |
| SLC6A3 | RAP2B | IGFBP3 |
| KYNU | FGF2 | IGF2 |
| GRM1 | ARAF | CD274 |
| SLC22A6 | MIR17 | ITGAL |
| GRM2 | PPARG | BAD |
| CDC45 | MAPK10 | BIRC5 |
| PTPRC | RARA | MCL1 |
| CDC25A | DAB2IP | MPO |
| MAN1B1 | MET | AKT3 |
| GRM3 | CD44 | AKT2 |
| GRM6 | MIR126 | ERBB3 |
| FYN | RAP2A | GSTP1 |
| EGFR | ALK | CREB1 |
| LCK | MAPK14 | MMP13 |
| AGL | MIR143 | CD40 |
| MGAM | IL2 | SLC2A1 |
| GLA | ESR1 | CHUK |
| FABP4 | RERG | RPS6KB1 |
| PPARA | RAB5C | CTSL |
| CPT2 | RHEB | IL1A |
| FABP3 | EP300 | CDK6 |
| FABP5 | CDK2 | PLAU |
| PPARD | RAB5A | HSP90AA1 |
| FFAR1 | PIK3CB | PARP1 |
| FABP2 | RSU1 | TYMS |
| PEPD | RASSF2 | CDK1 |
| UGCG | RHOB | CYP1A1 |
| GANAB | RAN | LCK |
| KDM2A | CIITA | CFLAR |
| KDM6B | GATA2 | PLK1 |
| PHF8 | MIR127 | XIAP |
| KDM5C | IGF1 | ABL1 |
| SHBG | CD244 | CD80 |
| FUCA1 | IL1B | SYK |
| HMGCR | CXCL8 | VCAM1 |
| GBA2 | RAD51 | HSPB1 |
| KDM4C | RASL11A | TLR9 |
| GLB1 | STAT3 | HSPA5 |
| VDR | RIT2 | NR3C1 |
| GPBAR1 | MMP9 | MMP7 |
| AKR1B10 | CBL | E2F1 |
| POLB | AURKA | ABCC1 |
| CPB2 | RAB5B | ALOX5 |
| PLG | MIR221 | EPHA2 |
| ODC1 | MIR27A | TP63 |
| CPA1 | MIR222 | PRKCB |
| KDM4E | RARB | SELE |
| PTGS1 | RHOD | ELK1 |
| AKR1B1 | STK11 | MAPK9 |
| GOT2 | MIR15A | CD40LG |
| ME2 | MIR141 | PDPK1 |
| GOT1 | CASP3 | CXCR3 |
| OTC | NKIRAS2 | LGALS3 |
| CTH | KDR | ELANE |
| MAOB | NKIRAS1 | CD86 |
| ADSS | PTGS2 | IL23A |
| KDM3A | RHOC | CYP1B1 |
| EGLN2 | MIRLET7A1 | TTR |
| CHRM4 | MIR205 | TYMP |
| CHRM5 | MIR182 | CXCL10 |
| CHRM2 | RIN2 | GSTM1 |
| CHRM1 | KSR2 | JAK3 |
| CHRM3 | RASSF6 | DNMT3B |
| TPI1 | RAB8A | CCNB1 |
| CDC25B | CSF1 | CXCL2 |
| ESR1 | MSH2 | DAPK1 |
| NCOA2 | ANXA5 | SOD2 |
| CXCL8 | MIR200A | CFTR |
| RXRA | MMP2 | AXL |
| CAT | FAS | FGF1 |
| HAS2 | RASD1 | CYP3A4 |
| DRD2 | HGF | TOP1 |
| DRD3 | RGL2 | NQO1 |
| DRD1 | RAB25 | GADD45A |
| HTR1A | RAB27A | PAK1 |
| HTR6 | PIK3R1 | CCR1 |
| HTR7 | MIR483 | HDAC1 |
| PTPRCAP | RAB11A | ITGA2B |
| HTR2B | BCL2L1 | CX3CR1 |
| ADRA1D | CRP | PCNA |
| HTR2A | MIR34C | CTSK |
| DRD4 | CSF2 | PTGS1 |
| HTR5A | ASXL1 | AURKB |
| ADRA1A | CNKSR1 | CTSG |
| HTR1D | MIR34B | HSF1 |
| ADRB1 | MIR10A | CDK5 |
| OPRM1 | MIR20A | SLC29A1 |
| DRD5 | RAP2C | PTPRC |
| HTR2C | MIR214 | PPP1CC |
| KCNH2 | MIR200C | MMP12 |
| ADRA1B | ESR2 | CCR7 |
| TSPO | BCL2 | ADAM17 |
| SLC6A4 | PLCE1 | SERPINE1 |
| ADRA2A | MIR96 | ABCG2 |
| ADRA2C | MIR128-2 | MMP8 |
| ADRA2B | MIR183 | PRKCG |
| HTR1B | CHEK2 | CCKBR |
| HRH2 | MIR203A | PRKACA |
| PPARG | MIR210 | SIRT1 |
| ADRB2 | CXCL12 | RAPGEF4 |
| CDK5R1 CDK5 | IKBKB | DHODH |
| GSK3B | PLCG1 | TOP2A |
| CDK2 CCNA1 CCNA2 | FLT1 | FYN |
| NR3C2 | RAB3A | PLG |
| HPGD | MIR148A | CASP7 |
| CYP19A1 | CSF1R | CYP17A1 |
| MGLL | NGF | RRM1 |
| TRPC6 | MUC1 | PRKCI |
| TRPC3 | IL2RA | MMP14 |
| JAK3 | RAPH1 |  |
| PTPN1 | STAT1 |  |
| SIGMAR1 | IDH2 |  |
| PIM1 | MIR100 |  |
| DYRK1A | IFNG |  |
| STAT3 | RAPGEF5 |  |
| CLK1 | MIR25 |  |
| DYRK2 | RAB11B |  |
| PRKCG | MIR32 |  |
| VCAM1 | MIRLET7B |  |
| GABRA5 | MAP2K4 |  |
| MAP2K1 | TIMP1 |  |
| ADAMTS5 | GNAS |  |
| RPS6KB1 | RAB7A |  |
| KAT2B | HIF1A |  |
| FLT3 | RAB10 |  |
| CLK3 | PTK2 |  |
| PTGES | CDKN1B |  |
| CSF1R | IRF1 |  |
| MAPKAPK2 | RASIP1 |  |
| KDR | RAB13 |  |
| CDK1 | IQGAP1 |  |
| IRAK4 | PRKD1 |  |
| ALOX15 | ANGPT1 |  |
| ALOX12 | MIR106B |  |
| PIK3CB | MIR335 |  |
| PIK3CG | TGFA |  |
| PIK3CA | NOTCH1 |  |
| PDGFRB | RAB9B |  |
| FLT4 | IL2RB |  |
| SRC | TET2 |  |
| ICAM1 | IL4 |  |
| SELE | RAB35 |  |
| PPP5C | CNKSR2 |  |
| CCNB3 CDK1 CCNB1 CCNB2 | PRKCA |  |
| DBF4 CDC7 | MMP1 |  |
| AURKA | RIN3 |  |
| TRPV1 | RAB23 |  |
| TRPM8 | STAT4 |  |
| PGR | MDM2 |  |
| MMP3 | RABL3 |  |
| MMP2 | TEK |  |
| ADORA2A | RAB31 |  |
| MAPK1 | RAPGEF6 |  |
| PDE10A | MIR18A |  |
| CXCR3 | RAB36 |  |
| CDK2 | ERCC6 |  |
| MMP9 | CXCR4 |  |
| PDGFRA PDGFRB | FN1 |  |
| AURKB | MIRLET7D |  |
| ROCK2 | IDH1 |  |
| FGFR1 | TIMP2 |  |
| AKT1 | IL17A |  |
| PLAU | MIR140 |  |
| TNFSF15 | CCNA2 |  |
| JUN | CRABP2 |  |
| IL6 | RRAD |  |
| CASP3 | IQGAP3 |  |
| POR | KMT2A |  |
| COMT | RAB3B |  |
| CREB1 | RAB2A |  |
| CCL2 | RAB33A |  |
| DUOX2 | MIR93 |  |
| IL2 | MIR181A2 |  |
| PLAT | MIR107 |  |
| IL1A | DNMT1 |  |
| GCLC | NFE2L2 |  |
| GSS | RAB14 |  |
| ABCG2 | RXRA |  |
| CRTC2 | MMP3 |  |
| NOX4 | ABCB1 |  |
| CA7 | HDAC9 |  |
| CA4 | MIR10B |  |
| ACHE | MIR19A |  |
| NQO2 | GDNF |  |
| RPS6KA3 | CAV1 |  |
| NMUR2 | RAB4A |  |
| CD38 | MIR199B |  |
| PDE5A | CD4 |  |
| TNF | PDGFRA |  |
| ADORA1 | MALAT1 |  |
| XDH | PIK3CD |  |
| ALOX5 | RASSF3 |  |
| SLC29A1 | MIR451A |  |
| TERT | SETBP1 |  |
| KCNA3 | GREM1 |  |
| APP | ITGAM |  |
| CYP1B1 | RASL12 |  |
| MCL1 | MIR200B |  |
| ALDH2 | NPM1 |  |
| ITGAV ITGB3 | RASL11B |  |
| ITGAV ITGB6 | ITGA8 |  |
| F10 | RUNX1 |  |
| CA13 | RASL10A |  |
| ITGB1 ITGA5 | RAB1A |  |
| HSP90AB1 | RAB6A |  |
| CHEK2 | TMCO1 |  |
| CHEK1 | NFKB1 |  |
| PRKCD | RASGEF1A |  |
| PRKCB | MIR204 |  |
| PRKCH | RAPGEF1 |  |
| DNM2 | RAB30 |  |
| MAPT | ITGB1 |  |
| GPR35 | MIR378A |  |
| AVPR2 | TGFB2 |  |
| TOP2A | DDR2 |  |
| MAOA | AREG |  |
| IGF1R | MIR499A |  |
| INSR | MIR30E |  |
| GLO1 | KITLG |  |
| MYLK | DNMT3A |  |
| MPO | CTLA4 |  |
| PIK3R1 | ODC1 |  |
| DAPK1 | RAB28 |  |
| SYK | G3BP1 |  |
| PTK2 | RXRG |  |
| HSD17B2 | CYP26A1 |  |
| MMP13 | H19 |  |
| CA3 | CTSB |  |
| PLK1 | PPP2R1B |  |
| CA6 | RELA |  |
| PKN1 | RHOH |  |
| CA14 | ALB |  |
| CSNK2A1 | MIR29A |  |
| MET | DIRAS1 |  |
| NEK2 | RAB4B |  |
| CXCR1 | RAB18 |  |
| CAMK2B | SPP1 |  |
| ALK | RASEF |  |
| NEK6 | JAK1 |  |
| CA5A | INS |  |
| AXL | TNFRSF1B |  |
| PRSS1 | CD79A |  |
| ABCB1 | RASA4B |  |
| ABCC1 | HOTAIR |  |
| PDK1 | RCE1 |  |
| HSP90AA1 | ALDH1A2 |  |
| BCL2L1 | CCL2 |  |
| BCL2 | MIRLET7C |  |
| MELK | TIAM1 |  |
| HSP90B1 | MEN1 |  |
| CTNNB1 | RASSF7 |  |
| OPRD1 | CCR6 |  |
| PARP1 | SPRY4 |  |
| TTR | MSH3 |  |
| TNKS2 | RASSF8 |  |
| TNKS | PTCH1 |  |
| TOP1 | ALDH1A1 |  |
| ARG1 | TNFRSF1A |  |
| AGTR1 | MIR372 |  |
| CNOT7 | MIRLET7E |  |
| ESR2 | MIR342 |  |
| ERBB2 | MIR29C |  |
| GCGR | MIR324 |  |
| PRKCI | RAB34 |  |
| CHUK | MIF |  |
| FASN | CAT |  |
| RELA | RHOV |  |
| CDK6 | CASP9 |  |
| OPRK1 | RAB29 |  |
| HSD17B1 | RXRB |  |
| KLK1 | RRAGB |  |
| KLK2 | NOS2 |  |
| CCR4 | RASSF4 |  |
| FBP1 | NTRK2 |  |
| DHODH | CSF3 |  |
| DHFR | IL1RN |  |
| CALCA | UCA1 |  |
| GPR84 | INSR |  |
| ACP1 | NBN |  |
| KLKB1 | RAB3C |  |
| CFTR | RALBP1 |  |
| RAF1 | DIRAS2 |  |
| TBXAS1 | FLT4 |  |
| ESRRA | MAP2K7 |  |
| ESRRB | AXIN2 |  |
| TYR | RARS1 |  |
| MIF | ICAM1 |  |
| PFKFB3 | IGFBP3 |  |
| CBR1 | IGF2 |  |
| PTPRS | RHOG |  |
| SLC6A2 | CD274 |  |
| PON1 | RAB24 |  |
| STS | PDCD1 |  |
| TLR9 | BMP4 |  |
| CA5B | ITGAL |  |
| SNCA | RHOJ |  |
| IGFBP3 | BAD |  |
| CRHR1 | RAB1B |  |
| BAD | BIRC5 |  |
| PTP4A3 | MCL1 |  |
| CDC7 | MPO |  |
| FNTA FNTB | MIR26A1 |  |
| IGFBP6 | RASGEF1B |  |
| IGFBP4 | ARHGEF2 |  |
| IGFBP5 | BDNF |  |
| IGFBP2 | SERPINA3 |  |
| IGFBP1 | AKT3 |  |
| NTRK1 | AKT2 |  |
| PIM2 | DIRAS3 |  |
| BRAF | ERBB3 |  |
| PRMT1 | MKI67 |  |
| CXCR2 | FNTB |  |
| CES2 | IL3 |  |
| MAPK14 | GSTP1 |  |
| VEGFA | ENO1 |  |
| FOS | CREB1 |  |
| CDKN1A | MIR30D |  |
| EIF6 | MIR486-1 |  |
| BAX | MSH6 |  |
| TP63 | RAB22A |  |
| LDLR | FGF10 |  |
| STAT1 | MMP13 |  |
| CYP3A4 | VEGFC |  |
| CAV1 | CD40 |  |
| MTTP | GAPDH |  |
| APOB | RAB27B |  |
| SOD2 | RAB40C |  |
| ECE1 | REL |  |
| IL4 | SLC2A1 |  |
| CPT1A | RAB33B |  |
| AHR | CHUK |  |
| RHOA | EGR1 |  |
| MT2A | PTHLH |  |
| FCER2 | RASL10B |  |
| EP300 | MIR133B |  |
| ATP5F1B | CYP2A6 |  |
| ND6 | RPS6KB1 |  |
| HSD3B2 | CTSL |  |
| HSD3B1 | IL1A |  |
| TFF1 | FCRL3 |  |
| TRPM2 | FGFR4 |  |
| GH1 | TNFRSF10B |  |
| PPARGC1B | RASSF9 |  |
| B4GALT4 | RAB9A |  |
| GHR | EZH2 |  |
| TGFB1I1 | CDK6 |  |
| CD5L | HLA-DQB1 |  |
| P4HB | SMO |  |
| CYP21A2 | A2ML1 |  |
| GADD45A | NCAM1 |  |
| BARD1 | RAB3D |  |
| RBM45 | THBS1 |  |
| BAP1 | PLAU |  |
| RAD51 | HSP90AA1 |  |
| MAPK8 | MIR130A |  |
| IFNG | FCGR3A |  |
| CD80 | TNFSF11 |  |
| CD86 | IFNA1 |  |
| CXCR4 | RAB39A |  |
| BIRC3 | ACP5 |  |
| CD274 | IL7 |  |
| IL23A | ETV6 |  |
| CCR7 | TFRC |  |
| CD1A | TNFSF10 |  |
| CD40 | PARP1 |  |
| CD14 | IFNB1 |  |
| C3 | HLA-DPB1 |  |
| VTCN1 | RAB32 |  |
| NUAK1 | AFDN |  |
| AKR1C2 | MIR373 |  |
| AKR1C1 | FHIT |  |
| AKR1C3 | SELL |  |
| AKR1C4 | GNAQ |  |
| AKR1A1 | TYMS |  |
| APEX1 | MIR192 |  |
| MPG | IL6ST |  |
| SLC22A12 | PRTN3 |  |
| MMP12 | IL15 |  |
| SCN5A | SP1 |  |
| F7 | TNFRSF10A |  |
| CCND1 | RAB2B |  |
| CASP9 | RAB38 |  |
| IL10 | MIRLET7G |  |
| EGF | CDK1 |  |
| RB1 | CYP1A1 |  |
| AHSA1 | LCK |  |
| ELK1 | TLR4 |  |
| NFKBIA | XRCC3 |  |
| CASP8 | BMP6 |  |
| SOD1 | SRSF2 |  |
| MMP1 | MIRLET7A3 |  |
| HIF1A | DCK |  |
| RUNX1T1 | NME1 |  |
| ACACA | SPRY1 |  |
| HMOX1 | LOX |  |
| CYP1A2 | TRAF1 |  |
| MYC | MTHFR |  |
| F3 | HLA-DMA |  |
| GJA1 | CYCS |  |
| CYP1A1 | MIR146B |  |
| IL1B | RRAGC |  |
| PTGER3 | PROM1 |  |
| BIRC5 | SERPINH1 |  |
| HSPB1 | APBB1IP |  |
| SULT1E1 | SRF |  |
| NR1I2 | RAB21 |  |
| CCNB1 | CFLAR |  |
| THBD | SPRED2 |  |
| SERPINE1 | PLK1 |  |
| COL1A1 | MIR185 |  |
| NCF1 | RAB26 |  |
| GSTP1 | XIAP |  |
| NFE2L2 | NUP214 |  |
| NQO1 | FNTA |  |
| PSMD3 | GFRA1 |  |
| SLC2A4 | CD28 |  |
| COL3A1 | CD69 |  |
| CXCL11 | ABL1 |  |
| CXCL2 | HMGB1 |  |
| DCAF5 | CCR5 |  |
| NR1I3 | HLA-DQA1 |  |
| CLDN4 | TGFBR2 |  |
| HSF1 | LRRK2 |  |
| CRP | PML |  |
| CXCL10 | IL6R |  |
| SPP1 | CYP26B1 |  |
| RUNX2 | IL11 |  |
| RASSF1 | COL2A1 |  |
| E2F1 | MIR98 |  |
| E2F2 | CCL3 |  |
| ACPP | CD80 |  |
| IGF2 | EPOR |  |
| CD40LG | OSM |  |
| IRF1 | CCL5 |  |
| ERBB3 | RAB8B |  |
| DIO1 | TRAF3 |  |
| PCOLCE | MIR193B |  |
| NPEPPS | RAB37 |  |
| HK2 | TNFRSF11B |  |
| RASA1 | RHEBL1 |  |
| GSTM1 | TGFB3 |  |
| GSTM2 | RRAGA |  |
| AMY1A | RAB6B |  |
| GRK6 | MGMT |  |
| MDM2 | SYK |  |
| PCNA | MIR137 |  |
| CASP7 | ACAN |  |
| NUF2 | PIK3R3 |  |
| ADCY2 | EPO |  |
| FGF1 | HSPD1 |  |
| FGF2 | IL18 |  |
| HPSE | PLA2G10 |  |
| AMY2A | FGF8 |  |
| TYMS | VCAM1 |  |
| FPGS | CTNND1 |  |
| TACR2 | HSPB1 |  |
| NPC1L1 | IRF5 |  |
| PTPRA | RAB39B |  |
| CASP6 | BCL10 |  |
| CASP1 | MIR30B |  |
| FOLH1 | BUB1 |  |
| PTPN2 | RAB42 |  |
| PTPN22 | RAB43 |  |
| ATP1A1 | GNB1 |  |
| EDNRA | GRP |  |
| LGALS3 | TLR9 |  |
| CCKBR | SLC22A18 |  |
| ALOX5AP | LIF |  |
| GPR34 | HSPA5 |  |
| SPTLC1 | CXCL1 |  |
| SPTLC2 | NR3C1 |  |
| P2RY12 | MMP7 |  |
| SIRT1 | E2F1 |  |
| ITGA2 ITGB3 | MBL2 |  |
| FDFT1 | HLA-DMB |  |
| ITGB5 ITGAV | IL32 |  |
| TMPRSS11D | PALB2 |  |
| ST14 | FCGR2A |  |
| P2RY10 | SNAI1 |  |
| RRM1 | IL1R1 |  |
| LGALS4 | ABCC1 |  |
| LGALS8 | HLA-DRA |  |
| TNNC1 TNNT2 TNNI3 | RRAGD |  |
| CD22 | RHOQ |  |
| LGALS7 | MIR429 |  |
| LGALS9 | MIR502 |  |
| TMPRSS15 | CDH2 |  |
| PDYN | HLA-DQA2 |  |
| GRB2 | ALOX5 |  |
| MTOR | FOXP3 |  |
| RORC | EPHA2 |  |
| LIPC | TP63 |  |
| IMPDH1 | RGL1 |  |
| IMPDH2 | PXN |  |
| HDAC6 | KRT7 |  |
| HDAC8 | RAB17 |  |
| HDAC1 | RAB40B |  |
| AKT2 | NRG1 |  |
| RPS6KA1 | IL13 |  |
| ROCK1 | CXCR5 |  |
| LTB4R | PRKCB |  |
| ITGAV ITGB1 | SELE |  |
| MMP8 | VAV1 |  |
| S1PR1 | IL1RAPL2 |  |
| S1PR3 | ZFAS1 |  |
| F3 F7 | S100A9 |  |
| ITGAL | PLAUR |  |
| SRD5A1 | CTNNA1 |  |
| ITGA2B | RAB12 |  |
| F11 | MICB |  |
| FANCF | HLA-DOA |  |
| SLC28A2 | HLA-DRB5 |  |
| F9 | MEG3 |  |
| GPR174 | ELK1 |  |
| CALCRL | ANXA1 |  |
| LNPEP | SAA1 |  |
| ERAP2 | MAPK9 |  |
| ERAP1 | CD40LG |  |
| CSNK2A2 | PDPK1 |  |
| TYMP | CXCR3 |  |
| MAPK9 | BGLAP |  |
| C3AR1 | MST1R |  |
| BCL2A1 | CEACAM5 |  |
| HCAR2 | PRKN |  |
| DNMT3B | FCGR2B |  |
| PRUNE1 | SF3B1 |  |
| METAP2 | MIR199A1 |  |
| ABL1 | LGALS3 |  |
| KIT | RASD2 |  |
| PPP2CA | BCL2L11 |  |
| PRKACA | MIR30A |  |
| AKT3 | ELANE |  |
| ITGB1 ITGA4 | CDKN2B |  |
| DOT1L | GRAP |  |
| CDK4 | CEBPA |  |
| MANBA | CD86 |  |
| IKBKB | FCGR3B |  |
| PPP3CA | H2AC18 |  |
| SLPI | IL23A |  |
| CCNA2 | RABL6 |  |
| PYGM | MACIR |  |
| NCOA1 | RAB41 |  |
| OLR1 | RERGL |  |
| IDH1 | WRN |  |
| DUSP3 | ACVR1B |  |
| CCR1 | CYP1B1 |  |
| PRKCZ | CR1 |  |
| SQLE | TTR |  |
| VCP | RAB20 |  |
| LDHA | CDX2 |  |
| LDHB | KRT20 |  |
| CDK1 CCNB1 | IRS1 |  |
| PSMG3 | RADIL |  |
| WEE1 | TYMP |  |
| TP53 | ERBB4 |  |
| ERN1 | SLC19A1 |  |
| TRAP1 | WT1 |  |
| TDP1 | IL22 |  |
| FKBP5 | CXCL10 |  |
| ADCYAP1R1 | CDKN2B-AS1 |  |
| GRK7 | KRT19 |  |
| GRK3 | GSTM1 |  |
| GRK2 | JAK3 |  |
| GRK1 | DNMT3B |  |
| GRK5 | KNDC1 |  |
| GRK4 | TSC1 |  |
| UGT2B7 | LTA |  |
| C5AR1 | TUG1 |  |
| CYP17A1 | PTH |  |
| SERPINA6 | CCNB1 |  |
| CTRC | ILK |  |
| LIPA | RAP1GAP |  |
| PSEN2 PSENEN NCSTN APH1A PSEN1 APH1B | CXCL2 |  |
| NR3C1 | CD82 |  |
| TRPV4 | RHOT1 |  |
| BRD4 | STAT5A |  |
| IDO1 | RHOT2 |  |
| PRCP | GDF15 |  |
| SLC6A9 | LIG4 |  |
| PREP | IL18R1 |  |
| CTSS | EIF4EBP1 |  |
| RASGRP1 | STAT5B |  |
| PPM1B | RAB15 |  |
| PPP1CC | RAB19 |  |
| MAPK3 | RAB44 |  |
| PPP1CA | PALLD |  |
| P2RX7 | DAPK1 |  |
| RAPGEF4 | SOD2 |  |
| SCN9A | MAP2K3 |  |
| PRKCQ | CFTR |  |
| MTNR1A | COMP |  |
| MTNR1B | AXL |  |
| KCNQ2 | MIR181A1 |  |
| PAM | MUC5AC |  |
| KCNQ1 | DMTF1 |  |
| OPRL1 | SOX2 |  |
| JAK2 | GPI |  |
| SLC10A2 | FGF1 |  |
| PKM | MYB |  |
| PIK3CD | RAB7B |  |
| PRMT3 | CYP3A4 |  |
| HSD17B7 | KMT2D |  |
| SLC5A2 | MXRA5 |  |
| ITGA3 | MIR320A |  |
| ABCB11 | MIR197 |  |
| KDM1A | CCAT1 |  |
| NFKB1 | RASSF10 |  |
| SOAT1 | TOP1 |  |
| SOAT2 | BMP2 |  |
| GABRB3 GABRA3 GABRG2 | NQO1 |  |
| GABRB3 GABRG2 GABRA1 | GADD45A |  |
| NAE1 | TSC2 |  |
| PTAFR | HMMR |  |
| RXRB | PAK1 |  |
| RARA | SMAD2 |  |
| PIM3 | JAM3 |  |
| CCND1 CDK4 | NR4A2 |  |
| CCNE2 CDK2 CCNE1 | RTEL1 |  |
| ADAM17 | GAS5 |  |
| ADORA2B | CCL4 |  |
| TACR1 | HP |  |
| LIPG | GGT1 |  |
| MMP7 | TNFSF13B |  |
| ELANE | NAT2 |  |
| AVPR1A | CCR1 |  |
| AVPR1B | RHOF |  |
| OXTR | HDAC1 |  |
| EIF4A1 | ITGA2B |  |
| CTSE | EPHB2 |  |
| PGA5 | PIK3R2 |  |
| CCNA2 CDK2 | MYCN |  |
| HRAS | KRT8 |  |
| BACE2 | CX3CR1 |  |
| CFD | YWHAE |  |
| BTK | CCND2 |  |
| TMIGD3 | RAB6C |  |
| ADAM10 | PCNA |  |
| PSMB8 | NEAT1 |  |
| GHRHR | IL26 |  |
| PSMB5 | BAK1 |  |
| EDNRB | APOH |  |
| CCKAR | CDKN3 |  |
| TUBB3 | IL24 |  |
| AURKC | CTSK |  |
| MERTK | TNFRSF11A |  |
| PSMB2 | IL10RA |  |
| MAP3K11 | CX3CL1 |  |
| MAP3K9 | MIR142 |  |
| MAP3K10 | PDGFB |  |
| CETP | ADAMTS4 |  |
| CDK5 | RELB |  |
| BDKRB1 | CXCL6 |  |
| SLC2A1 | PTGS1 |  |
| CX3CR1 | AURKB |  |
| RET | CTSG |  |
| CDK7 | PMS2 |  |
| CDK9 | HSF1 |  |
| PSEN1 | NGFR |  |
| GCK | NKX2-1 |  |
| CCNE1 CDK2 | SYVN1 |  |
| CDK7 CCNH | CDK5 |  |
| CDK9 CCNT1 | PTK2B |  |
| PAK3 | BCR |  |
| PAK2 | CYP2E1 |  |
| PAK1 | SLC29A1 |  |
| PSMB9 | PVT1 |  |
| MMP14 | MECOM |  |
| HSPA5 | CD247 |  |
| HRH1 | PLB1 |  |
| CCR3 | EZR |  |
| ADCY1 | MAP3K1 |  |
| P2RX3 | PTPRC |  |
| RASGRP3 | RAB40A |  |
| PIK3CA PIK3R1 | MPL |  |
| PDPK1 | LECT2 |  |
| HK1 | ICMT |  |
| CCR6 | GNRH1 |  |
| FAAH | RABL2A |  |
| F2R | HBEGF |  |
| FGFR2 | S100A12 |  |
| SAE1 UBA2 | PPP1CC |  |
| EPHB4 | IL16 |  |
| KLK3 | FLG |  |
| PDE4B | PAX8 |  |
| SCARB1 | ITGA5 |  |
| KDM5A | TNFRSF13C |  |
| NEK1 | MMP12 |  |
| PLA2G7 | CCR7 |  |
| KIF11 | KLF4 |  |
| NISCH | ADAM17 |  |
| DAPK3 | CHI3L1 |  |
| DAPK2 | XRCC1 |  |
| STK17B | CRABP1 |  |
| STK17A | STK4 |  |
| FLT1 | PRRC2A |  |
| EEF2K | IL19 |  |
| PTK6 | HLA-B |  |
| ACVR1 | SMAD3 |  |
| MAP4K5 | CCN2 |  |
| MAP4K3 | MIR196A1 |  |
| CASR | SERPINE1 |  |
| HCRTR2 | CCR2 |  |
| JAK1 | CCL20 |  |
| DCTPP1 | SLC11A1 |  |
| CYP24A1 | ABCG2 |  |
| LIMK1 | FOXO3 |  |
| TAAR1 | IL23R |  |
| LIMK2 | ETS1 |  |
| HDAC7 | RETN |  |
| CTSG | CAST |  |
| PAK4 | RARG |  |
| TRPA1 | RHOU |  |
| KLK7 | IFI27 |  |
| NCOR2 HDAC3 | AGTR2 |  |
| CAPN2 | FSTL1 |  |
| SEM1 PSMD8 PSMD4 PSMD13 PSMD7 PSMD6 PSMD11 PSMD12 PSMD3 PSMD2 PSMC5 PSMC3 PSMC6 PSMC4 PSMC1 PSMC2 ADRM1 PSMD14 PSMD1 PSMB7 PSMB4 PSMB3 PSMB11 PSMB10 PSMA8 PSMA7 PSMA6 PSMA5 PSMA4 PSMA3 PSMA2 PSMA1 PSMB6 PSMB9 PSMB8 PSMB5 PSMB1 PSMB2 | PPP1CB |  |
| KLK5 | PTPN3 |  |
| ZAP70 | SAA4 |  |
| CMA1 | FBXW7 |  |
| LIPE | XIST |  |
| TYK2 | TNFSF13 |  |
| PTPN11 | NRP1 |  |
| PTPRF | IQGAP2 |  |
| PDE4D | IL37 |  |
| MPEG1 | ANGPT2 |  |
| PLCG1 | TP73 |  |
| PTGDR | CREBBP |  |
| TBXA2R | MUC4 |  |
| SLC10A1 | MMP8 |  |
| UBLCP1 | CXCL5 |  |
| CD81 | MAFB |  |
| PTGDR2 | ARHGAP5 |  |
| EPHA2 | MIR195 |  |
| THRA | VEGFD |  |
| THRB | PRKCG |  |
| PTGFR | CCKBR |  |
| BMP1 | DDX39B |  |
| CYSLTR1 | EPCAM |  |
| ATP12A | BRAP |  |
| ST3GAL1 | B2M |  |
| NR1H2 | KRT18 |  |
| S1PR2 | REM2 |  |
| ITGA4 | TERC |  |
| RCE1 | IL17RA |  |
| PGGT1B FNTA | PRKACA |  |
| TUBB1 | SIRT1 |  |
| CTSA | RBP4 |  |
| BIRC2 | ITGA6 |  |
| CFLAR | CXCL9 |  |
| CDC37 | ARF6 |  |
| TIMP1 | IL18BP |  |
| TIMP2 | TAGAP |  |
| FN1 | MIR223 |  |
| COL7A1 | FREM1 |  |
| COL4A4 | DDX41 |  |
|  | NSD1 |  |
|  | DNAJC21 |  |
|  | SPRY4-IT1 |  |
|  | YAP1 |  |
|  | RAPGEF4 |  |
|  | MAPK7 |  |
|  | RABL2B |  |
|  | MIR9-1 |  |
|  | NF2 |  |
|  | CARD8 |  |
|  | ROS1 |  |
|  | BTNL2 |  |
|  | DHODH |  |
|  | ATIC |  |
|  | TNIP1 |  |
|  | TWIST1 |  |
|  | CCND3 |  |
|  | FOLR2 |  |
|  | ITGA2 |  |
|  | ZEB1 |  |
|  | TOP2A |  |
|  | PRKG1 |  |
|  | SH2D1A |  |
|  | STEAP4 |  |
|  | NLRP1 |  |
|  | BST1 |  |
|  | VWF |  |
|  | LTB |  |
|  | CLEC16A |  |
|  | BTLA |  |
|  | FYN |  |
|  | PLG |  |
|  | TIMP3 |  |
|  | CASP7 |  |
|  | ABI1 |  |
|  | MT-CO1 |  |
|  | SPRY2 |  |
|  | HTRA1 |  |
|  | TBC1D10C |  |
|  | TRAF6 |  |
|  | NPSR1 |  |
|  | CD8A |  |
|  | FBXL19-AS1 |  |
|  | BMI1 |  |
|  | ARF1 |  |
|  | GEM |  |
|  | ACTB |  |
|  | SIGLEC1 |  |
|  | TNFAIP6 |  |
|  | CEBPB |  |
|  | CCRL2 |  |
|  | SIAE |  |
|  | SNHG29 |  |
|  | CYP17A1 |  |
|  | REM1 |  |
|  | HAS1 |  |
|  | OLAH |  |
|  | ZNF334 |  |
|  | PHB |  |
|  | CILP |  |
|  | TKT |  |
|  | SIPA1 |  |
|  | RGL3 |  |
|  | BCAR1 |  |
|  | RRM1 |  |
|  | CHGA |  |
|  | SIX1 |  |
|  | MIR23A |  |
|  | SERPINB5 |  |
|  | MVP |  |
|  | CYP26C1 |  |
|  | CEACAM3 |  |
|  | CTAG1B |  |
|  | CASC2 |  |
|  | IFT22 |  |
|  | MUC2 |  |
|  | BSG |  |
|  | MSLN |  |
|  | FUT4 |  |
|  | TGFBR1 |  |
|  | IL22RA1 |  |
|  | PRSS2 |  |
|  | SUPT20H |  |
|  | HNF1A-AS1 |  |
|  | SNHG28 |  |
|  | MIR424 |  |
|  | LINC-ROR |  |
|  | ELK3 |  |
|  | EREG |  |
|  | GPRC5A |  |
|  | VHL |  |
|  | PRKCI |  |
|  | MIR193A |  |
|  | EPS8 |  |
|  | MIR212 |  |
|  | CALR |  |
|  | NAT1 |  |
|  | MIR202 |  |
|  | TNFRSF9 |  |
|  | RUNX3 |  |
|  | RGS14 |  |
|  | SKP2 |  |
|  | MIR301A |  |
|  | DKK1 |  |
|  | CALM1 |  |
|  | ARHGAP26 |  |
|  | PAX2 |  |
|  | FGA |  |
|  | E2F3 |  |
|  | DYRK1B |  |
|  | MMP14 |  |
|  | RND1 |  |
|  | F2RL3 |  |
|  | MIR139 |  |
|  | MIR144 |  |
|  | PEBP1 |  |
|  | MIR181C |  |
|  | CD24 |  |
|  | RINL |  |
|  | S100A4 |  |
|  | OGG1 |  |
|  | SETD2 |  |
|  | KRT14 |  |
|  | CEACAM6 |  |
|  | ETV5 |  |
|  | MIR338 |  |

Table S6. The key targets and their topological parameters by PPI.

| Gene symbol | Degree | Closeness | Betweenness |
| --- | --- | --- | --- |
| TP53 | 183 | 0.8376 | 0.0377 |
| AKT1 | 178 | 0.8225 | 0.0322 |
| VEGFA | 170 | 0.7993 | 0.0230 |
| MAPK3 | 162 | 0.7774 | 0.0261 |
| MYC | 157 | 0.7643 | 0.0211 |
| CASP3 | 157 | 0.7643 | 0.0180 |
| STAT3 | 156 | 0.7617 | 0.0160 |
| IL6 | 155 | 0.7592 | 0.0210 |
| EGFR | 154 | 0.7567 | 0.0207 |
| MAPK1 | 151 | 0.7467 | 0.0228 |
| SRC | 150 | 0.7467 | 0.0224 |
| TNF | 149 | 0.7443 | 0.0180 |
| HRAS | 148 | 0.7418 | 0.0281 |
| JUN | 147 | 0.7394 | 0.0134 |
| EGF | 147 | 0.7394 | 0.0126 |
| MAPK8 | 142 | 0.7276 | 0.0127 |
| CCND1 | 139 | 0.7206 | 0.0112 |
| FN1 | 131 | 0.7028 | 0.0134 |
| CXCL8 | 129 | 0.6985 | 0.0104 |
| ESR1 | 127 | 0.6942 | 0.0126 |
| MMP9 | 127 | 0.6942 | 0.0093 |
| MTOR | 124 | 0.6879 | 0.0084 |
| HSP90AA1 | 123 | 0.6858 | 0.0124 |
| ERBB2 | 122 | 0.6837 | 0.0084 |
| PTGS2 | 120 | 0.6796 | 0.0109 |
| BCL2L1 | 116 | 0.6716 | 0.0066 |
| CTNNB1 | 115 | 0.6696 | 0.0099 |
| IL10 | 114 | 0.6676 | 0.0060 |
| IL1B | 109 | 0.6542 | 0.0063 |
| FOS | 109 | 0.6561 | 0.0056 |
| FGF2 | 108 | 0.6561 | 0.0051 |
| MAPK14 | 107 | 0.6523 | 0.0050 |
| IL4 | 106 | 0.6504 | 0.0058 |
| CCL2 | 105 | 0.6486 | 0.0046 |
| PIK3CA | 104 | 0.6486 | 0.0083 |
| IL2 | 103 | 0.6467 | 0.0046 |
| MMP2 | 101 | 0.6431 | 0.0042 |
| STAT1 | 99 | 0.6394 | 0.0044 |
| JAK2 | 98 | 0.6359 | 0.0044 |
| CXCR4 | 96 | 0.6341 | 0.0042 |
| SIRT1 | 95 | 0.6323 | 0.0045 |
| RHOA | 93 | 0.6271 | 0.0042 |
| MDM2 | 92 | 0.6271 | 0.0049 |
| KDR | 92 | 0.6271 | 0.0036 |
| MAP2K1 | 92 | 0.6253 | 0.0035 |
| PIK3R1 | 91 | 0.6253 | 0.0051 |
| RELA | 91 | 0.6236 | 0.0037 |
| CREB1 | 90 | 0.6219 | 0.0038 |
| GRB2 | 88 | 0.6202 | 0.0046 |
| EP300 | 84 | 0.6135 | 0.0046 |
| CAT | 76 | 0.5989 | 0.0041 |
| **Average** | 62.81 | 0.5812 | 0.0033 |

Table S7. Nodes and edges in the TCM-herb-ingredient-target-pathway network.

|  | Node | Node name | Related edges |
| --- | --- | --- | --- |
| TCM (to Herb) | 1 | KC | 4 |
| Herb (to Ingredient) | 4 | TH | 15 |
|  |  | EB | 16 |
|  |  | CC | 7 |
|  |  | LB | 1 |
| Ingredient (to Target) | 35 | proline | 1 |
|  |  | valine | 2 |
|  |  | betaine | 1 |
|  |  | Epigallocatechin | 2 |
|  |  | catechin | 1 |
|  |  | magnoflorine | 9 |
|  |  | L-epicatechin | 8 |
|  |  | hyperoside | 9 |
|  |  | baohuosu | 8 |
|  |  | daidzein | 15 |
|  |  | triptolide | 12 |
|  |  | quercetin | 25 |
|  |  | luteolin | 20 |
|  |  | epimedin A | 11 |
|  |  | epimedin B | 10 |
|  |  | epimedin C | 9 |
|  |  | icariin | 11 |
|  |  | kaempferol | 12 |
|  |  | isorhamnetin | 9 |
|  |  | ikarisoside A | 10 |
|  |  | tripterifordin | 8 |
|  |  | sagittatoside B | 10 |
|  |  | rha-icariside | 9 |
|  |  | icaritin | 9 |
|  |  | icarisid | 9 |
|  |  | wilfortrine | 8 |
|  |  | peritassine A | 8 |
|  |  | wilfordine | 10 |
|  |  | wilfornine D | 5 |
|  |  | wilforgine | 10 |
|  |  | triptophenolide | 5 |
|  |  | euonine | 8 |
|  |  | wilfornine A | 8 |
|  |  | wilforine | 7 |
|  |  | celastrol | 12 |
| Target (to Pathway） | 45 | TP53 | 2 |
|  |  | AKT1 | 8 |
|  |  | VEGFA | 3 |
|  |  | MAPK3 | 9 |
|  |  | MYC | 3 |
|  |  | IL6 | 6 |
|  |  | EGFR | 3 |
|  |  | MAPK1 | 9 |
|  |  | HRAS | 5 |
|  |  | EGF | 3 |
|  |  | CCND1 | 2 |
|  |  | FN1 | 1 |
|  |  | MTOR | 3 |
|  |  | HSP90AA1 | 3 |
|  |  | ERBB2 | 2 |
|  |  | BCL2L1 | 2 |
|  |  | FGF2 | 2 |
|  |  | IL4 | 5 |
|  |  | PIK3CA | 7 |
|  |  | IL2 | 4 |
|  |  | JAK2 | 3 |
|  |  | MDM2 | 1 |
|  |  | KDR | 3 |
|  |  | MAP2K1 | 7 |
|  |  | PIK3R1 | 7 |
|  |  | RELA | 8 |
|  |  | CREB1 | 3 |
|  |  | GRB2 | 5 |
|  |  | CASP3 | 3 |
|  |  | TNF | 6 |
|  |  | JUN | 7 |
|  |  | MAPK8 | 7 |
|  |  | MMP9 | 2 |
|  |  | PTGS2 | 3 |
|  |  | IL1B | 6 |
|  |  | FOS | 7 |
|  |  | MAPK14 | 8 |
|  |  | CCL2 | 2 |
|  |  | IL10 | 2 |
|  |  | RHOA | 1 |
|  |  | CXCL8 | 2 |
|  |  | STAT3 | 2 |
|  |  | STAT1 | 4 |
|  |  | EP300 | 1 |
|  |  | SRC | 1 |
| Pathway (to Target) | 10 | PI3K-Akt signaling pathway | 28 |
|  |  | TNF signaling pathway | 19 |
|  |  | T cell receptor signaling pathway | 18 |
|  |  | IL-17 signaling pathway | 17 |
|  |  | JAK-STAT signaling pathway | 19 |
|  |  | MAPK signaling pathway | 22 |
|  |  | Toll-like receptor signaling pathway | 16 |
|  |  | Th17 cell differentiation | 16 |
|  |  | Osteoclast differentiation | 16 |
|  |  | VEGF signaling pathway | 12 |
